# Investigations on Synperiplanar and Antiperiplanar Isomers of Losartan: Theoretical and Experimental NMR Studies

**DOI:** 10.3390/molecules200711875

**Published:** 2015-06-29

**Authors:** Jacek Kujawski, Kornelia Czaja, Tomasz Ratajczak, Elżbieta Jodłowska, Marcin K. Chmielewski

**Affiliations:** 1 Department of Organic Chemistry, Faculty of Pharmacy, Poznan University of Medical Sciences, Grunwaldzka 6 Street, 60-780 Poznań, Poland; E-Mails: czaja.kornelia@gmail.com (K.C.); youleadmeastray@gmail.com (E.J.); 2 Institute of Bioorganic Chemistry Polish Academy of Sciences, Z. Noskowskiego Street 12/14, 61-704 Poznań, Poland; E-Mail: tomaszr@ibch.poznan.pl

**Keywords:** losartan, NMR calculations, DFT, MP2

## Abstract

Losartan inhibits the renin-angiotensin-aldosterone system by blocking the angiotensin II receptor. It is commonly used in cardiovascular diseases, such as hypertension. Several publications applied the *ab initio* and density functional theory methods to investigate the molecule of losartan. Only in one of them were the nuclear magnetic resonance spectra calculations carried out, and their results were correlated with the experimental values. The authors focused their attention on calculations of the anion form of losartan, taking into consideration both its synperiplanar and antiperiplanar configurations. Coefficients of determination and mean absolute deviation parameters were calculated for the experimental and calculated chemical shifts for every used basis set. They showed a noticeably stronger correlation for the *anti*-isomers than for the *syn*-isomers. Moreover, the solvation model increased the value of this parameter. The results of calculations confirmed that an *anti*-conformation of the analyte seems to be the preferred one, and such an orientation might be most potent within the receptor cavity, which is in agreement with the results of previous studies.

## 1. Introduction

Losartan is a drug that inhibits the renin-angiotensin-aldosterone (RAA) system by blocking the angiotensin II receptor. Thus, it is commonly used in cardiovascular diseases, such as hypertension. Angiotensin II receptor-1 antagonists (ARBs) reveal fewer side effects than angiotensin-converting enzyme inhibitors (ACEI), as they do not block the metabolism of bradykinins. In turn, they rarely cause angioedema or dry cough. Losartan was the first ARB-type drug available on the market and was accepted for clinical use in 1995 [1]. The increasing prevalence of cardiovascular diseases results in a greater popularity of drugs that block the RAA system. Therefore, it is important to thoroughly investigate the properties of the currently used ARBs and to draw conclusions concerning their affinity to the receptor and the efficiency of blocking it. The *in vivo* losartan hydroxyl group is oxidized to a carboxylic moiety through an aldehyde intermediate by cytochrome P-450 [2]. This metabolite has a relatively long time of action and a considerably greater affinity to the receptor, which lengthens the activity of losartan despite the short half-life of losartan itself [3,4]. Nevertheless, both losartan and its derivative bind with the enzyme, and both were investigated in docking studies in their antiperiplanar form [5].

The experimental analyses carried out previously showed that losartan prefers an *anti* conformation of the tetrazole and imidazole moieties with relation to the phenyl ring [6]. The results of the NOESY approach indicated the proximity of *n*-butyl to both the methylene moiety and the phenyl ring [6]. In 2014, there were two publications addressed the *ab initio* and density functional theory (DFT) methods to investigate the molecule of losartan for the first time [7,8]. Only one of them included the ^1^H- and ^13^C-NMR calculations (the Gaussian 03W program at the B3LYP/6-31G(d,p) level of theory) and their correlation with the experimental values [7]. However, the calculations were not carried out using the polarizable continuum model (PCM), so the solvent was not taken into consideration, and no other basis sets were used. The authors focused primarily on carbon atoms and did not discuss the values acquired for particular hydrogen atoms in detail. The authors described the discrepancies between the calculated and experimental results; however, calculations were carried out for an isolated losartan molecule in the gaseous phase. Continuing our computational chemistry investigations on biologically-active azoles [9,10,11], we focused our attention on calculation of the chemical shifts of losartan (^1^H-NMR spectrum) applying different basis sets and methods in the gaseous phase and using the conductor-like polarizable continuum model (CPCM) solvation model, so that the influence of the solvent environment is also accounted for. Apart from the DFT formalism, losartan was also investigated using molecular dynamics (MD) techniques [5,12]. The main goal of our investigations was to prove what basis sets or methods yield results in the best agreement with the experimental data. Moreover, we hoped to explain which conformation of the analyte is preferred from the standpoint of quantum chemistry.

## 2. Results and Discussion

**Figure 1:**
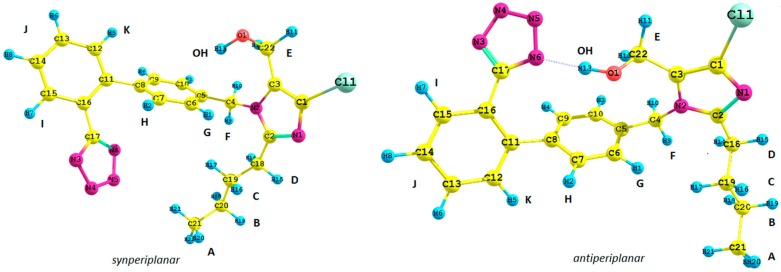
Structure of possible isomers of the losartan anion (**1**): antiperiplanar conformation (*anti*) and synperiplanar conformation (*syn*).

Continuing our investigations on the interactions of biologically-important azahetarenes with the environment, we focused our attention on losartan potassium salt. The present study deals, *inter alia*, with simulation of the ^1^H-NMR spectrum of losartan in its anion form (**1** given in Figure 1).

**Scheme 1:**
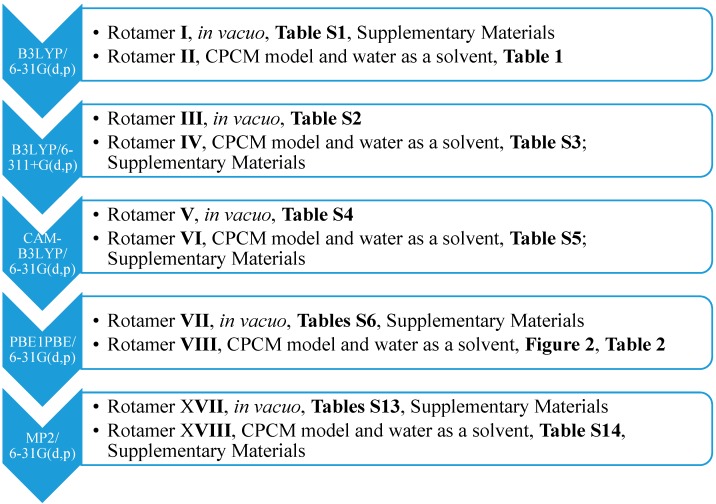
The approaches used for the optimization together with the numbers of *syn*-rotamers. CPCM, the conductor-like polarizable continuum model.

Basing on the already published reports [7,8,12], we decided to carry out a simulation of the ^1^H-NMR spectrum of anion **1**. We took into consideration the position of the tetrazole ring and the butyl chain connected with the imidazole ring with respect to the biphenyl moiety of **1**, *i.e.*, its synperiplanar (*syn*) and antiperiplanar (*anti*) isomers. Previously reported studies concerning the spectroscopic analysis of losartan require a brief commentary. Generally, the results are difficult to emulate due to the lack of supplementary materials [7,8]; they were just a repetition of previous findings [8]; or only one functional was used for calculations [7,12]. In our paper, we generated the theoretical ^1^H-NMR spectrum for all of the most stable *syn* rotamers of **1** using the gauge-including atomic orbital (GIAO) method [13] implemented in Gaussian G09 D.01 (Gaussian, Inc.: Wallingford, CT, USA, 2009). Therefore, we carried out the optimization of particular rotamers and NMR spectrum generation at the same level of theory. To show the results more clearly, only one most stable rotamer was considered with the lowest energy, optimized at the (1) B3LYP/6-31G(d,p), (2) B3LYP/6-311+G(d,p), (3) CAM-B3LYP/6-31G(d,p), (4) PBE1PBE/6-31G(d,p) and (5) MP2/6-31G(d,p) level of theory, both in the gaseous phase, using the conductor-like polarizable continuum model (CPCM) solvation model and water as a solvent (Scheme 1).

**Figure 2:**
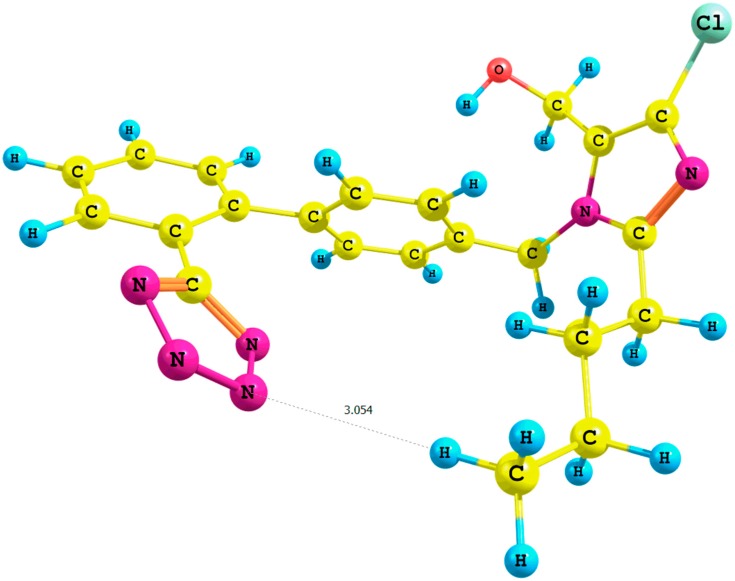
Optimized structure of the *syn*-losartan anion **1** (PBE1PBE/6-31G(d,p) level of theory, Rotamer **VIII**, CPCM solvation model).

The calculated values showed a strong correlation with the NMR experimental data for Compound **1**. The highest relative percentage error was observed for the protons of the hydroxyl and methyl functionality (A) as a part of the *n*-butyl chain. This is due to the lability of the hydroxyl proton, the shielding of the methyl protons and the proximity of the tetrazole ring. The distance between the methyl protons of the butyl chain and the N2 nitrogen of the tetrazole ring for Rotamers **II**, **IV**, **VI**, **VIII** (Figure 2) equaled after optimization approximately 3 Å (CPCM model, Figure 2).

Moreover, the application of the CPCM solvation model for *syn* isomers **1** (Rotamers **II**, **IV**, **VI**, **VIII**; Table 1 and Table 2) resulted in a considerable improvement of the relative percentage errors of the chemical shifts of the methyl protons A (93%, 16%, 21% and 14%; Table 1, Table 2, Tables S3 and S5, respectively) when compared to the rotamers optimized in the gaseous phase (Rotamers **I**, **III**, **V**, **VII**, respectively). The relative percentage error of the chemical shift of these protons equaled: 93%, 86%, 100% and 94% (gaseous phase; Tables S1, S2, S4 and S6, respectively). Such improvement was particularly noticeable for rotamers calculated using the hybrid functional PBE1PBE (PBE0 functional).

**Table 1:** Calculated and experimental data recorded at 293 K for losartan anion (**1**) Rotamer **II** (isomer *syn*); the following parameters were determined for their proton groups: experimental (Exp.) and calculated values of the chemical shifts (II), absolute errors (δ2) and values of the relative percentage errors (E); calculated NMR shielding for proton H^ref^ = 31.740 ppm for TMS (tetramethylsilane) (B3LYP/6-31G(d,p)/gauge-including atomic orbital (GIAO)/CPCM; *R*^2^ = 0.73, mean absolute deviation (MAD) = 0.58.

Proton Signals	Exp.	Water		
		II	δ2	E
A	0.826	1.592	0.766	93
B	1.270	1.278	0.008	1
C	1.497	1.718	0.221	15
D	2.515	2.407	0.108	4
E	4.328	4.386	0.058	1
OH	5.304	0.263	5.041	95
F	5.228	5.010	0.217	4
G	6.917	6.881	0.035	1
H	7.108	7.473	0.364	5
I	7.553	8.966	1.413	19
J	7.370	6.883	0.487	7
K	7.293	6.836	0.458	6

**Table 2:** Calculated and experimental data recorded at 293 K for losartan anion (**1**) Rotamer **VIII** (isomer syn); the following parameters were determined for their proton groups: experimental (Exp.) and calculated values of the chemical shifts (VIII), absolute errors (δ8) and values of the relative percentage errors (E); calculated NMR shielding for proton H^ref^ = 31.642 ppm for TMS (PBE1PBE/6-31G(d,p)/GIAO/CPCM; *R*^2^ = 0.75, MAD = 0.77.

Proton Signals	Exp.	Water		
		VIII	δ8	E
A	0.826	0.940	0.114	14
B	1.270	1.367	0.097	8
C	1.497	1.311	0.186	12
D	2.515	2.599	0.083	3
E	4.328	4.418	0.089	2
OH	5.304	0.214	5.090	96
F	5.228	5.239	0.011	0
G	6.917	7.269	0.352	5
H	7.108	7.439	0.331	5
I	7.553	8.154	0.601	8
J	7.370	7.712	0.342	5
K	7.293	7.617	0.323	4

Herein, we focused our attention only on the electrostatic interactions between the solute and water as the solvent (in order to simulate the cellular environment). These are in fact the interactions that generally dominate solvent effects, especially in polar solvents (*i.e.*, water). As a matter of fact, continuum models were originally developed to describe this type of interaction [14].

**Figure 3:**
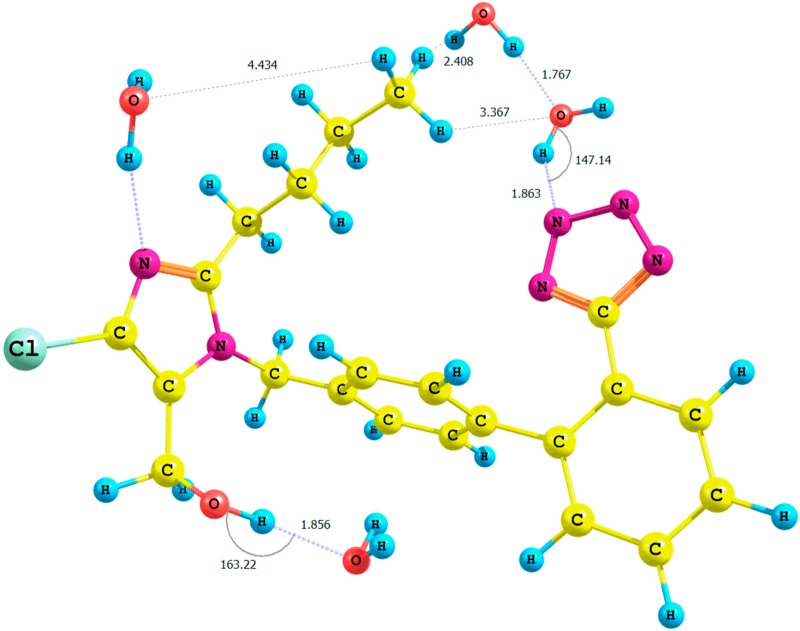
Optimized structure of the *syn*-losartan anion cluster **1** with water molecules (Rotamer **X**); interaction of **1** with three water molecules (PBE1PBE/6-31G(d,p) level of theory, gas).

The optimization of clusters of the *syn* isomers **1** with water molecules resulted in a considerable decrease of the relative percentage error of the chemical shifts of A protons and the hydroxyl moiety in the *in silico* ^1^H-NMR spectrum. During the optimization of the **IX** cluster (B3LYP/6-31G(d,p)/gas level of theory; Table S7, Supplementary Materials), we observed a migration of three water molecules surrounding the methyl group A to the tetrazole ring. Concurrently, the distance *n*-butyl–N5_terazole_ within the **X** cluster (Figure 3, PBE1PBE/6-31G(d,p)/gas level of theory) decreased from 2.9 to 2.5 Å in comparison with the corresponding **VII** rotamer (PBE1PBE/6-31G(d,p)/gas level of theory; the numeration of nitrogen atoms is taken from the output files, and the visualization of all rotamers is given in the Supplementary Materials).

This caused a decrease in relative percentage errors of the methyl group from 93% (Rotamer **I**) to 43% (Cluster **IX**) calculated using the B3LYP functional in the gaseous phase and from 94% (Rotamer **VII**) to 50% (Rotamer **X**) estimated applying the PBE0 functional in the gaseous phase. A similar trend was observed for the hydroxyl proton in terms of the values of its chemical shift. We noticed a decline in the relative percentage errors within the results of calculations using the PBE0 functional in the gaseous phase: from 95% (Rotamer **I**, B3LYP functional) to 27% (Cluster **IX**, B3LYP functional) in the gaseous phase and from 94% (Rotamer **VII**, PBE0 functional) to 30% (Cluster **X**, PBE0 functional).

**Scheme 2:**
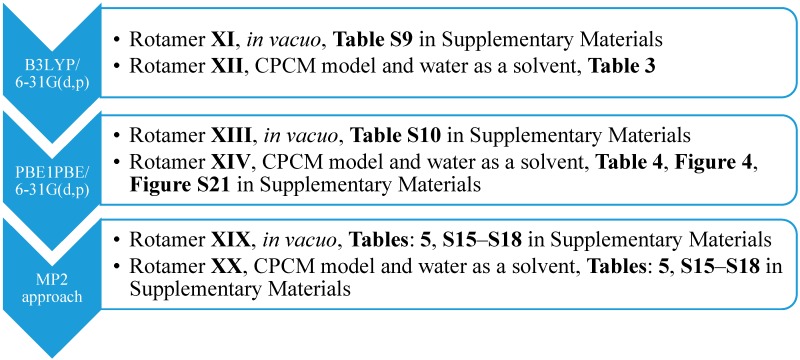
The approaches used for the optimization together with the numbers of *anti*-rotamers.

Subsequently, we used an analogous methodology for the antiperiplanar (*anti*) rotamers of **1** (Figure 1). The *anti*-rotamers were obtained by rotating the bonds C17–C16, C11–C8, C5–C4, C4–N2, C3–C22 and C2–C18 (**1** in Figure 1) in torsion angle increments of 20°. Then, they were optimized at the DFT or MP2 level of theory using the Gaussian 09 D.01 program (Scheme 2), namely: (1) B3LYP/6-31G(d,p) and (2) PBE1PBE/6-31G(d,p) basis set and the MP2 approach in the gaseous phase and applying the CPCM solvation model (water as a solvent).

**Figure 4:**
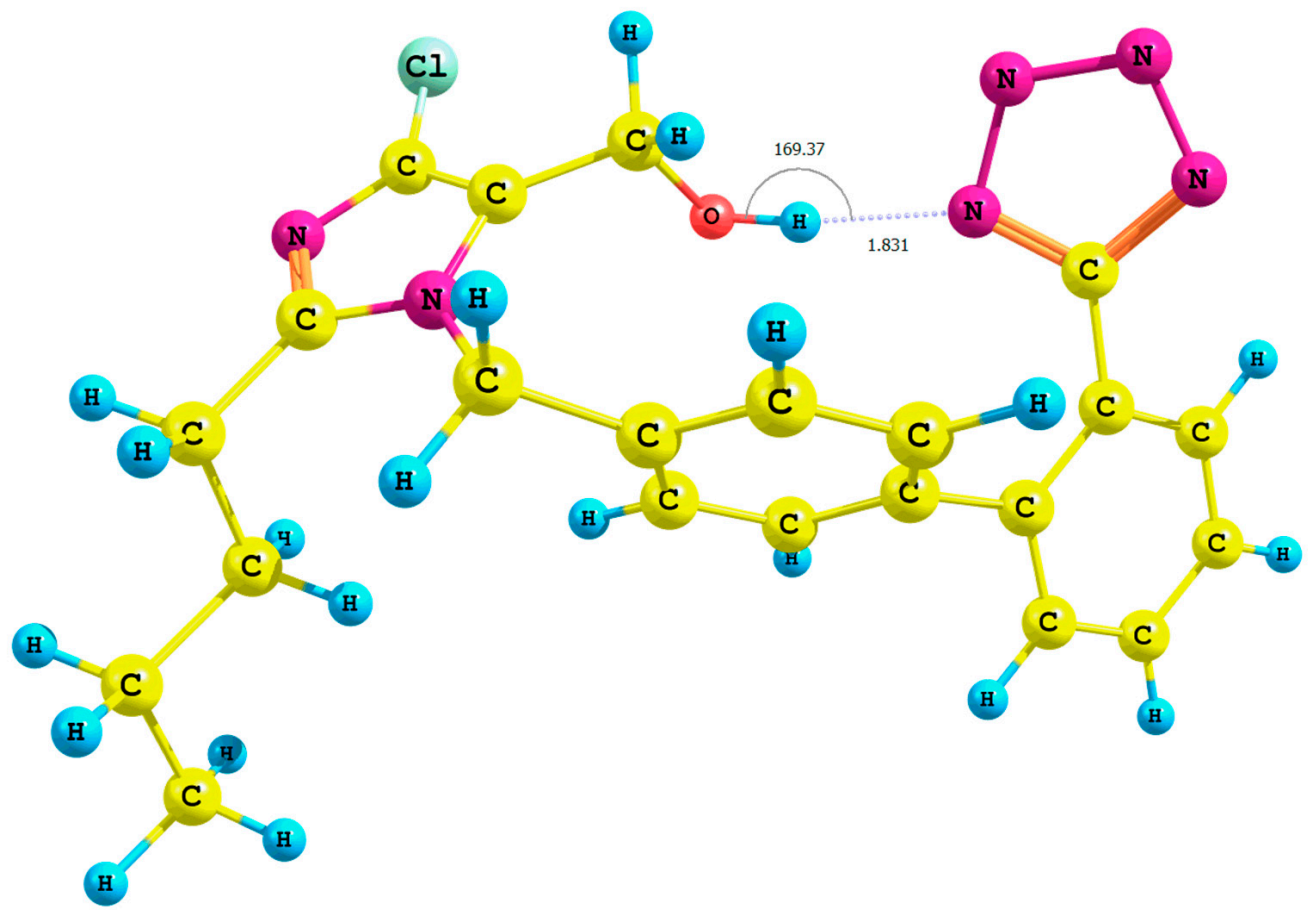
Optimized structure of the *anti*-losartan anion **1** (PBE1PBE/6-31G(d,p) level of theory, Rotamer **XIV**, CPCM solvation model).

Analogously, we calculated NMR shielding for TMS proton (H^ref^) at the (1) B3LYP/6-31G(d,p), (2) PBE1PBE/6-31G(d,p) or (3) MP2/6-31G(d,p) level of theory (both gaseous phase and the CPCM solvation model with water as a solvent). The application of B3LYP/6-311+G(d,p) and CAM-B3LYP functionals for the *syn* isomers **1** did not result in a significant improvement of the agreement between the calculated and the experimental values of chemical shifts in the ^1^H-NMR spectrum. Therefore, the calculations of the *anti-*isomers **1** were limited to the two abovementioned functionals. In this case, the application of CPCM solvation model resulted in a decline in the relative percentage error of the hydroxyl proton from 27% (*anti*-isomer, B3LYP/6-31G(d,p)/gas level of theory, Rotamer **XI**; Table S9) to 7% (*anti*-isomer, B3LYP/6-31G(d,p)/CPCM level of theory, Rotamer **XII**; Table 3) or from 31% (*anti*-isomer, PBE1PBE/6-31G(d,p)/gas level of theory, Rotamer **XIII**; Table S10) to 12% (*anti*-isomer, PBE1PBE/6-31G(d,p)/CPCM level of theory, Rotamer **XIV**; Table 4; Figure 4; Figure S21 in the Supplementary Materials).

**Table 3:** Calculated and experimental data recorded at 293 K for losartan anion (**1**) Rotamer **XII** (isomer *anti*); the following parameters were determined for their proton groups: experimental (Exp.) and calculated values of the chemical shifts (XII), absolute errors (δ12) and values of the relative percentage errors (E); calculated NMR shielding for proton H^ref^ = 31.740 ppm for TMS (B3LYP/6-31G(d,p)/GIAO/CPCM; *R*^2^ = 0.99, MAD = 0.28.

Proton Signals	Exp.	Water		
		XII	δ12	E
A	0.826	1.038	0.212	26
B	1.270	1.549	0.279	22
C	1.497	1.794	0.298	20
D	2.515	2.658	0.143	6
E	4.328	4.270	0.058	1
OH	5.304	5.699	0.395	7
F	5.228	4.986	0.242	5
G	6.917	7.482	0.566	8
H	7.108	7.261	0.153	2
I	7.553	8.145	0.592	8
J	7.370	7.566	0.197	3
K	7.293	7.519	0.226	3

**Table 4:** Calculated and experimental data recorded at 293 K for losartan anion (**1**) Rotamer **XIV** (isomer *anti*); the following parameters were determined for their proton groups: experimental (Exp.) and calculated values of the chemical shifts (XIV), absolute errors (δ14) and values of the relative percentage errors (E); calculated NMR shielding for proton H^ref^ = 31.642 ppm for TMS (PBE1PBE/6-31G(d,p)/GIAO/CPCM; *R*^2^ = 0.99, MAD = 0.37.

Proton Signals	Exp.	Water		
		XIV	δ14	E
A	0.826	1.030	0.204	25
B	1.270	1.509	0.239	19
C	1.497	1.805	0.309	21
D	2.515	2.676	0.161	6
E	4.328	4.270	0.058	1
OH	5.304	5.955	0.651	12
F	5.228	5.053	0.175	3
G	6.917	7.640	0.723	10
H	7.108	7.421	0.313	4
I	7.553	8.356	0.803	11
J	7.370	7.741	0.371	5
K	7.293	7.698	0.405	6

It is important to emphasize the influence of tetrazole nitrogen atoms and their distance to the hydroxyl group. The difference of the N6_tetrazole_–HO distance after optimization of the **XI** and **XII** or **XIII** and **XIV** rotamers was equal to 0.04 Å (numeration of nitrogen atoms is taken from the output files, and the visualization of all rotamers is given in the Supplementary Materials). A greater proximity of the tetrazole to the hydroxyl in the optimized **XI** or **XIII** rotamers (gaseous phase) intensified the polarization of the O-H bond and caused the deshielding of the proton. These changes were reflected by the higher relative percentage error of its chemical shift in the theoretical NMR spectrum. However, an analogous influence of the solvation model using B3LYP and PBE0 functionals for the *anti-*isomers **1** was not observed for the methyl protons (A) (Table 5). Instead, we noted an insignificant increase of the relative percentage error: from 20% (*anti*-isomer, B3LYP/6-31G(d,p)/gas level of theory, Rotamer **XI**; Table S9) to 26% (*anti*-isomer, B3LYP/6-31G(d,p)/CPCM level of theory, Rotamer **XII**; Table 3) or from 18% (*anti*-isomer, PBE1PBE/6-31G(d,p)/gas level of theory, Rotamer **XIII**; Table S10) to 25% (*anti*-isomer, PBE1PBE/6-31G(d,p)/CPCM Rotamer **XIV**; Table 4; Figure 4). The changes of the relative percentage error of hydroxyl and methyl (A) protons are gathered in Table 5.

Moreover, the interaction of N6_tetrazole_ and the hydroxyl moiety seems to be confirmed by the experimentally recorded NMR spectra of **1** at different temperatures (Figure S23, Supplementary Materials) and concentrations (Figure S25, Supplementary Materials). Based on the literature data, it is known that the shifts are mightily affected by hydrogen bonding, with large downfield shifts of H-bonded functionalities compared to the hydroxyl substituent [15]. Moreover, intra- and inter-molecular types of hydrogen bonds can be easily discriminated by means of NMR spectroscopy, where only in the second case, the resonance frequencies of the OH group are concentration dependent [16]. Our experiments clearly have shown that the triplet relating to the OH group moves upfield together with increasing temperature, while the changes in concentration of **1** do not affect the chemical shift of the hydroxyl moiety. Basing on these facts, we confirmed the results of *in silico* calculations, where the OH group was engaged in the formation of the strong intramolecular hydrogen bond with the nitrogen atom in a tetrazole ring.

**Table 5:** Calculated relative percentage errors (E) of protons A and OH of losartan anion (**1**) rotamers recorded at 293 K and values of coefficient of determination (*R*^2^) and mean absolute deviation (MAD) parameters as a factor describing the correlation of ^1^H-NMR with experimental data; the following approaches were considered: (a) B3LYP/6-31G(d,p); (b) PBE1PBE/6-31G(d,p); (c) MP2/6-31G(d,p).

Protons	Relative Percentage Error (E) (%)											
	a				b				c			
	Vacuum		Water		Vacuum		Water		Vacuum		Water	
	I	XI	II	XII	VII	XIII	VIII	XIV	XVII	XIX	XVIII	XX
	*syn*	*anti*	*syn*	*anti*	*syn*	*anti*	*syn*	*anti*	*syn*	*anti*	*syn*	*anti*
A	93	20	93	26	94	18	14	25	94	18	28	28
OH	95	27	95	7	94	31	96	12	97	30	95	12
**Parameters**												
*R*^2^	0.71	0.96	0.73	0.99	0.72	0.95	0.75	0.99	0.72	0.97	0.75	0.99
MAD	0.76	0.40	0.58	0.28	0.77	0.43	0.63	0.37	0.67	0.34	0.65	0.36

The correlation analysis of the ^1^H-NMR spectra of the *anti*-isomers with the experimental data showed a stronger correlation for the *anti-*isomers optimized using the CPCM solvation model (proven by the higher values of the coefficient of determination *R*^2^ and lower values of the mean absolute deviation MAD) in comparison with the *syn* isomers (Table 5).

The results of the QTAIM (quantum theory of atoms-in-molecules) analysis [17,18] carried out using the AIMAll 14.11.23 software [19] (Figure S21 and Table S19 in the Supplementary Materials) suggested the interactions between the tetrazole ring and the hydroxyl proton within the **XIV** rotamer (isomer *anti*, PBE1PBE/6-31G(d,p)/CPCM level of theory; Figure 4), as well. The proposed model showed the existence of a bond critical point localized on the bond path between the interacting moieties N6_tetrazole_···HO–CH_2_–imidazole.

Therefore, it can be concluded that a lower value of the relative percentage error for hydroxyl and methyl (A) protons **1** were obtained for the *anti-*isomers. The only exception was the relative percentage error of methyl protons of *syn*/*anti* isomers, *i.e.*, **VIII** and **XV** rotamers, respectively, optimized using the PBE0 functional and the CPCM model. The reasons for this finding can be explained due to the considerable relaxation of the C-C bonds within the *anti-*isomer **1** and a possible rotation of the methyl group of the butyl chain. Such a phenomenon in this case was not limited by the heterocyclic moiety and, thus, lack of the shielding effect of the analyzed protons, which was observed in the *syn* isomers **1**. Moreover, contrary to the *anti-*isomers **1** (where the hydroxyl was stabilized by the nitrogen atom of the tetrazole ring by means of electrostatic interactions), for the *syn* isomers **1**, the hydroxyl moiety undergoes shielding due to the steric hindrance (phenyl moiety connected with the methylene bridge). We can conclude that from the stereochemical point of view, losartan preferentially adopts the *anti* form. The results of the calculated energy indicated that the *anti-*isomers of **1** were always characterized with lower energy (more negative) (Table S17, Supplementary Materials). Furthermore, the correlation analysis of the theoretical and experimental ^1^H-NMR spectra of the rotamers **1** indicates unambiguously the tendency for a stronger correlation being obtained for the *anti-*isomers (higher values of the coefficients of determination *R*^2^ and lower values of the mean absolute deviation MAD parameters; Table 1, Table 2, Table 3, Table 4, Table 5, and Tables S1–S15, Supplementary Materials) in comparison with the *syn* isomers.

**Figure 5:**
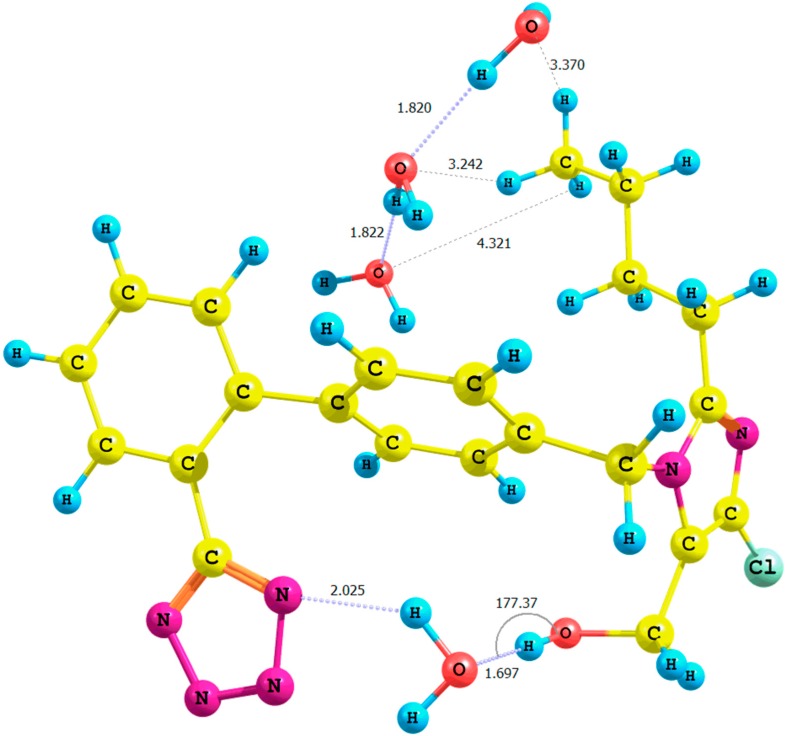
Optimized structure of the *anti*-losartan anion cluster **1** with water molecules (Cluster **XVI**); interaction of **1** with three water molecules (PBE1PBE/6-31G(d,p) level of theory, gas).

The application of the B3LYP or PBE0 functional for the *anti-*clusters of losartan surrounded by water molecules, optimized in the gaseous phase (Cluster **XV** and Cluster **XVI**; Figure 5, respectively), when compared to the single *anti*-isomers **1** (Rotamer **XI**, B3LYP/6-31G(d,p)/gas level of theory; Rotamer **XIII**, PBE1PBE/6-31G(d,p)/gas level of theory, respectively), did not result in an improvement of the relative percentage error of the chemical shift of the methyl (A) and hydroxyl protons.

Only when comparing the **XI** and **XV** clusters optimized in the gaseous phase at the B3LYP/6-31G(d,p) level of theory did we observe a small decline in the relative percentage error of the hydroxyl proton chemical shift: from 27% (Rotamer **XI**) to 25% (Rotamer **XV**).

In our study, we also compared the experimental values of chemical shifts with the calculated results obtained for the *syn*- and *anti*-romaters optimized using the MP2 method (MP2/6-31G(d,p) level of theory). For this purpose, we considered the gaseous phase (*syn*- and *anti*-isomers, Rotamers **XVII** and **XIX**, respectively) or the CPCM solvation model (water as a solvent, *syn*- and *anti*-isomers, Rotamers **XVIII** and **XX**, respectively). Calculated NMR chemical shifts for protons of these rotamers (MP2/6-31G(d,p)/GIAO approach) are gathered in Table 5 and Tables S15–S18 (Supplementary Materials). The obtained data showed a smaller value of the relative percentage error for the methyl (A) and hydroxyl protons of the *anti*-isomer of losartan in comparison with *syn*-isomers. Only for the methyl proton in the CPCM model did the relative percentage error for *syn*- and *anti*-isomers equal 28%. In contrast to our cost-effective DFT approach, the perturbation methods seem to be a too time consuming of an approach when used for calculating the ^1^H-NMR spectra of **1**. Nevertheless, the lower energy of the *anti-*isomers **1** (MP2 approach) also suggested their greater stability (Table S17, Supplementary Materials). Moreover, the MP2 method gave a better correlation between the calculated and experimental NMR chemical shifts for the *anti*-isomers (a higher value of the *R*^2^ and a lower value of the MAD parameters; Table 1, Table 2, Table 3, Table 4, Table 5, and Tables S1–S16, Supplementary Materials).

Our hypothesis that losartan Anion **1** adopts preferentially the *anti* form is in agreement with the literature data [6,12,20,21]. Molecular dynamics (MD) simulations in implicit solvation models supported the theoretical existence of the *anti* and *syn* conformations. On the other hand, an MD simulation run in the lipid bilayers revealed that losartan forms the hydrogen bonds with the lipid glycerol backbone and the phosphate groups [12]. The authors concluded that the *anti* conformation of losartan seems to be favored in the cellular environment, pointing to this isomer as the active one in a membrane environment. The results presented in our paper were also in accordance with the proposed two-step mechanism (involving lipid bilayers) or direct action of losartan in the AT1 receptor [21]. The results of energy, NMR, as well as QTAIM calculations presented herein proved that the *anti* conformation of **1** is most stable from the structural standpoint and allow us to assume that this orientation might be the most effective within the receptor cavity.

## 3. Experimental Section

The salt of losartan potassium **1** (1.2 mg, 0.26 µmol; Biofarm Sp. z o.o., Poznań, Poland) was added to 530 µL DMSO-*d*_6_ (99.8 atom%D, ARMAR Chemicals, Döttingen, Switzerland) and mixed for one hour at 293 K until the content was completely dissolved. The solution was moved to a 5-nm thin-wall tube with a final sample volume of 530 μL, and the 1D ^1^H-NMR spectra, as well as 2D homonuclear spectra were directly recorded starting from 293 K on a 400-MHz spectrometer (Bruker Daltonics, Billerica, MA, USA) operating at the frequency of 400 MHz (^1^H). Spectra were acquired within 4424.8-Hz spectral width and processed and prepared with TopSpin 3.0 Bruker software (Bruker BioSpin, Billerica, MA, USA). Density functional calculations were executed, and the geometries of losartan anion (**1**) were optimized at the DFT [22] level of theory (Becke three-term correlation functional; the Lee, Yang and Parr exchange functional B3LYP, or long range corrected version of the B3LYP functional, or the Perdew, Burke and Ernzerhof functional PBE1PBE) or applying the Mőller–Plesset second order perturbation theory MP2 using the Gaussian 09 D.01 program [23], namely the (1) B3LYP/6-31G(d,p) [24], (2) CAM-B3LYP/6-31G(d,p) [25], (3) B3LYP/6-311+G(d,p) [26,27], (4) PBE1PBE/6-31G(d,p) [28,29] and (5) MP2/6-31G(d,p) [30] approach in the gaseous phase and applying the conductor-like polarizable continuum model (CPCM, water as a solvent) [27,31,32]. The vibrational frequencies and thermodynamic properties were calculated by applying the ideal gas, rigid rotor and harmonic oscillator approximations, and the energy minimum was confirmed by the frequency calculation for all rotamers; no negative frequencies were detected in the generated vibrational spectrum of the analyzed rotamers. All of the *syn*-/*anti*-rotamers were obtained by rotating the bonds C17–C16, C11–C8, C5–C4, C4–N2, C3–C22 and C2–C18 (Figure 1) in torsion angle increments of 20° (changes in torsion angles are gathered in Table S18 given in the Supplementary Materials), and a total of 1080 rotamers were obtained. NMR shielding for proton (H^ref^) was calculated for TMS (tetramethylsilane) at the (1) B3LYP/6-31G(d,p), (2) CAM-B3LYP/6-31G(d,p), (3) B3LYP/6-311+G(d,p), (4) PBE1PBE/6-31G(d,p) and (5) MP2/6-31G(d,p) level of theory (gaseous phase and CPCM solvation model and water as solvent) at 293 K. It is noteworthy that the difference between dielectric constant values of water and DMSO are not significant (ε = 78.3 and 46.8, respectively [23]). Moreover, the results of the NMR calculations carried out using DMSO as a solvent in comparison with the corresponding data (water as a solvent) were very similar (Tables S1b and S6b for *syn*-isomers or Tables S9b and S10b for *anti*-isomers given in the Supplementary Materials). Particular clusters with water molecules were constructed by adding water molecules to protons of hydroxyl and methyl groups and then optimized. In order to discriminate the protons in **1**, they were marked according to Figure 1 positions (experimental values of chemical shifts are given in ppm and are in good agreement with reference data [10]; Figures S22–S24, Supplementary Materials): ^1^H-NMR (DMSO-*d*_6_, 400 MHz, 293 K) ppm: **I** 7.553 (H7; 1H; d; ^3^*J*_HH_ = 9.2 Hz), **J** 7.370 (H6, H8; 2H; m; overlapping signals), **K** 7.293 (H5; 1H; d; ^3^*J*_HH_ = 9.2 Hz), **H** 7.108 (H2, H4; 2H; d; ^3^*J*_HH_ = 8.0 Hz), **G** 6.917 (H1, H3; 2H; d; ^3^*J*_HH_ = 8.0 Hz), **F** 5.228 (H9, H10; 2H; s), **OH** 5.304 (H13, 1H, t, ^3^*J*_HH_ = 5.2 Hz), **E** 4.328 (H11, H12; 2H; d; ^3^*J*_HH_ = 4.8 Hz), **D** 2.51 (H14, H15; 2H, s), **C** 1.497 (H16, H17; 2H; m), **B** 1.270 (H19, H18; 2H; m), **A** 0.826 (H22–H20; 3H; t; ^3^*J*_HH_ = 7.6 Hz). The ^1^H-NMR spectra were recorded in DMSO-*d*_6_. For Losartan, the ^1^H-NMR spectrum was acquired within the 4424.8 Hz spectral width. A total of 128 scans was collected.

The compound of interest (**1**) and reference compound (TMS) were calculated using the same method, and the reference compound was used to obtain the chemical shifts of **1** according to the following equation: δ_i_ = σ_ref_ − σ_i_, where δ_i_ was the chemical shift of i-nuclei of **1** and σ_ref_ and σ_i_ were the calculated isotropic magnetic shielding tensor for the TMS and **1**, respectively [9,10,33]. The calculated chemical shifts for the homotopic protons were averaged. The Chemcraft 1.7 software was utilized for visualization of all optimized rotamers [34]. The calculations were carried out using resources provided by Poznan Supercomputing and Networking Center (Reef cluster), as well as the Wrocław Center for Networking and Supercomputing (Supernova and Bem clusters).

## 4. Conclusions

This study involves a calculated theoretical ^1^H-NMR spectrum of losartan potassium **1**, taking into consideration its synperiplanar and antiperiplanar configuration and its correlation with the corresponding experimental data. For the calculations, the DFT formalism and the Mőller–Plesset second order perturbation MP2 level of theory were used. Each rotamer was optimized in the gaseous phase and using the CPCM solvation model, and their clusters with water environment were optimized in the gaseous phase. The highest relative percentage error in the generated NMR spectrum (GIAO method) was observed for the hydroxyl and methyl (A) protons. For the *syn* isomers **1**, it was primarily caused by the steric hindrance from the tetrazole ring, the limited rotation of the butyl chain and the proximity of the phenyl ring to the hydroxyl group. For the *anti*-isomers, not only the influence of the tetrazole ring on the polarization of the hydroxyl functionality was essential (the N_tetrazole_···HO contact was confirmed basing on the results of QTAIM investigations, as well as NMR experiments), but also a smaller steric hindrance from the butyl moiety. In general, lower values of the relative percentage error of the calculated chemical shifts of methyl (A) protons were obtained for *anti*-isomers of **1** when compared to the corresponding *syn* isomers. Values of the coefficients of determination (*R*^2^) calculated for the theoretical chemical shifts of each rotamer related to the experimental value of chemical shifts have shown that they are noticeably lower for the *syn* ones. Moreover, the CPCM solvation model used for the NMR calculations increased the value of this parameter. A stronger correlation between the calculated and experimental NMR chemical shifts for the *anti*-isomers was also proven by their lower values of the MAD parameters. Thus, the computational methods showed a considerably superior correlation with the experimental results for the *anti*- than the *syn*-rotamers. The results of NMR experiments, supported by energy and QTAIM investigations, proved the intramolecular type of bond formation between the hydroxyl group and a tetrazole ring and confirmed that an *anti* conformation of **1** seems to be the preferred one. Thus, such an orientation might be most potent within the receptor cavity, which is in agreement with the results of previous studies [6,12,20,21].

## Supplementary Materials

Supplementary materials can be accessed at: http://www.mdpi.com/1420-3049/20/07/11875/s1.
