# Supplementary material for: Investigations on Synperiplanar and Antiperiplanar Isomers of Losartan: Theoretical and Experimental NMR Studies
Source: Molecules. 2015 Jun 29;20(7):11875–90. doi: 10.3390/molecules200711875 (PMC6332005; doi:10.3390/molecules200711875)
Supplement: Supplementary file 1 [file molecules-20-11875-s001.pdf]

Supplementary Materials

The NMR signals were calculated at 293 K. Experimental data are given in the experimental section.

Table S1. Calculated and experimental data for losartan anion (**1**) Rotamer **I** (isomer *syn*); the following parameters were determined for their proton groups: experimental (Exp.) and calculated values of the chemical shifts (I), absolute errors (δ1) and values of the relative percentage errors (E); calculated NMR shielding for proton H^{ref} = 31.755 ppm for TMS (B3LYP/6-31G(d,p)/GIAO/gas; R^2 = 0.71, MAD = 0.76.

**Proton Signals**	**Exp.**	**Vacuum**		
		**I**	**δ1**	**E**
A	0.826	1.592	0.766	93
B	1.270	1.278	0.008	1
C	1.497	1.718	0.221	15
D	2.515	2.407	0.108	4
E	4.328	4.386	0.058	1
OH	5.304	0.263	5.041	95
F	5.228	5.010	0.217	4
G	6.917	6.881	0.035	1
H	7.108	7.473	0.364	5
I	7.553	8.966	1.413	19
J	7.370	6.883	0.487	7
K	7.293	6.836	0.458	6

Table S1b. Calculated and experimental data for losartan anion (**1**) Rotamer **Ib** (isomer *syn*); the following parameters were determined for their proton groups: experimental (Exp.) and calculated values of the chemical shifts (Ib), absolute errors (δ1b) and values of the relative percentage errors (E); calculated NMR shielding for proton H^{ref} = 31.7396 ppm for TMS (B3LYP/6-31G(d,p)/GIAO/CPCM/DMSO; R^2 = 0.73, MAD = 0.58.

**Proton Signals**	**Exp.**	**DMSO**		
		**Ib**	**δ1b**	**E**
A	0.826	0.956	0.130	16
B	1.270	1.383	0.113	9
C	1.497	1.307	0.189	13
D	2.515	2.561	0.046	2
E	4.328	4.452	0.124	3
OH	5.304	0.031	5.273	99
F	5.228	5.211	0.017	0
G	6.917	7.098	0.181	3
H	7.108	7.278	0.169	2
I	7.553	7.966	0.413	5
J	7.370	7.531	0.161	2
K	7.293	7.417	0.124	2

Table S2. Calculated and experimental data for losartan anion (**1**) Rotamer **III** (isomer *syn*); the following parameters were determined for their proton groups: experimental (Exp.) and calculated values of the chemical shifts (III), absolute errors (δ3) and values of the relative percentage errors (E); calculated NMR shielding for proton H^{ref} = 31.984 ppm for TMS (B3LYP/6-311+G(d,p)/GIAO/gas; R^2 = 0.74, MAD = 0.74.

**Proton Signals**	**Exp.**	**Vacuum**		
		**III**	**δ3**	**E**
A	0.826	1.538	0.712	86
B	1.270	1.297	0.027	2
C	1.497	1.601	0.104	7
D	2.515	2.555	0.039	2
E	4.328	4.385	0.057	1
OH	5.304	0.455	4.849	91
F	5.228	5.140	0.087	2
G	6.917	8.003	1.086	16
H	7.108	7.470	0.362	5
I	7.553	8.811	1.257	17
J	7.370	7.158	0.212	3
K	7.293	7.218	0.075	1

Table S3. Calculated and experimental data for losartan anion (**1**) Rotamer **IV** (isomer *syn*); the following parameters were determined for their proton groups: experimental (Exp.) and calculated values of the chemical shifts (IV), absolute errors (δ4) and values of the relative percentage errors (E); calculated NMR shielding for proton H^{ref} = 31.975 ppm for TMS (B3LYP/6-311+G(d,p)/GIAO/CPCM; R^2 = 0.75, MAD = 0.65.

**Proton Signals**	**Exp.**	**Water**		
		**IV**	**δ4**	**E**
A	0.826	0.960	0.134	16
B	1.270	1.432	0.162	13
C	1.497	1.408	0.088	6
D	2.515	2.719	0.204	8
E	4.328	4.302	0.026	1
OH	5.304	0.196	5.108	96
F	5.228	5.200	0.028	1
G	6.917	7.327	0.410	6
H	7.108	7.451	0.343	5
I	7.553	7.951	0.398	5
J	7.370	7.810	0.440	6
K	7.293	7.751	0.458	6

Table S4. Calculated and experimental data for losartan anion (**1**) Rotamer **V** (isomer *syn*); the following parameters were determined for their proton groups: experimental (Exp.) and calculated values of the chemical shifts (V), absolute errors (δ5) and values of the relative percentage errors (E); calculated NMR shielding for proton H^{ref} = 31.775 ppm for TMS (CAM-B3LYP/6-31G(d,p)/GIAO/gas; R^2 = 0.72, MAD = 0.77.

**Proton Signals**	**Exp.**	**Vacuum**		
		**V**	**δ5**	**E**
A	0.826	1.654	0.828	100
B	1.270	1.246	0.024	2
C	1.497	1.708	0.212	14
D	2.515	2.390	0.125	5
E	4.328	4.363	0.035	1
OH	5.304	0.309	4.995	94
F	5.228	4.984	0.244	5
G	6.917	7.014	0.097	1
H	7.108	7.557	0.449	6
I	7.553	9.154	1.601	21
J	7.370	7.051	0.319	4
K	7.293	7.009	0.285	4

Table S5. Calculated and experimental data for losartan anion (**1**) Rotamer **VI** (isomer *syn*); the following parameters were determined for their proton groups: experimental (Exp.) and calculated values of the chemical shifts (VI), absolute errors (δ6) and values of the relative percentage errors (E); calculated NMR shielding for proton H^{ref} = 31.757 ppm for TMS (CAM-B3LYP/6-31G(d,p)/GIAO/CPCM; R^2 = 0.74, MAD = 0.63.

**Proton Signals**	**Exp.**	**Water**		
		**VI**	**δ6**	**E**
A	0.826	0.996	0.170	21
B	1.270	1.366	0.096	8
C	1.497	1.273	0.224	15
D	2.515	2.540	0.024	1
E	4.328	4.452	0.123	3
OH	5.304	0.005	5.299	100
F	5.228	5.182	0.046	1
G	6.917	7.188	0.271	4
H	7.108	7.373	0.265	4
I	7.553	8.038	0.484	6
J	7.370	7.680	0.310	4
K	7.293	7.576	0.283	4

Table S6. Calculated and experimental data for losartan anion (**1**) Rotamer **VII** (isomer *syn*); the following parameters were determined for their proton groups: experimental (Exp.) and calculated values of the chemical shifts (VII), absolute errors (δ7) and values of the relative percentage errors (E); calculated NMR shielding for proton Href = 31.665 ppm for TMS (PBE1PBE/6-31G(d,p)/GIAO/gas; R^2 = 0.72, MAD = 0.77.

Proton Signals	Exp.	Vacuum		
		VII	δ7	E
A	0.826	1.600	0.774	94
B	1.270	1.273	0.003	0
C	1.497	1.742	0.245	16
D	2.515	2.447	0.068	3
E	4.328	4.379	0.051	1
OH	5.304	0.338	4.966	94
F	5.228	5.078	0.149	3
G	6.917	7.086	0.169	2
H	7.108	7.677	0.569	8
I	7.553	9.195	1.641	22
J	7.370	7.080	0.290	4
K	7.293	7.037	0.257	4

Table S6b. Calculated and experimental data for losartan anion (**1**) Rotamer **VIIb** (isomer *syn*); the following parameters were determined for their proton groups: experimental (Exp.) and calculated values of the chemical shifts (VIIb), absolute errors (δ7b) and values of the relative percentage errors (E); calculated NMR shielding for proton H^{ref} = 31.6536 ppm for TMS (PBE1PBE/6-31G(d,p)/GIAO/CPCM/DMSO; R^2 = 0.74, MAD = 0.66.

Proton Signals	Exp.	DMSO		
		VIIb	δ7b	E
A	0.826	1.000	0.174	21
B	1.270	1.364	0.094	7
C	1.497	1.289	0.208	14
D	2.515	2.598	0.083	3
E	4.328	4.445	0.116	3
OH	5.304	0.122	5.182	98
F	5.228	5.282	0.054	1
G	6.917	7.260	0.343	5
H	7.108	7.458	0.349	5
I	7.553	8.209	0.656	9
J	7.370	7.710	0.340	5
K	7.293	7.608	0.315	4

Table S7. Calculated and experimental data for *syn*-losartan anion–water (**1**–water) Cluster **IX**; the following parameters were determined for their proton groups: experimental (Exp.) and calculated values of the chemical shifts (VII), absolute errors (δ9) and values of the relative percentage errors (E); calculated NMR shielding for proton H^{ref} = 31.755 ppm for TMS (B3LYP/6-31G(d,p)/GIAO/gas; R^2 = 0.95, MAD = 0.39.

**Proton Signals**	**Exp.**	**Vacuum**		
		**IX**	**δ9**	**E**
A	0.826	1.184	0.358	43
B	1.270	1.264	0.006	0
C	1.497	1.594	0.098	7
D	2.515	2.440	0.076	3
E	4.328	4.437	0.109	3
OH	5.304	3.883	1.421	27
F	5.228	4.740	0.487	9
G	6.917	6.990	0.074	1
H	7.108	7.342	0.234	3
I	7.553	8.818	1.265	17
J	7.370	7.075	0.295	4
K	7.293	6.994	0.299	4

Table S8. Calculated and experimental data for *syn*-losartan anion–water (**1**–water) Cluster **X**; the following parameters were determined for their proton groups: experimental (Exp.) and calculated values of the chemical shifts (XI), absolute errors (δ11) and values of the relative percentage errors (E); calculated NMR shielding for proton H^{ref} = 31.665 ppm for TMS (PBE1PBE/6-31G(d,p)/GIAO/gas; R^2 = 0.93, MAD = 0.48.

**Proton Signals**	**Exp.**	**Vacuum**		
		**XI**	**δ11**	**E**
A	0.826	1.235	0.409	50
B	1.270	1.111	0.159	13
C	1.497	1.801	0.304	20
D	2.515	2.251	0.264	11
E	4.328	4.294	0.034	1
OH	5.304	3.725	1.579	30
F	5.228	5.055	0.172	3
G	6.917	7.169	0.253	4
H	7.108	7.506	0.398	6
I	7.553	9.162	1.609	21
J	7.370	7.174	0.196	3
K	7.293	6.921	0.373	5

Table S9. Calculated and experimental data for losartan anion (**1**) Rotamer **XI** (isomer *anti*); the following parameters were determined for their proton groups: experimental (Exp.) and calculated values of the chemical shifts (XI), absolute errors (δ11) and values of the relative percentage errors (E); calculated NMR shielding for proton H^{ref} = 31.755 ppm for TMS (B3LYP/6-31G(d,p)/GIAO/gas; R^2 = 0.96, MAD = 0.40.

Proton Signals	Exp.	Vacuum		
		XI	δ11	E
A	0.826	0.990	0.164	20
B	1.270	1.413	0.143	11
C	1.497	1.810	0.314	21
D	2.515	2.494	0.021	1
E	4.328	4.388	0.060	1
OH	5.304	6.756	1.452	27
F	5.228	4.693	0.535	10
G	6.917	7.237	0.320	5
H	7.108	7.254	0.145	2
I	7.553	8.557	1.004	13
J	7.370	6.980	0.390	5
K	7.293	7.048	0.246	3

Table S9b. Calculated and experimental data for losartan anion (**1**) Rotamer **XIb** (isomer *anti*); the following parameters were determined for their proton groups: experimental (Exp.) and calculated values of the chemical shifts (XIb), absolute errors (δ11b) and values of the relative percentage errors (E); calculated NMR shielding for proton H^{ref} = 31.6536 ppm for TMS (B3LYP/6-31G(d,p)/GIAO/CPCM/DMSO; R^2 = 0.99, MAD = 0.28.

Proton Signals	Exp.	DMSO		
		XIb	δ11b	E
A	0.826	1.039	0.212	26
B	1.270	1.545	0.275	22
C	1.497	1.802	0.305	20
D	2.515	2.704	0.188	7
E	4.328	4.307	0.022	1
OH	5.304	5.972	0.668	13
F	5.228	5.071	0.157	3
G	6.917	7.668	0.752	11
H	7.108	7.433	0.325	5
I	7.553	8.378	0.825	11
J	7.370	7.739	0.369	5
K	7.293	7.680	0.387	5

Table S10. Calculated and experimental data for losartan anion (**1**) Rotamer **XIII** (isomer *anti*); the following parameters were determined for their proton groups: experimental (Exp.) and calculated values of the chemical shifts (XIII), absolute errors (δ13) and values of the relative percentage errors (E); calculated NMR shielding for proton H^{ref} = 31.665 ppm for TMS (PBE1PBE/6-31G(d,p)/GIAO/gas; R^2 = 0.95, MAD = 0.43.

**Proton Signals**	**Exp.**	**Vacuum**		
		**XIII**	**δ13**	**E**
A	0.826	0.972	0.146	18
B	1.270	1.392	0.121	10
C	1.497	1.817	0.320	21
D	2.515	2.532	0.016	1
E	4.328	4.406	0.078	2
OH	5.304	6.973	1.669	31
F	5.228	4.745	0.483	9
G	6.917	7.406	0.489	7
H	7.108	7.428	0.320	4
I	7.553	8.758	1.205	16
J	7.370	7.131	0.239	3
K	7.293	7.204	0.089	1

Table S10b. Calculated and experimental data for losartan anion (**1**) Rotamer **XIIIb** (isomer *anti*); the following parameters were determined for their proton groups: experimental (Exp.) and calculated values of the chemical shifts (XIIIb), absolute errors (δ13b) and values of the relative percentage errors (E); calculated NMR shielding for proton H^{ref} = 31.6536 ppm for TMS (PBE1PBE/6-31G(d,p)/GIAO/CPCM/DMSO; R^2 = 0.99, MAD = 0.37.

**Proton Signals**	**Exp.**	**DMSO**		
		**XIII**	**δ13**	**E**
A	0.826	1.039	0.212	26
B	1.270	1.545	0.275	22
C	1.497	1.802	0.305	20
D	2.515	2.704	0.188	7
E	4.328	4.307	0.022	1
OH	5.304	5.972	0.668	13
F	5.228	5.071	0.157	3
G	6.917	7.668	0.752	11
H	7.108	7.433	0.325	5
I	7.553	8.378	0.825	11
J	7.370	7.739	0.369	5
K	7.293	7.680	0.387	5

Table S11. Calculated and experimental data for *anti*-losartan anion–water (**1**–water) Cluster **XV**; the following parameters were determined for their proton groups: experimental (Exp.) and calculated values of the chemical shifts (XV), absolute errors (δ15) and values of the relative percentage errors (E); calculated NMR shielding for proton H^{ref} = 31.755 ppm for TMS (B3LYP/6-31G(d,p)/GIAO/gas; R^2 = 0.95, MAD = 0.47.

Proton Signals	Exp.	Vacuum		
		XV	δ15	E
A	0.826	1.092	0.266	32
B	1.270	1.321	0.051	4
C	1.497	1.367	0.130	9
D	2.515	3.271	0.756	30
E	4.328	4.452	0.123	3
OH	5.304	6.640	1.336	25
F	5.228	5.645	0.417	8
G	6.917	7.151	0.235	3
H	7.108	7.268	0.159	2
I	7.553	8.989	1.435	19
J	7.370	7.009	0.361	5
K	7.293	6.946	0.348	5

Table S12. Calculated and experimental data for *anti*-losartan anion–water (**1**–water) **XVI**; the following parameters were determined for their proton groups: experimental (Exp.) and calculated values of the chemical shifts (XVI), absolute errors (δ16) and values of the relative percentage errors (E); calculated NMR shielding for proton H^{ref} = 31.665 ppm for TMS (PBE1PBE/6-31G(d,p)/GIAO/gas; R^2 = 0.95, MAD = 0.62.

Proton Signals	Exp.	Vacuum		
		XVI	δ16	E
A	0.826	0.572	0.255	31
B	1.270	1.456	0.186	15
C	1.497	0.988	0.508	34
D	2.515	2.340	0.175	7
E	4.328	4.509	0.181	4
OH	5.304	7.381	2.077	39
F	5.228	5.035	0.193	4
G	6.917	7.945	1.029	15
H	7.108	7.554	0.446	6
I	7.553	9.309	1.756	23
J	7.370	7.262	0.108	1
K	7.293	7.771	0.478	7

Table S13. Calculated and experimental data for *anti*-losartan anion (**1**) Rotamer **XVII** (isomer *syn*); the following parameters were determined for their proton groups: experimental (Exp.) and calculated values of the chemical shifts (XVII), absolute errors (δ17) and values of the relative percentage errors (E); calculated NMR shielding for proton H^{ref} = 31.967 ppm for TMS (MP2/6-31G(d,p)/GIAO/gas; R^2 = 0.72, MAD = 0.67.

**Proton Signals**	**Exp.**	**Vacuum**		
		**XVII**	**δ19**	**E**
A	0.826	1.603	0.777	94
B	1.270	1.192	0.078	6
C	1.497	1.583	0.087	6
D	2.515	2.440	0.075	3
E	4.328	4.283	0.045	1
OH	5.304	0.166	5.138	97
F	5.228	5.092	0.136	3
G	6.917	7.102	0.186	3
H	7.108	7.514	0.406	6
I	7.553	8.554	1.000	13
J	7.370	7.238	0.132	2
K	7.293	7.305	0.011	0

Table S14. Calculated and experimental data for losartan anion (**1**) Rotamer **XVIII** (isomer *syn*); the following parameters were determined for their proton groups: experimental (Exp.) and calculated values of the chemical shifts (XVIII), absolute errors (δ18) and values of the relative percentage errors (E); calculated NMR shielding for proton H^{ref} = 31.957 ppm for TMS (MP2/6-31G(d,p)/GIAO/CPCM; R^2 = 0.75, MAD = 0.65.

**Proton Signals**	**Exp.**	**Vacuum**		
		**XVIII**	**δ18**	**E**
A	0.826	1.056	0.230	28
B	1.270	1.334	0.064	5
C	1.497	1.288	0.209	14
D	2.515	2.610	0.095	4
E	4.328	4.382	0.054	1
OH	5.304	0.248	5.056	95
F	5.228	5.271	0.043	1
G	6.917	7.261	0.345	5
H	7.108	7.435	0.327	5
I	7.553	7.935	0.382	5
J	7.370	7.850	0.480	7
K	7.293	7.845	0.552	8

Table S15. Calculated and experimental data for losartan anion (**1**) Rotamer **XIX** (isomer *anti*); the following parameters were determined for their proton groups: experimental (Exp.) and calculated values of the chemical shifts (XIX), absolute errors (δ19) and values of the relative percentage errors (E); calculated NMR shielding for proton H^{ref} = 31.967 ppm for TMS (MP2/6-31G(d,p)/GIAO/gas; R^2 = 0.97, MAD = 0.34.

Proton Signals	Exp.	Vacuum		
		**XIX**	**δ19**	**E**
A	0.826	0.972	0.146	18
B	1.270	1.327	0.057	5
C	1.497	1.804	0.307	21
D	2.515	2.523	0.008	0
E	4.328	4.327	0.001	0
OH	5.304	6.878	1.574	30
F	5.228	4.866	0.362	7
G	6.917	7.343	0.427	6
H	7.108	7.354	0.246	3
I	7.553	8.302	0.749	10
J	7.370	7.293	0.077	1
K	7.293	7.451	0.158	2

Table S16. Calculated and experimental data for losartan anion (**1**) Rotamer **XX** (isomer *anti*); the following parameters were determined for their proton groups: experimental (Exp.) and calculated values of the chemical shifts (XX), absolute errors (δ20) and values of the relative percentage errors (E); calculated NMR shielding for proton H^{ref} = 31.957 ppm for TMS (MP2/6-31G(d,p)/GIAO/CPCM; R^2 = 0.99, MAD = 0.36.

Proton Signals	Exp.	Vacuum		
		**XX**	**δ20**	**E**
A	0.826	0.972	0.146	18
B	1.270	1.327	0.057	5
C	1.497	1.804	0.307	21
D	2.515	2.523	0.008	0
E	4.328	4.327	0.001	0
OH	5.304	6.878	1.574	30
F	5.228	4.866	0.362	7
G	6.917	7.343	0.427	6
H	7.108	7.354	0.246	3
I	7.553	8.302	0.749	10
J	7.370	7.293	0.077	1
K	7.293	7.451	0.158	2

Table S17. Calculated energy of losartan anion (**1**) rotamers; the following approaches were considered: (a) B3LYP/6-31G(d,p); (b) PBE1PBE/6-31G(d,p); (c) MP2/6-31G(d,p).

Methods		Energy (kcal/mol)			
DFT	a	vacuum	**I**	*syn*	−1,076,542.05
			**XI**	*anti*	−1,076,546.45
		water	**II**	*syn*	−1,076,597.98
			**XII**	*anti*	−1,076,600.96
	b	vacuum	**VII**	*syn*	−1,075,543.42
			**XIII**	*anti*	−1,075,548.96
		water	**VIII**	*syn*	−1,075,601.04
			**XIV**	*anti*	−1,075,604.20
MP2	c	vacuum	**XVII**	*syn*	−1,071,129.32
			**XIX**	*anti*	−1,071,132.90
		water	**XVIII**	*syn*	−1,071,191.71
			**XX**	*anti*	−1,071,192.79

Table S18. Estimated changes in torsion angles for optimized rotamers; the following approaches were considered: (a) B3LYP/6-31G(d,p); (b) PBE1PBE/6-31G(d,p); (c) MP2/6-31G(d,p).

**Type of Torsion Angle**	**Torsion Angles (°)**											
	**a**				**b**				**c**			
	Vacuum		Water		Vacuum		Water		Vacuum		Water	
	**I**	**XI**	**II**	**XII**	**VII**	**XIII**	**VIII**	**XIV**	**XVII**	**XIX**	**XVIII**	**XX**
	syn	*anti*	*syn*	*anti*	*syn*	*anti*	*syn*	*anti*	*syn*	*anti*	*syn*	*anti*
C16–C17–N6	126.3	127.6	125.3	127.3	126.3	127.8	125.3	127.4	124.6	125.9	124.0	125.8
C8–C11–C12	117.7	118.7	118.1	118.7	117.6	118.8	118.4	118.9	119.9	120.4	119.8	120.1
C4–C5–C10	118.9	119.6	118.9	119.8	118.9	119.6	119.2	119.9	119.6	119,5	119.7	119.9
C4–N2–C3	125.3	127.0	125.2	126.6	125.3	126.6	125.0	126.2	124.9	125.9	124.7	125.7
C22–C3–N2	124.5	126.5	125.0	127.1	124.5	126.4	124.9	127.0	124.4	126.0	124.8	126.7
C18–C2–N1	123.6	122.8	123.7	123.2	123.7	123.1	123.9	123.5	124.4	123.7	124.4	124.0

Equation S1: Boltzmann Distribution Equation

$$\mathrm{N_i/N_{tot}} = \mathrm{e}^{-\Delta E/\mathrm{RT}} \sum_{k=1}^{\mathrm{N_{tot}}} \mathrm{e}^{-\Delta E_k/\mathrm{RT}}$$

where ΔE, the relative energy of the *i*-th conformer from the minimum energy conformer; ΔE_k, the relative energy of the *k*-th conformer from the minimum energy conformer; *R*, the molar ideal gas constant equal to 8.31 J/(mol·K); T, temperature in Kelvin.

Distribution (%):

syn-isomers:

I: 8.6 **II**: 8.9 **VII**: 9.0 **VIII**: 9.1 **XVII**: 9.7 **XVIII**: 10.0

anti-isomers:

XI: 15.3 **XII**: 16.0 **XIII**: 17.2 **XIV**: 17.4 **XIX**: 16.9 **XX**: 17.1

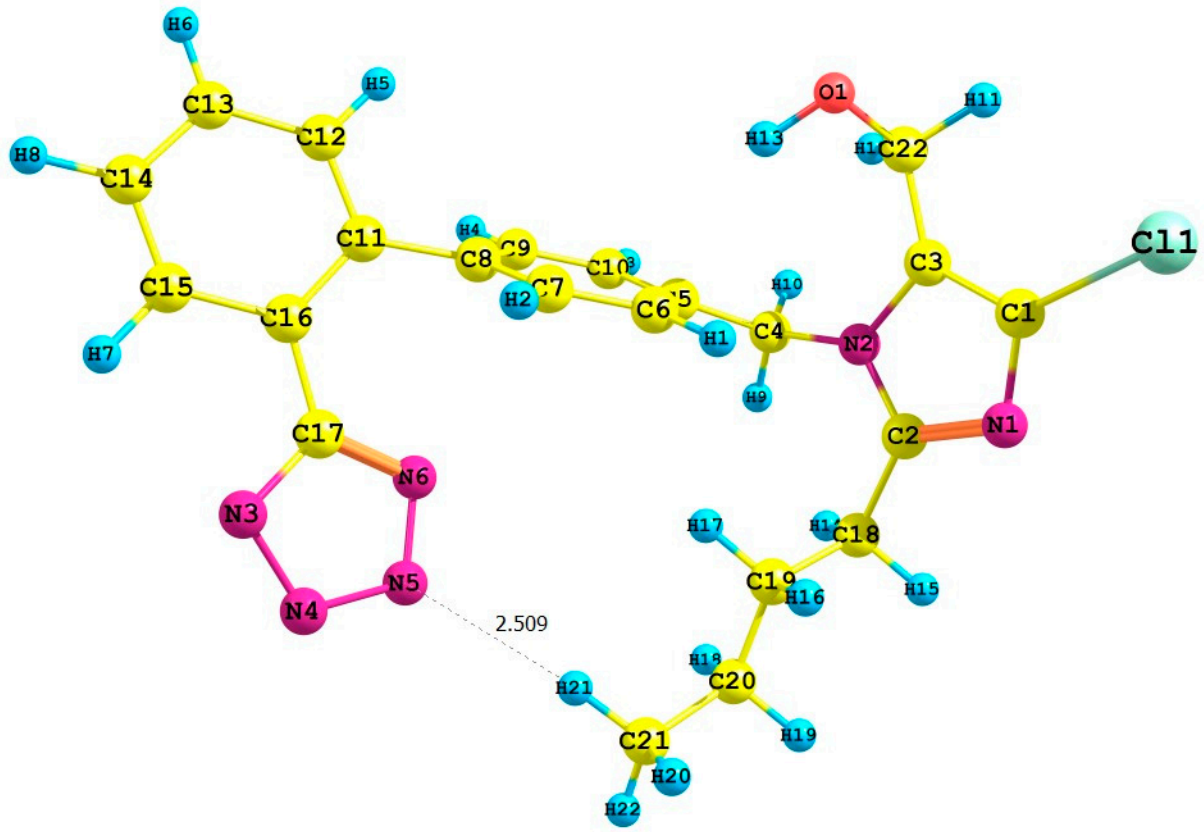

Figure S1. Optimized structure of the *syn*-losartan anion **1** (B3LYP/6-31G(d,p)/gas level of theory, Rotamer **I**).

Cartesian coordinates:

C	−4.562535000	−1.467681000	0.793771000
N	−4.739533000	−0.123499000	0.741346000
C	−3.931366000	0.291879000	−0.225854000
N	−3.253734000	−0.762041000	−0.792314000
C	−3.655861000	−1.924876000	−0.133509000
C	−2.313018000	−0.707371000	−1.906249000
C	−0.847646000	−0.852037000	−1.531558000
C	−0.379484000	−0.611677000	−0.232213000
C	0.986809000	−0.578046000	0.030725000
C	1.926942000	−0.783385000	−0.987729000
C	1.449833000	−1.091051000	−2.268913000
C	0.082859000	−1.123321000	−2.539278000
C	3.390484000	−0.713601000	−0.714465000
C	4.157463000	−1.861356000	−0.973033000
C	5.523241000	−1.909140000	−0.706148000
C	6.144557000	−0.780780000	−0.165150000
C	5.406475000	0.370838000	0.078871000
C	4.024538000	0.446404000	−0.194424000

C	3.353563000	1.730745000	0.048798000
N	3.934006000	2.722791000	0.759307000
N	3.050482000	3.722623000	0.733244000
N	1.981743000	3.351434000	0.026064000
N	2.144420000	2.100788000	−0.418337000
C	−3.767371000	1.732726000	−0.610404000
C	−2.567292000	2.446085000	0.052398000
C	−2.478781000	3.929077000	−0.329187000
C	−1.346328000	4.667108000	0.393339000
C	−3.116750000	−3.289183000	−0.419326000
O	−1.889811000	−3.601340000	0.233402000
Cl	−5.451734000	−2.452570000	1.926887000
H	−1.082502000	−0.409961000	0.569875000
H	1.333165000	−0.348689000	1.032107000
H	−0.260533000	−1.327480000	−3.551862000
H	2.161956000	−1.267669000	−3.069854000
H	3.653524000	−2.740167000	−1.368448000
H	6.089418000	−2.814758000	−0.907904000
H	5.876741000	1.260921000	0.482488000
H	7.208865000	−0.795790000	0.058319000
H	−2.464430000	0.256301000	−2.400940000
H	−2.596296000	−1.474158000	−2.636655000
H	−3.842218000	−4.019922000	−0.052728000
H	−3.032142000	−3.438350000	−1.508548000
H	−1.237765000	−2.931240000	−0.023303000
H	−3.697200000	1.835787000	−1.701443000
H	−4.693635000	2.232651000	−0.309954000
H	−2.667536000	2.349654000	1.140995000
H	−1.627860000	1.950705000	−0.217934000
H	−2.335241000	4.010073000	−1.416252000
H	−3.442920000	4.414159000	−0.112951000
H	−1.494779000	4.634199000	1.479674000
H	−0.361615000	4.234262000	0.178148000
H	−1.320782000	5.722580000	0.098557000

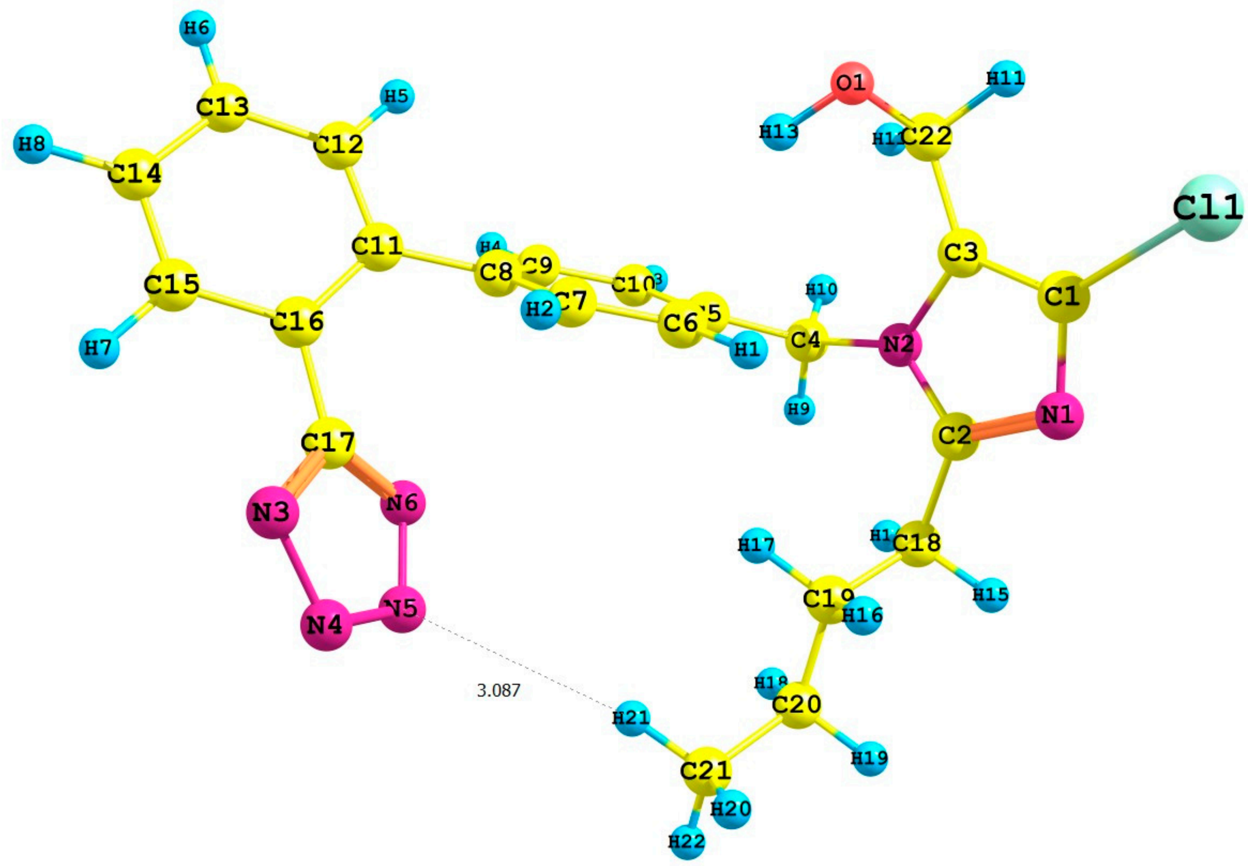

Figure S2. Optimized structure of the *syn*-losartan anion **1** (B3LYP/6-31G(d,p)/CPCM level of theory, Rotamer **II**).

Cartesian coordinates:

C	−4.426344000	−1.502317000	0.893953000
N	−4.695206000	−0.172463000	0.826701000
C	−3.952268000	0.276167000	−0.180226000
N	−3.223938000	−0.739789000	−0.747848000
C	−3.526523000	−1.915109000	−0.059046000
C	−2.321237000	−0.643736000	−1.893172000
C	−0.841708000	−0.723909000	−1.550583000
C	−0.350705000	−0.476596000	−0.261309000
C	1.021465000	−0.476501000	−0.011343000
C	1.945070000	−0.715768000	−1.038652000
C	1.445303000	−0.992673000	−2.320941000
C	0.074838000	−0.997570000	−2.573213000
C	3.410870000	−0.742864000	−0.766816000
C	4.125941000	−1.901595000	−1.113197000
C	5.482030000	−2.039918000	−0.822896000
C	6.151920000	−1.005621000	−0.167404000
C	5.464250000	0.159293000	0.163505000
C	4.099918000	0.322918000	−0.135384000

C	3.473986000	1.619824000	0.194233000
N	3.782116000	2.324944000	1.300813000
N	3.055804000	3.450460000	1.199939000
N	2.351883000	3.423739000	0.076047000
N	2.593953000	2.278083000	−0.582867000
C	−3.905053000	1.714560000	−0.601738000
C	−2.722627000	2.506262000	−0.003238000
C	−2.713964000	3.977688000	−0.432436000
C	−1.568796000	4.774864000	0.200554000
C	−2.919779000	−3.252934000	−0.336318000
O	−1.694104000	−3.497844000	0.361867000
Cl	−5.199827000	−2.516326000	2.086303000
H	−1.039318000	−0.278293000	0.553910000
H	1.379327000	−0.281799000	0.994466000
H	−0.283916000	−1.203805000	−3.578387000
H	2.138650000	−1.191807000	−3.132700000
H	3.593698000	−2.718520000	−1.591990000
H	6.005470000	−2.951333000	−1.095861000
H	5.983322000	0.979157000	0.649065000
H	7.206745000	−1.099373000	0.073754000
H	−2.531904000	0.304772000	−2.393013000
H	−2.583539000	−1.428226000	−2.609110000
H	−3.615724000	−4.019940000	0.011092000
H	−2.792493000	−3.398631000	−1.417401000
H	−1.035928000	−2.871090000	0.026239000
H	−3.887611000	1.795080000	−1.695056000
H	−4.845919000	2.165574000	−0.272226000
H	−2.773491000	2.439307000	1.091164000
H	−1.775345000	2.038598000	−0.298002000
H	−2.641871000	4.033308000	−1.526976000
H	−3.674250000	4.439145000	−0.165632000
H	−1.654590000	4.784532000	1.293246000
H	−0.591769000	4.345646000	−0.049053000
H	−1.574428000	5.815081000	−0.141368000

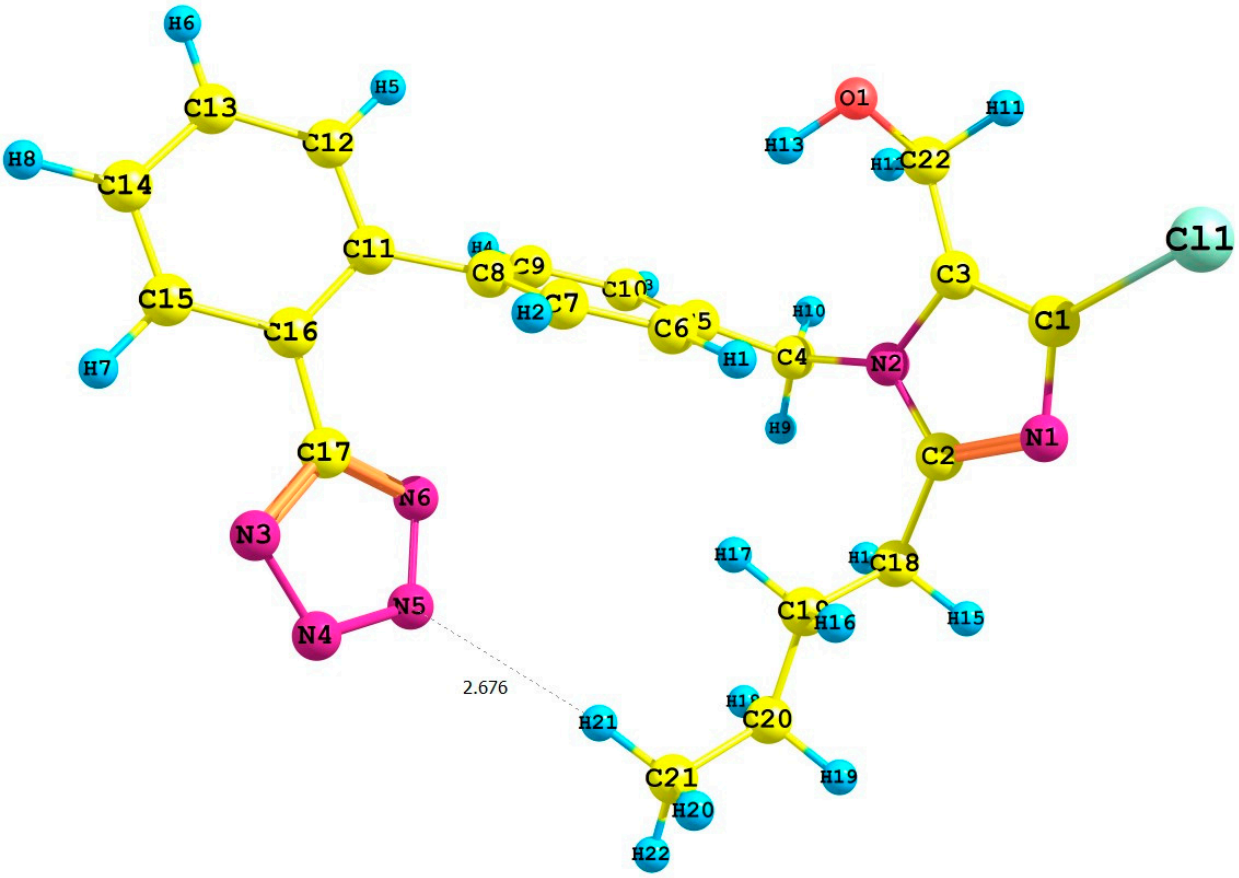

Figure S3. Optimized structure of the *syn*-losartan anion **1** (B3LYP/6-311+G(d,p)/gas level of theory, Rotamer **III**).

Cartesian coordinates:

C	−4.574034000	−1.420936000	0.733870000
N	−4.754792000	−0.078697000	0.705653000
C	−3.931937000	0.365518000	−0.233763000
N	−3.236826000	−0.669550000	−0.807779000
C	−3.645837000	−1.849580000	−0.182440000
C	−2.274436000	−0.581858000	−1.902554000
C	−0.820610000	−0.781659000	−1.511959000
C	−0.356905000	−0.556734000	−0.212494000
C	1.004915000	−0.592223000	0.066989000
C	1.942817000	−0.849572000	−0.937870000
C	1.468800000	−1.134106000	−2.222390000
C	0.106882000	−1.098562000	−2.506694000
C	3.403398000	−0.836346000	−0.643461000
C	4.128422000	−2.022075000	−0.826916000
C	5.482799000	−2.105416000	−0.518481000
C	6.132848000	−0.979414000	−0.012042000
C	5.434171000	0.208703000	0.156868000
C	4.067841000	0.318408000	−0.162831000

C	3.424069000	1.629359000	−0.003615000
N	3.899444000	2.579194000	0.824585000
N	3.074600000	3.615900000	0.661662000
N	2.147381000	3.308613000	−0.237131000
N	2.340783000	2.059799000	−0.674070000
C	−3.780888000	1.811787000	−0.595361000
C	−2.558431000	2.511656000	0.035790000
C	−2.475889000	3.999243000	−0.324819000
C	−1.293243000	4.709883000	0.341667000
C	−3.117882000	−3.207639000	−0.501183000
O	−1.925523000	−3.576662000	0.198007000
Cl	−5.479243000	−2.429789000	1.828507000
H	−1.055225000	−0.318481000	0.581668000
H	1.349691000	−0.376001000	1.070843000
H	−0.232640000	−1.287799000	−3.521524000
H	2.178039000	−1.340608000	−3.016227000
H	3.604556000	−2.899885000	−1.191924000
H	6.018256000	−3.038314000	−0.660347000
H	5.930391000	1.094657000	0.533308000
H	7.186998000	−1.025299000	0.242818000
H	−2.394321000	0.407433000	−2.348855000
H	−2.558652000	−1.305607000	−2.672603000
H	−3.865517000	−3.943605000	−0.203634000
H	−2.967064000	−3.311511000	−1.584465000
H	−1.241680000	−2.919764000	0.011633000
H	−3.746723000	1.931453000	−1.684983000
H	−4.695540000	2.304574000	−0.256480000
H	−2.617418000	2.399588000	1.124271000
H	−1.632712000	2.019101000	−0.276502000
H	−2.393556000	4.098686000	−1.415040000
H	−3.417168000	4.490535000	−0.042446000
H	−1.372028000	4.656401000	1.432804000
H	−0.333655000	4.268018000	0.056289000
H	−1.267647000	5.768634000	0.065131000

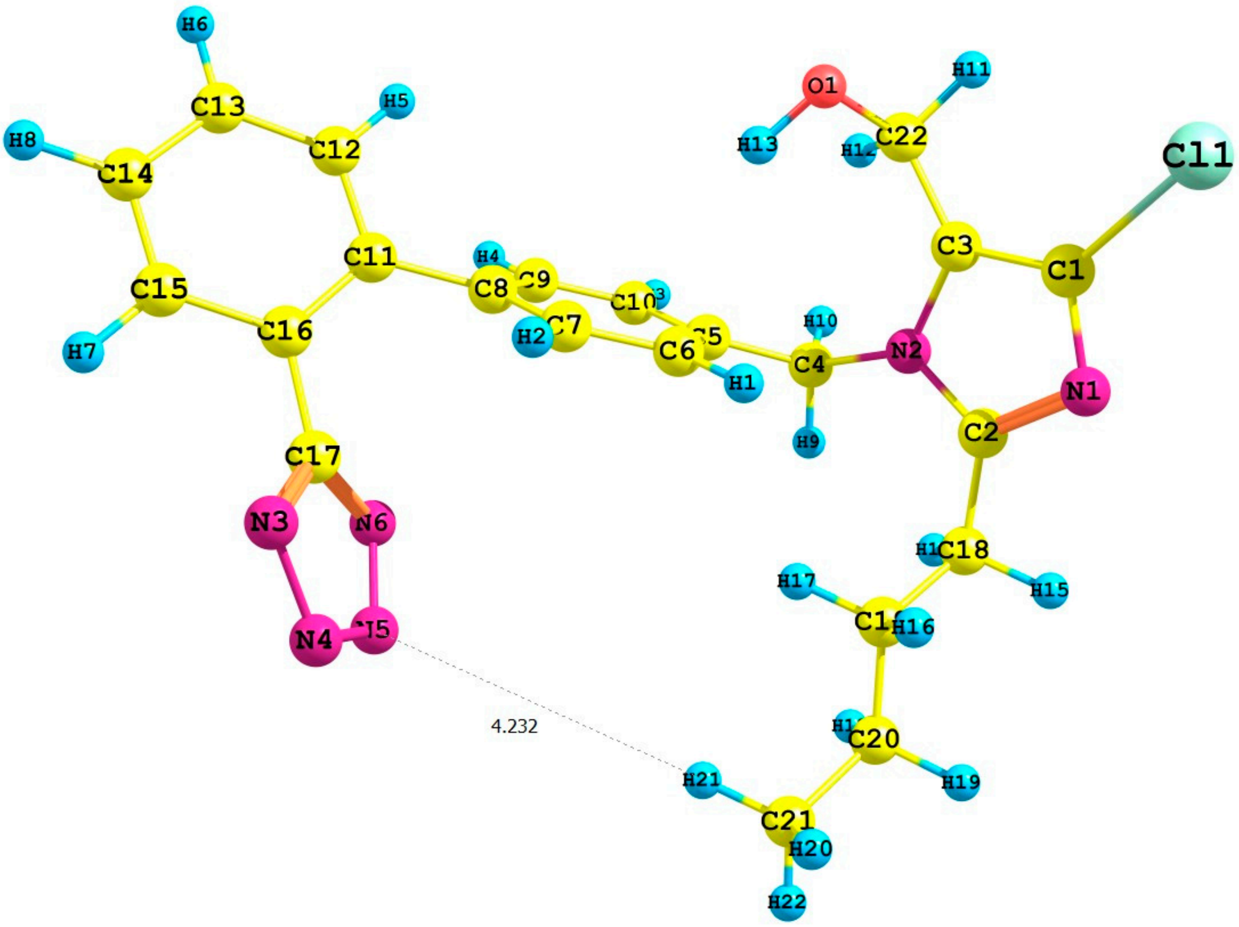

Figure S4. Optimized structure of the *syn*-losartan anion **1** (B3LYP/6-311+G(d,p)/CPCM level of theory, Rotamer **IV**).

Cartesian coordinates:

C	−4.325943000	−1.510529000	0.812589000
N	−4.694331000	−0.205720000	0.776126000
C	−3.959544000	0.338001000	−0.188194000
N	−3.132875000	−0.594009000	−0.757195000
C	−3.365851000	−1.814159000	−0.118529000
C	−2.213276000	−0.387302000	−1.878919000
C	−0.744869000	−0.562642000	−1.531614000
C	−0.231819000	−0.241685000	−0.269815000
C	1.129953000	−0.363204000	−0.003556000
C	2.022741000	−0.805219000	−0.988625000
C	1.501835000	−1.135078000	−2.246513000
C	0.140126000	−1.016087000	−2.513579000
C	3.472179000	−0.984237000	−0.691638000
C	4.052126000	−2.237757000	−0.939831000
C	5.380459000	−2.503817000	−0.620260000
C	6.160258000	−1.507197000	−0.036009000

C	5.606829000	−0.252615000	0.200046000
C	4.273457000	0.035110000	−0.128909000
C	3.786403000	1.411926000	0.096493000
N	3.974279000	2.089503000	1.240971000
N	3.436754000	3.299156000	1.015331000
N	2.957796000	3.346161000	−0.212484000
N	3.162257000	2.167374000	−0.821021000
C	−4.045198000	1.779600000	−0.584956000
C	−2.931823000	2.674189000	−0.003930000
C	−3.115217000	4.149506000	−0.375572000
C	−2.020337000	5.051440000	0.200963000
C	−2.680384000	−3.099309000	−0.438605000
O	−1.488119000	−3.343364000	0.330097000
Cl	−5.057422000	−2.618807000	1.941514000
H	−0.894831000	0.098914000	0.517301000
H	1.499710000	−0.118322000	0.985136000
H	−0.234735000	−1.278944000	−3.497365000
H	2.167849000	−1.482319000	−3.028361000
H	3.437454000	−3.023991000	−1.363577000
H	5.797721000	−3.485075000	−0.815809000
H	6.215860000	0.534462000	0.628731000
H	7.194614000	−1.700675000	0.224634000
H	−2.387599000	0.622143000	−2.253250000
H	−2.487611000	−1.068254000	−2.687514000
H	−3.351955000	−3.922171000	−0.194569000
H	−2.458505000	−3.162484000	−1.508695000
H	−0.848645000	−2.648417000	0.125960000
H	−4.059336000	1.871749000	−1.675772000
H	−5.015050000	2.137330000	−0.231359000
H	−2.922616000	2.567259000	1.086616000
H	−1.953541000	2.331376000	−0.358397000
H	−3.132342000	4.246520000	−1.467708000
H	−4.095376000	4.490252000	−0.021394000
H	−2.000490000	4.998826000	1.294026000
H	−1.031891000	4.755768000	−0.164175000
H	−2.180050000	6.096275000	−0.079355000

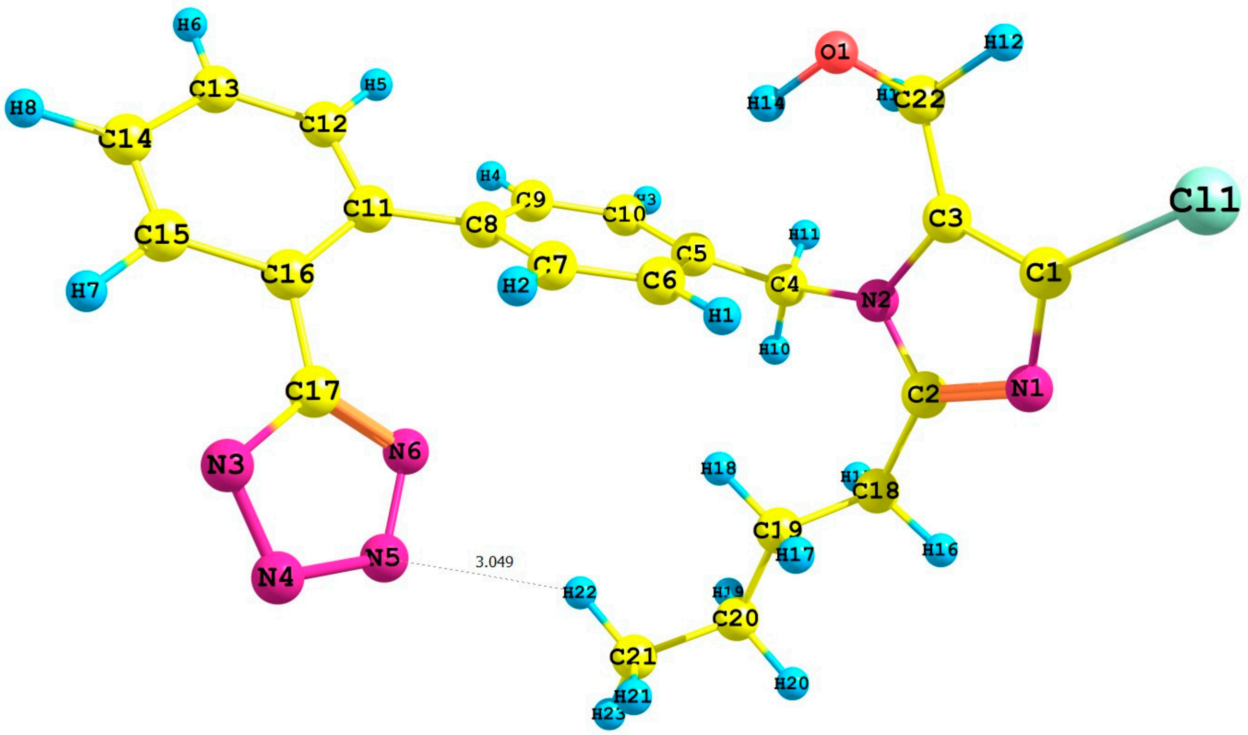

Figure S5. Optimized structure of the *syn*-losartan anion **1** (CAM-B3LYP/6-31G(d,p)/gas level of theory, Rotamer **V**).

Cartesian coordinates:

C	4.602047000	−0.579952000	−0.738177000
N	4.567873000	0.750814000	−0.486537000
C	3.661237000	0.889011000	0.462214000
N	3.127322000	−0.313789000	0.822598000
C	3.729354000	−1.286593000	0.043103000
C	2.124707000	−0.560359000	1.855618000
C	0.754001000	−0.922364000	1.343758000
C	0.253259000	−0.473941000	0.107353000
C	−1.077173000	−0.611100000	−0.224066000
C	−2.025171000	−1.193514000	0.664787000
C	−1.487246000	−1.689947000	1.885641000
C	−0.157672000	−1.552668000	2.213165000
C	−3.422209000	−1.327158000	0.330714000
C	−4.130743000	−2.472604000	0.788389000
C	−5.393409000	−2.796702000	0.364591000
C	−6.056626000	−1.965091000	−0.570162000
C	−5.437621000	−0.813266000	−0.988950000
C	−4.156521000	−0.426237000	−0.532831000
C	−3.751099000	0.925513000	−0.852982000
N	−4.368224000	1.667572000	−1.840006000
N	−3.827413000	2.842442000	−1.843157000

N	−2.916543000	2.821502000	−0.887365000
N	−2.812086000	1.662295000	−0.234198000
C	3.250084000	2.208204000	1.037643000
C	1.866949000	2.681210000	0.571311000
C	1.442363000	4.010901000	1.187782000
C	0.040462000	4.427648000	0.748894000
C	3.389022000	−2.738564000	0.112663000
O	2.195426000	−3.096989000	−0.547746000
Cl	5.683568000	−1.242402000	−1.923023000
H	0.932329000	−0.027744000	−0.614949000
H	−1.406753000	−0.296390000	−1.205914000
H	0.190628000	−1.914192000	3.179839000
H	−2.155014000	−2.150246000	2.607009000
H	−3.609548000	−3.164208000	1.443659000
H	−5.866746000	−3.707926000	0.720936000
H	−5.951141000	−0.136735000	−1.664299000
H	−7.046369000	−2.221420000	−0.936067000
H	2.094308000	0.350488000	2.465773000
H	2.505217000	−1.347738000	2.517016000
H	4.193505000	−3.290893000	−0.378432000
H	3.374018000	−3.061253000	1.165939000
H	1.468296000	−2.599556000	−0.139165000
H	3.273053000	2.171418000	2.134079000
H	4.015141000	2.925570000	0.729548000
H	1.876103000	2.765888000	−0.521792000
H	1.112794000	1.924350000	0.807475000
H	1.469135000	3.927537000	2.281821000
H	2.165042000	4.793289000	0.922754000
H	0.002085000	4.565147000	−0.337732000
H	−0.688860000	3.656619000	1.019039000

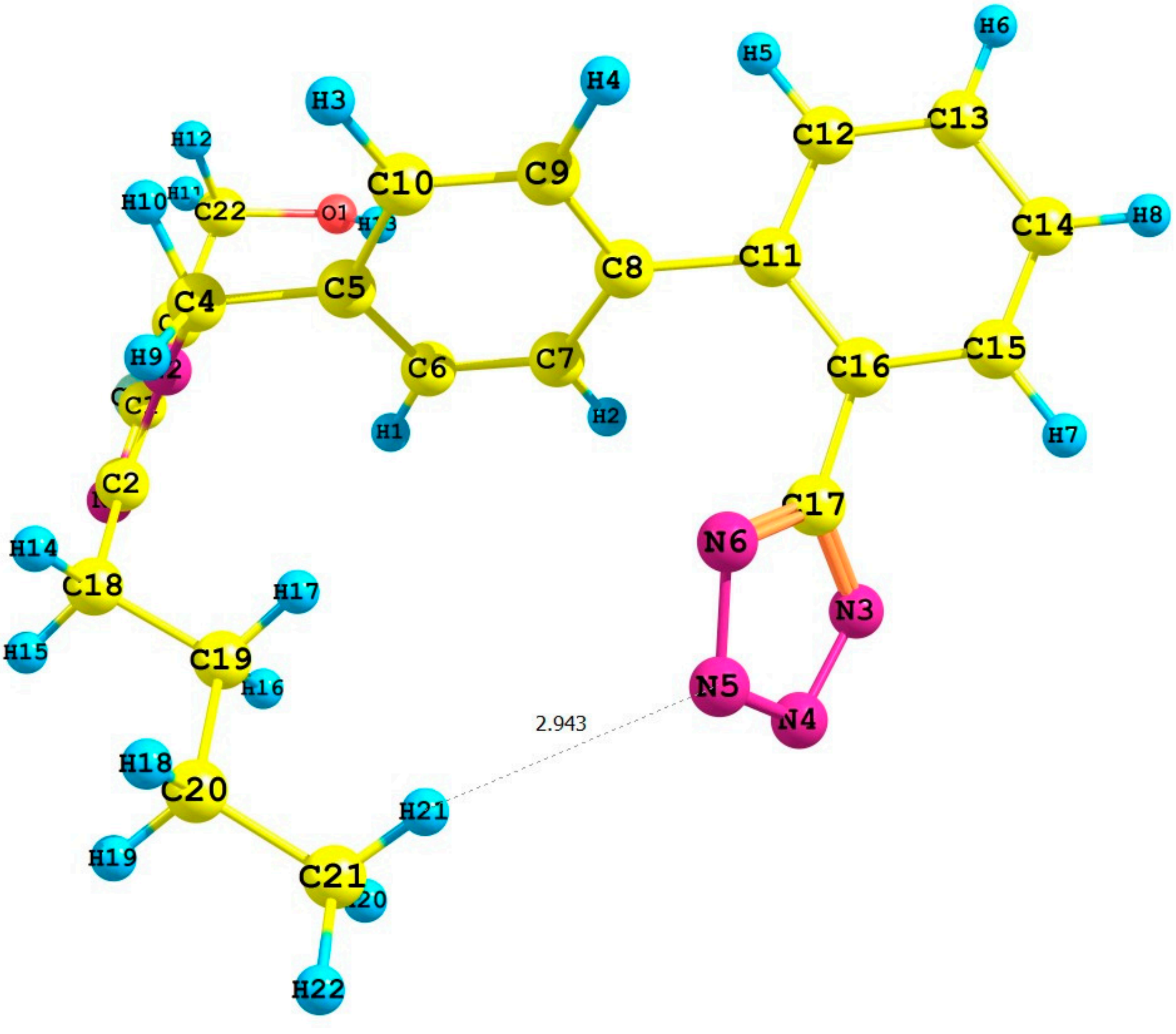

Figure S6. Optimized structure of the *syn*-losartan anion **1** (CAM-B3LYP/6-31G(d,p)/CPCM level of theory, Rotamer **VI**).

Cartesian coordinates:

C	1.008001000	2.105147000	0.308414000
N	1.231773000	2.134323000	1.644925000
C	2.064393000	1.134064000	1.872642000
N	2.372004000	0.476181000	0.716748000
C	1.691367000	1.102028000	−0.318498000
C	3.272756000	−0.655867000	0.565643000
C	2.582249000	−1.979501000	0.305992000
C	1.239045000	−2.195719000	0.616339000
C	0.668109000	−3.451044000	0.446282000
C	1.421119000	−4.524159000	−0.030919000
C	2.755606000	−4.293415000	−0.371622000
C	3.328414000	−3.039709000	−0.206394000
C	0.807005000	−5.866339000	−0.221416000

C	0.867187000	−6.448846000	−1.490876000
C	0.257913000	−7.666960000	−1.760658000
C	−0.432614000	−8.325188000	−0.749623000
C	−0.483603000	−7.769177000	0.520745000
C	0.137143000	−6.550047000	0.811649000
C	0.082614000	−6.062981000	2.204280000
N	−1.001812000	−6.182032000	2.980572000
N	−0.629487000	−5.679359000	4.160463000
N	0.623506000	−5.285458000	4.094712000
N	1.104506000	−5.514361000	2.870223000
C	2.564240000	0.759024000	3.231409000
C	1.790353000	−0.400749000	3.874633000
C	2.293814000	−0.741547000	5.274605000
C	1.473826000	−1.841631000	5.942714000
C	1.721445000	0.667463000	−1.745383000
O	0.755413000	−0.324704000	−2.067568000
Cl	−0.037710000	3.262564000	−0.449932000
H	0.632356000	−1.381948000	0.999504000
H	−0.375694000	−3.601641000	0.699724000
H	4.372057000	−2.887994000	−0.466504000
H	3.357044000	−5.110649000	−0.756661000
H	1.381892000	−5.916972000	−2.284977000
H	0.313930000	−8.092606000	−2.757142000
H	−0.999544000	−8.285670000	1.322587000
H	−0.920538000	−9.274427000	−0.945779000
H	3.864102000	−0.724726000	1.480367000
H	3.979139000	−0.435816000	−0.238533000
H	1.483879000	1.532145000	−2.367644000
H	2.731029000	0.339640000	−2.022372000
H	0.985909000	−1.131641000	−1.587355000
H	3.632458000	0.519895000	3.196442000
H	2.465091000	1.651012000	3.854889000
H	0.728185000	−0.132142000	3.919163000
H	1.856706000	−1.292258000	3.240771000
H	3.345583000	−1.046963000	5.214122000
H	2.273122000	0.161968000	5.896299000
H	0.432489000	−1.528419000	6.069994000
H	1.469660000	−2.760805000	5.348204000
H	1.869504000	−2.084735000	6.932781000

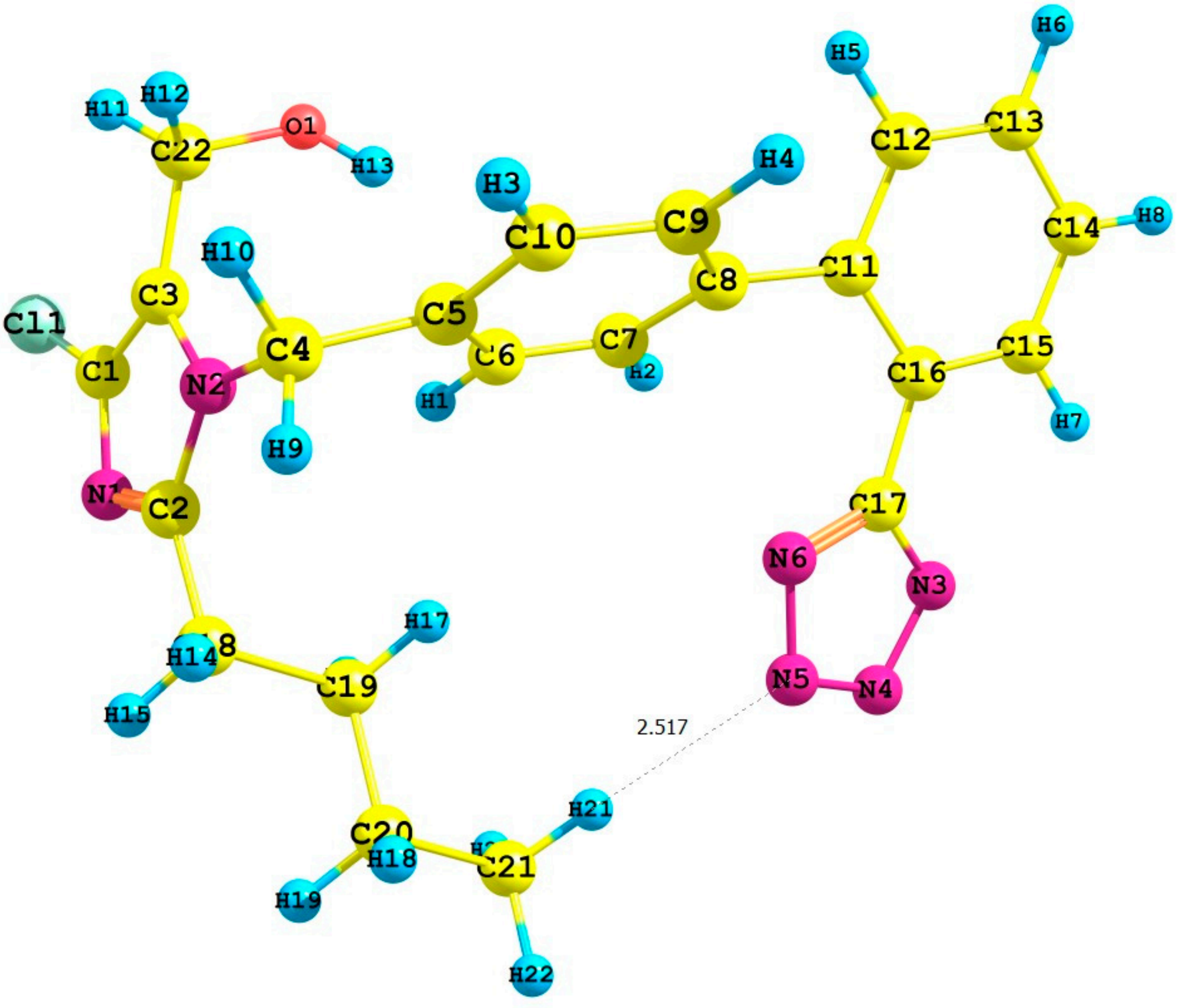

Figure S7. Optimized structure of the *syn*-losartan anion **1** (PBE1PBE/6-31G(d,p)/gas level of theory, Rotamer **VII**).

Cartesian coordinates:

C	4.609937000	−0.571766000	−0.707229000
N	4.526050000	0.770088000	−0.524554000
C	3.576633000	0.933777000	0.387985000
N	3.064057000	−0.276316000	0.792681000
C	3.730749000	−1.275573000	0.081905000
C	2.044480000	−0.504073000	1.811212000
C	0.667466000	−0.873726000	1.286522000
C	0.266215000	−0.590866000	−0.026646000
C	−1.056057000	−0.781062000	−0.417210000
C	−2.016851000	−1.258991000	0.483860000
C	−1.592633000	−1.603108000	1.774269000
C	−0.270665000	−1.411868000	2.172540000
C	−3.440980000	−1.432179000	0.079238000
C	−3.988230000	−2.723169000	0.159786000

C	−5.295973000	−2.994022000	−0.234135000
C	−6.083001000	−1.948515000	−0.723480000
C	−5.565614000	−0.660906000	−0.792837000
C	−4.247671000	−0.360677000	−0.388710000
C	−3.821968000	1.044156000	−0.450411000
N	−4.537373000	1.985243000	−1.105014000
N	−3.864904000	3.121376000	−0.910195000
N	−2.789441000	2.876848000	−0.159409000
N	−2.736398000	1.576121000	0.145973000
C	3.111712000	2.275312000	0.872999000
C	1.850932000	2.816445000	0.161877000
C	1.455067000	4.217289000	0.645177000
C	0.259983000	4.798825000	−0.118282000
C	3.433626000	−2.736091000	0.193523000
O	2.347093000	−3.192766000	−0.606437000
Cl	5.760790000	−1.260509000	−1.823349000
H	0.978974000	−0.181937000	−0.735969000
H	−1.359525000	−0.517370000	−1.424039000
H	0.024099000	−1.652312000	3.192432000
H	−2.318884000	−1.988173000	2.484268000
H	−3.354021000	−3.530568000	0.518187000
H	−5.689510000	−4.005095000	−0.168764000
H	−6.168806000	0.165000000	−1.153848000
H	−7.105496000	−2.136021000	−1.043291000
H	1.971544000	0.417385000	2.396015000
H	2.408879000	−1.275441000	2.499611000
H	4.311513000	−3.284425000	−0.157663000
H	3.285372000	−3.009223000	1.251324000
H	1.560389000	−2.683047000	−0.358583000
H	2.940640000	2.255760000	1.957579000
H	3.945315000	2.963730000	0.701789000
H	2.047877000	2.840184000	−0.917534000
H	1.005368000	2.134913000	0.308102000
H	1.218715000	4.172143000	1.718002000
H	2.323373000	4.887434000	0.553145000
H	0.491954000	4.891954000	−1.186349000
H	−0.638400000	4.176265000	−0.025564000
H	0.013558000	5.800839000	0.251490000

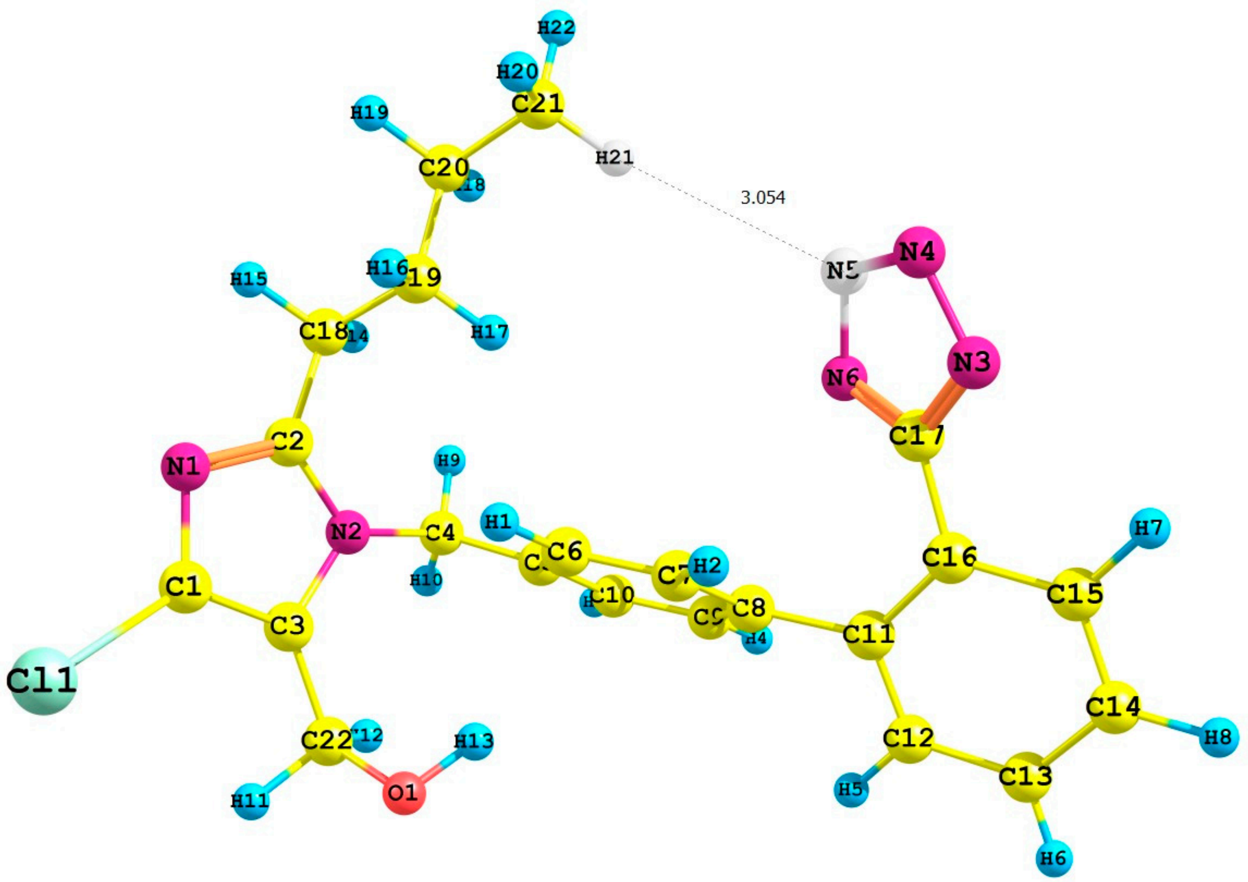

Figure S8. Optimized structure of the *syn*-losartan anion **1** (PBE1PBE/6-31G(d,p)/CPCM level of theory, Rotamer **VIII**).

Cartesian coordinates:

C	0.181920000	0.055362000	−0.112714000
N	0.040662000	0.112880000	1.232423000
C	1.277047000	0.061336000	1.704804000
N	2.187833000	−0.024094000	0.692201000
C	1.492830000	−0.026907000	−0.507061000
C	3.632256000	−0.072531000	0.816095000
C	4.240220000	−1.416688000	0.483948000
C	3.509664000	−2.605629000	0.581374000
C	4.126592000	−3.833591000	0.368859000
C	5.487720000	−3.910897000	0.056589000
C	6.203188000	−2.716367000	−0.079856000
C	5.589313000	−1.486711000	0.131601000
C	6.144317000	−5.221661000	−0.173844000
C	6.805000000	−5.429717000	−1.391068000
C	7.376676000	−6.656946000	−1.707988000
C	7.289235000	−7.708199000	−0.798788000
C	6.657632000	−7.510421000	0.423057000
C	6.090589000	−6.274932000	0.765175000

C	5.503501000	−6.133596000	2.107560000
N	4.831171000	−7.119375000	2.721476000
N	4.521009000	−6.619900000	3.917439000
N	4.993782000	−5.394992000	4.021370000
N	5.620792000	−5.055288000	2.895739000
C	1.612162000	0.055400000	3.159464000
C	1.900453000	−1.342785000	3.721691000
C	2.188227000	−1.330010000	5.219035000
C	2.410578000	−2.727085000	5.785821000
C	2.117367000	−0.157570000	−1.853786000
O	2.293165000	−1.497800000	−2.283781000
Cl	−1.193080000	0.100405000	−1.157734000
H	2.452474000	−2.574296000	0.831011000
H	3.546107000	−4.747092000	0.455918000
H	6.169718000	−0.572415000	0.035157000
H	7.258526000	−2.752316000	−0.335757000
H	6.840410000	−4.615944000	−2.110632000
H	7.875792000	−6.793202000	−2.662871000
H	6.607746000	−8.314823000	1.150669000
H	7.723134000	−8.675757000	−1.034303000
H	3.879082000	0.197647000	1.846601000
H	4.068477000	0.706732000	0.183111000
H	1.449351000	0.308698000	−2.582647000
H	3.064287000	0.399254000	−1.886473000
H	2.927662000	−1.913230000	−1.685246000
H	2.459538000	0.721300000	3.364279000
H	0.748675000	0.484594000	3.676784000
H	1.037497000	−1.989664000	3.516652000
H	2.752673000	−1.790624000	3.194550000
H	3.070387000	−0.705035000	5.411585000
H	1.352533000	−0.847925000	5.743671000
H	1.518568000	−3.349349000	5.652162000
H	3.243795000	−3.237209000	5.289217000
H	2.631756000	−2.690884000	6.857142000

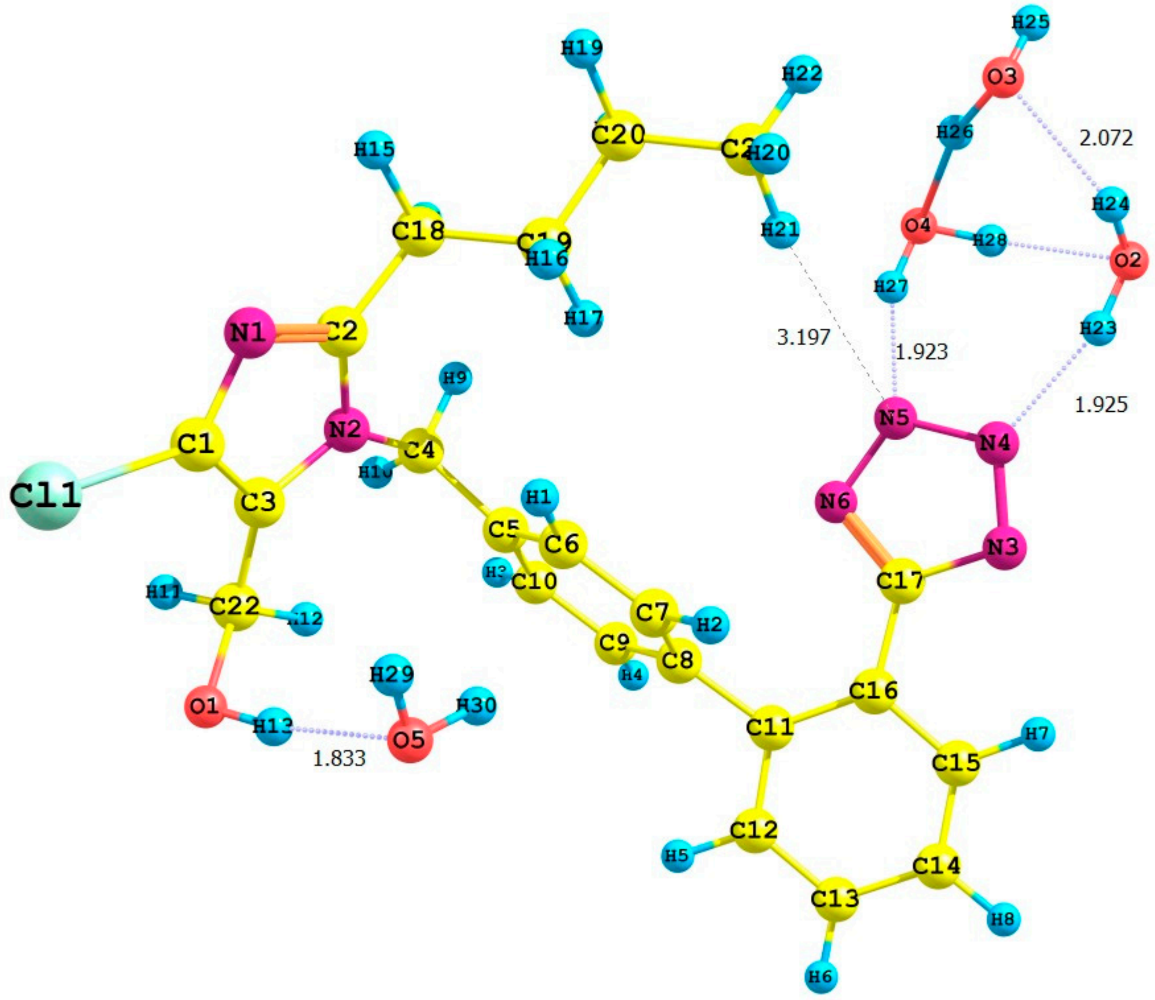

Figure S9. Optimized structure of the *syn*-losartan anion cluster **1** with water molecules (**IX**); interaction of **1** with three water molecules (B3LYP/6-31G(d,p) level of theory, gas).

Cartesian coordinates:

C	−4.703337000	−2.175466000	0.125652000
N	−5.007321000	−0.879210000	0.405212000
C	−4.221845000	−0.160575000	−0.382799000
N	−3.416841000	−0.972915000	−1.151518000
C	−3.721215000	−2.297589000	−0.831649000
C	−2.410224000	−0.540348000	−2.119200000
C	−0.969661000	−0.712566000	−1.668853000
C	−0.590629000	−0.523317000	−0.331925000
C	0.752420000	−0.528598000	0.034216000
C	1.761060000	−0.729105000	−0.921379000
C	1.374321000	−0.976929000	−2.242336000
C	0.028166000	−0.963277000	−2.612335000
C	3.198722000	−0.713090000	−0.525539000
C	3.928116000	−1.905884000	−0.647478000
C	5.257461000	−1.994670000	−0.241685000

C	5.878237000	−0.868216000	0.301707000
C	5.177676000	0.327748000	0.409597000
C	3.837394000	0.441335000	−0.007111000
C	3.203770000	1.766669000	0.080897000
N	3.725954000	2.770614000	0.817000000
N	2.920677000	3.802388000	0.589602000
N	1.953266000	3.441329000	−0.253006000
N	2.106009000	2.163030000	−0.592476000
C	−4.228587000	1.341532000	−0.414431000
C	−3.083896000	2.017598000	0.368464000
C	−3.172657000	3.547938000	0.397048000
C	−2.014981000	4.178344000	1.177490000
C	−3.119034000	−3.496550000	−1.522802000
O	−2.778614000	−4.555972000	−0.659137000
Cl	−5.549030000	−3.475390000	0.919838000
H	−1.349692000	−0.331660000	0.419825000
H	1.031590000	−0.333649000	1.064833000
H	−0.242715000	−1.125851000	−3.653412000
H	2.138411000	−1.146085000	−2.995129000
H	3.424215000	−2.782463000	−1.046673000
H	5.796489000	−2.933048000	−0.339721000
H	5.652478000	1.216955000	0.809633000
H	6.912896000	−0.918139000	0.631359000
H	−2.603345000	0.517423000	−2.320504000
H	−2.573481000	−1.069316000	−3.063810000
H	−3.849229000	−3.909962000	−2.231246000
H	−2.256189000	−3.162925000	−2.120781000
H	−2.113793000	−4.220218000	−0.022800000
H	−4.225696000	1.703422000	−1.451640000
H	−5.188945000	1.640895000	0.016418000
H	−3.088966000	1.630757000	1.396152000
H	−2.117086000	1.736901000	−0.063382000
H	−3.172277000	3.928280000	−0.634042000
H	−4.135019000	3.850495000	0.834614000
H	−2.028808000	3.854797000	2.225234000
H	−1.047437000	3.885206000	0.756176000
H	−2.048711000	5.270672000	1.167281000
O	2.815847000	6.666489000	0.861358000
H	2.989576000	5.698086000	0.915514000
H	1.956421000	6.768219000	1.308472000
O	−0.099839000	6.992744000	1.189685000
H	−0.132831000	7.920454000	0.922871000

H	0.103756000	6.528610000	0.335551000
O	0.943614000	5.971280000	−1.106913000
H	1.096054000	5.008160000	−0.964915000
H	1.760639000	6.343915000	−0.710728000
O	−1.061928000	−3.358390000	1.205634000
H	−1.755992000	−2.879046000	1.678060000
H	−0.556331000	−2.661934000	0.756264000

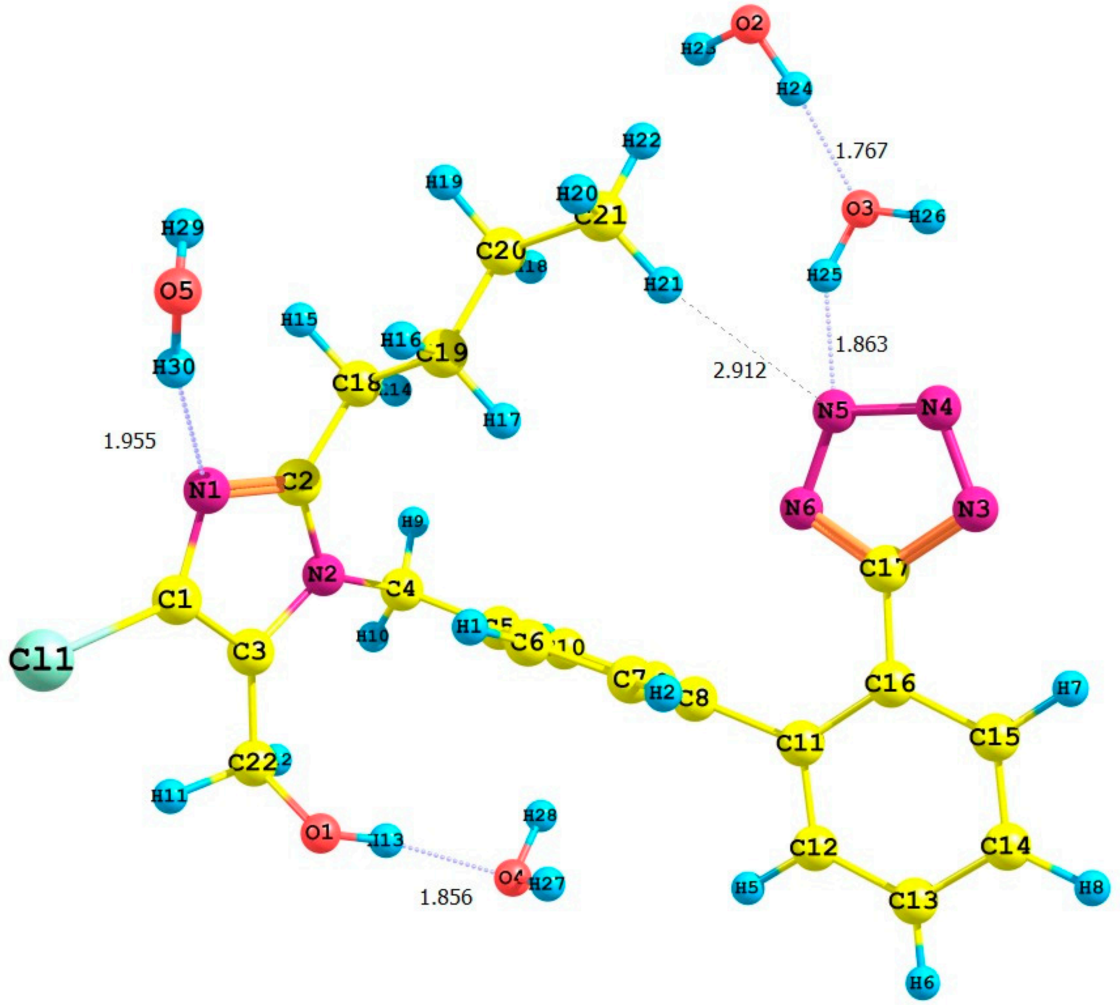

Figure S10. Optimized structure of the *syn*-losartan anion cluster **1** with water molecules (**X**); interaction of **1** with three water molecules (PBE1PBE/6-31G(d,p) level of theory, gas).

Cartesian coordinates:

C	−2.306827000	−1.280895000	−1.927300000
N	−2.806570000	−0.380853000	−1.046412000
C	−2.528295000	0.805461000	−1.561722000
N	−1.871590000	0.673886000	−2.751087000
C	−1.712678000	−0.681585000	−3.007344000
C	−1.433028000	1.743821000	−3.619838000
C	0.002312000	2.177461000	−3.430694000

C	0.805317000	1.684746000	−2.404315000
C	2.108490000	2.145932000	−2.248968000
C	2.647091000	3.095962000	−3.119892000
C	1.845556000	3.567905000	−4.164511000
C	0.535978000	3.120844000	−4.312204000
C	4.064223000	3.527787000	−2.993378000
C	4.980248000	3.008180000	−3.914622000
C	6.334353000	3.311176000	−3.841023000
C	6.784419000	4.150971000	−2.824210000
C	5.885356000	4.686182000	−1.914119000
C	4.512249000	4.398391000	−1.978433000
C	3.623918000	5.044673000	−1.012268000
N	4.085843000	5.735884000	0.045013000
N	3.002255000	6.200827000	0.638012000
N	1.931157000	5.809557000	−0.035205000
N	2.285533000	5.081085000	−1.081278000
C	−2.850502000	2.097043000	−0.886572000
C	−1.832877000	2.494152000	0.193339000
C	−2.185157000	3.811992000	0.875009000
C	−1.137526000	4.242184000	1.894364000
C	−0.982133000	−1.274789000	−4.168314000
O	0.403742000	−1.390126000	−3.967316000
Cl	−2.462876000	−2.974334000	−1.656413000
H	0.416866000	0.928786000	−1.729120000
H	2.718027000	1.773802000	−1.431097000
H	−0.078546000	3.516540000	−5.119039000
H	2.248997000	4.308898000	−4.848249000
H	4.612019000	2.338965000	−4.690153000
H	7.028494000	2.891205000	−4.563761000
H	6.214700000	5.353628000	−1.124030000
H	7.840549000	4.396206000	−2.745134000
H	−2.098242000	2.597310000	−3.457558000
H	−1.597192000	1.425077000	−4.655363000
H	−1.391985000	−2.282577000	−4.307342000
H	−1.218114000	−0.716760000	−5.087480000
H	0.880509000	−0.696380000	−4.457983000
H	−2.942856000	2.906048000	−1.620669000
H	−3.839659000	1.983169000	−0.428003000
H	−1.792350000	1.686503000	0.934833000
H	−0.836099000	2.580747000	−0.255378000
H	−2.294396000	4.596383000	0.112297000
H	−3.168460000	3.717663000	1.359525000

H	−1.009181000	3.474387000	2.666156000
H	−0.167751000	4.400224000	1.409895000
H	−1.411901000	5.180088000	2.388420000
O	−1.677242000	7.495227000	2.994932000
H	−2.323748000	7.508340000	2.284326000
H	−0.828667000	7.545941000	2.500590000
O	0.536564000	7.692188000	1.387761000
H	0.694777000	6.960682000	0.750597000
H	1.404623000	7.722060000	1.806982000
O	2.214160000	0.394223000	−5.149489000
H	2.762183000	0.319919000	−4.359858000
H	1.884382000	1.302476000	−5.086224000
O	−3.167550000	−0.309747000	1.840474000
H	−3.879065000	0.334354000	1.881031000
H	−3.081132000	−0.487935000	0.886074000

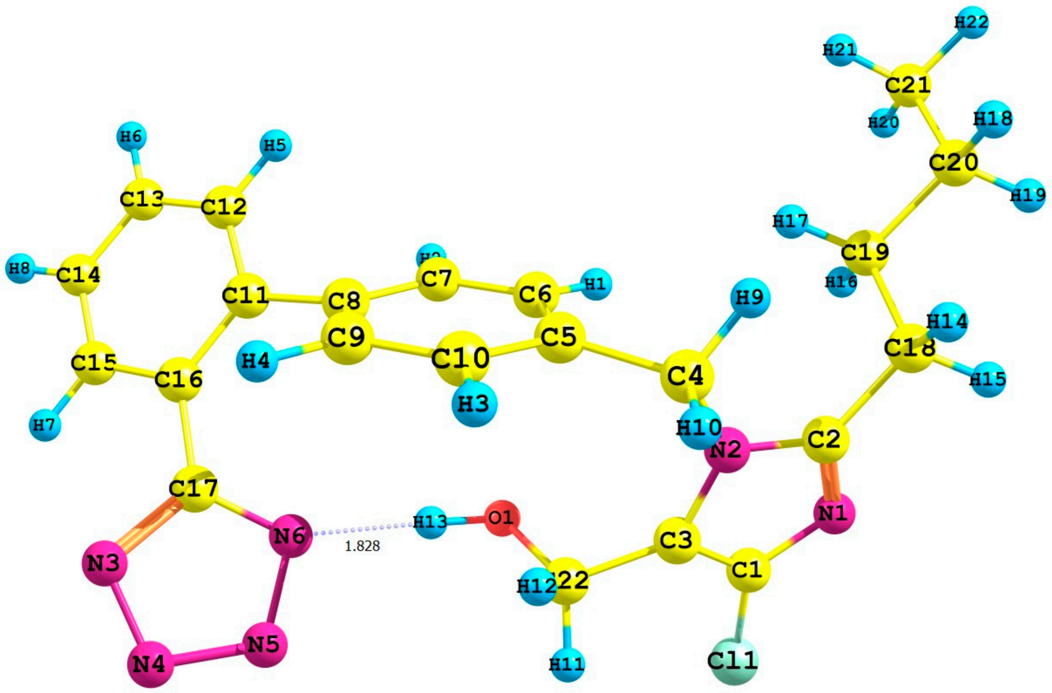

Figure S11. Optimized structure of the *anti*-losartan anion **1** (B3LYP/6-31G(d,p)/gas level of theory, Rotamer **XI**).

Cartesian coordinates:

C	−3.643676000	−1.681373000	0.592262000
N	−4.375550000	−0.534480000	0.594284000
C	−3.790541000	0.234100000	−0.310078000
N	−2.703100000	−0.388323000	−0.878465000
C	−2.598293000	−1.659045000	−0.301727000
C	−1.937678000	0.130864000	−2.022860000

C	−0.437784000	0.012749000	−1.875963000
C	0.227771000	0.519356000	−0.757629000
C	1.578746000	0.248510000	−0.561099000
C	2.291969000	−0.546148000	−1.468989000
C	1.644080000	−0.962934000	−2.639712000
C	0.297351000	−0.685822000	−2.838379000
C	3.690886000	−0.963964000	−1.184144000
C	4.651098000	0.027166000	−0.925925000
C	5.988730000	−0.288530000	−0.701664000
C	6.384989000	−1.627019000	−0.738049000
C	5.443352000	−2.623664000	−0.970355000
C	4.082414000	−2.327905000	−1.182682000
C	3.170443000	−3.470731000	−1.380706000
N	3.605432000	−4.632500000	−1.903798000
N	2.534835000	−5.439013000	−1.896419000
N	1.497278000	−4.799046000	−1.380186000
N	1.864744000	−3.551751000	−1.045288000
C	−4.259030000	1.625540000	−0.621220000
C	−3.426881000	2.739453000	0.046333000
C	−3.983122000	4.143076000	−0.219758000
C	−3.166662000	5.251845000	0.451179000
C	−1.518994000	−2.674133000	−0.561921000
O	−0.375262000	−2.370757000	0.199493000
Cl	−4.068321000	−3.003288000	1.649597000
H	−0.329334000	1.053818000	0.007181000
H	2.068812000	0.583551000	0.348396000
H	−0.204131000	−1.071186000	−3.723448000
H	2.186169000	−1.562022000	−3.363376000
H	4.334010000	1.067140000	−0.931907000
H	6.712175000	0.500301000	−0.511847000
H	5.732535000	−3.668742000	−0.988486000
H	7.425805000	−1.895678000	−0.573907000
H	−2.239716000	1.174682000	−2.143555000
H	−2.254632000	−0.399086000	−2.929182000
H	−1.941946000	−3.656332000	−0.297371000
H	−1.298333000	−2.715564000	−1.638676000
H	0.424422000	−2.768259000	−0.237420000
H	−4.302199000	1.798466000	−1.705091000
H	−5.289126000	1.681018000	−0.255820000
H	−3.396638000	2.549999000	1.126502000
H	−2.386334000	2.688805000	−0.301509000
H	−4.019940000	4.320708000	−1.303794000

H	−5.023597000	4.190743000	0.129943000
H	−3.138424000	5.118219000	1.538441000
H	−2.130703000	5.252334000	0.093708000
H	−3.589374000	6.241746000	0.247849000

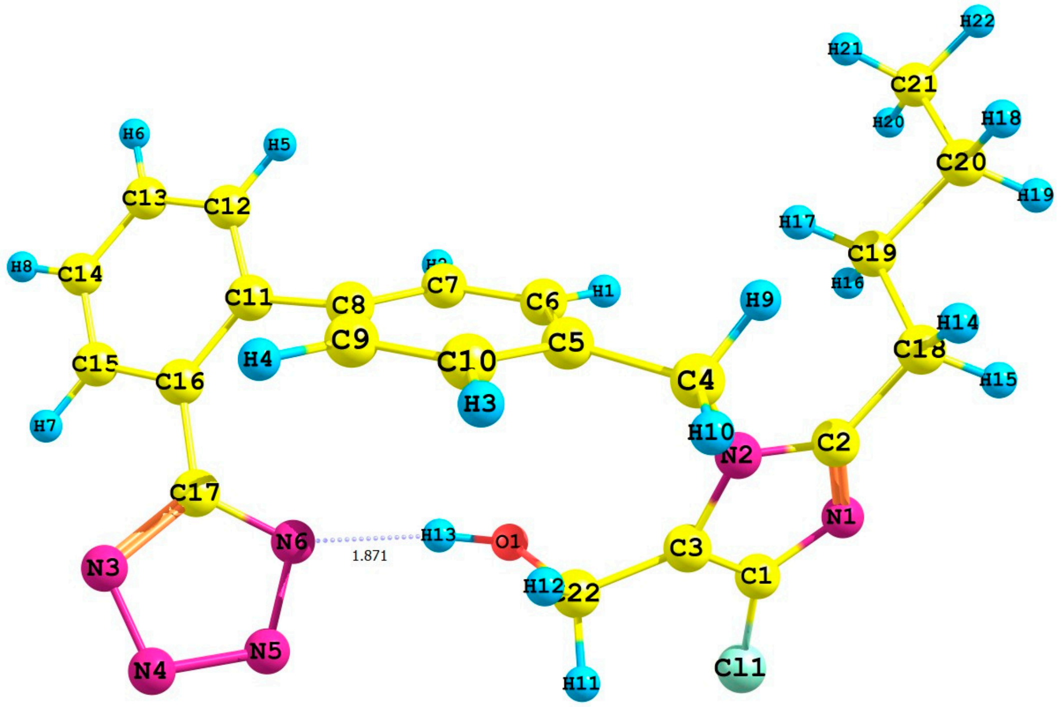

Figure S12. Optimized structure of the *anti*-losartan anion **1** (B3LYP/6-31G(d,p)/CPCM level of theory, Rotamer **XII**).

Cartesian coordinates

C	−3.636647000	−1.682024000	0.592163000
N	−4.346613000	−0.524124000	0.626796000
C	−3.780618000	0.243709000	−0.298001000
N	−2.726943000	−0.396306000	−0.902997000
C	−2.625574000	−1.670894000	−0.338038000
C	−1.962126000	0.117855000	−2.053415000
C	−0.463385000	−0.017812000	−1.890501000
C	0.192021000	0.468558000	−0.755296000
C	1.545218000	0.206707000	−0.555291000
C	2.271568000	−0.558908000	−1.479074000
C	1.633767000	−0.959640000	−2.661137000
C	0.283351000	−0.691808000	−2.863015000
C	3.679313000	−0.950977000	−1.195682000
C	4.626106000	0.058002000	−0.956354000
C	5.967941000	−0.237236000	−0.724301000
C	6.386287000	−1.568886000	−0.728214000
C	5.457062000	−2.583309000	−0.942317000

C	4.096079000	−2.305593000	−1.168430000
C	3.191592000	−3.464966000	−1.342514000
N	3.608644000	−4.611993000	−1.911075000
N	2.556407000	−5.443318000	−1.837322000
N	1.547893000	−4.830734000	−1.243596000
N	1.915377000	−3.578598000	−0.918635000
C	−4.240625000	1.639357000	−0.600549000
C	−3.389473000	2.744433000	0.059583000
C	−3.942441000	4.150039000	−0.202700000
C	−3.108603000	5.252991000	0.455792000
C	−1.588640000	−2.712668000	−0.635962000
O	−0.484573000	−2.586425000	0.250513000
Cl	−4.037269000	−3.007025000	1.661460000
H	−0.367206000	1.007159000	0.004608000
H	2.030964000	0.547405000	0.354495000
H	−0.206208000	−1.047607000	−3.766020000
H	2.189056000	−1.520817000	−3.406044000
H	4.298063000	1.093311000	−0.979268000
H	6.679029000	0.564815000	−0.550361000
H	5.771529000	−3.621215000	−0.925147000
H	7.428715000	−1.818440000	−0.553434000
H	−2.248459000	1.163986000	−2.175714000
H	−2.285799000	−0.410451000	−2.955668000
H	−2.074209000	−3.693036000	−0.514569000
H	−1.276359000	−2.644025000	−1.683217000
H	0.347854000	−2.834223000	−0.219250000
H	−4.290855000	1.806798000	−1.683009000
H	−5.267396000	1.708965000	−0.228871000
H	−3.346439000	2.558816000	1.140346000
H	−2.354952000	2.688166000	−0.304175000
H	−3.991079000	4.323005000	−1.286148000
H	−4.976934000	4.203452000	0.162026000
H	−3.067629000	5.122162000	1.543028000
H	−2.078293000	5.246950000	0.082414000
H	−3.529763000	6.243315000	0.254849000

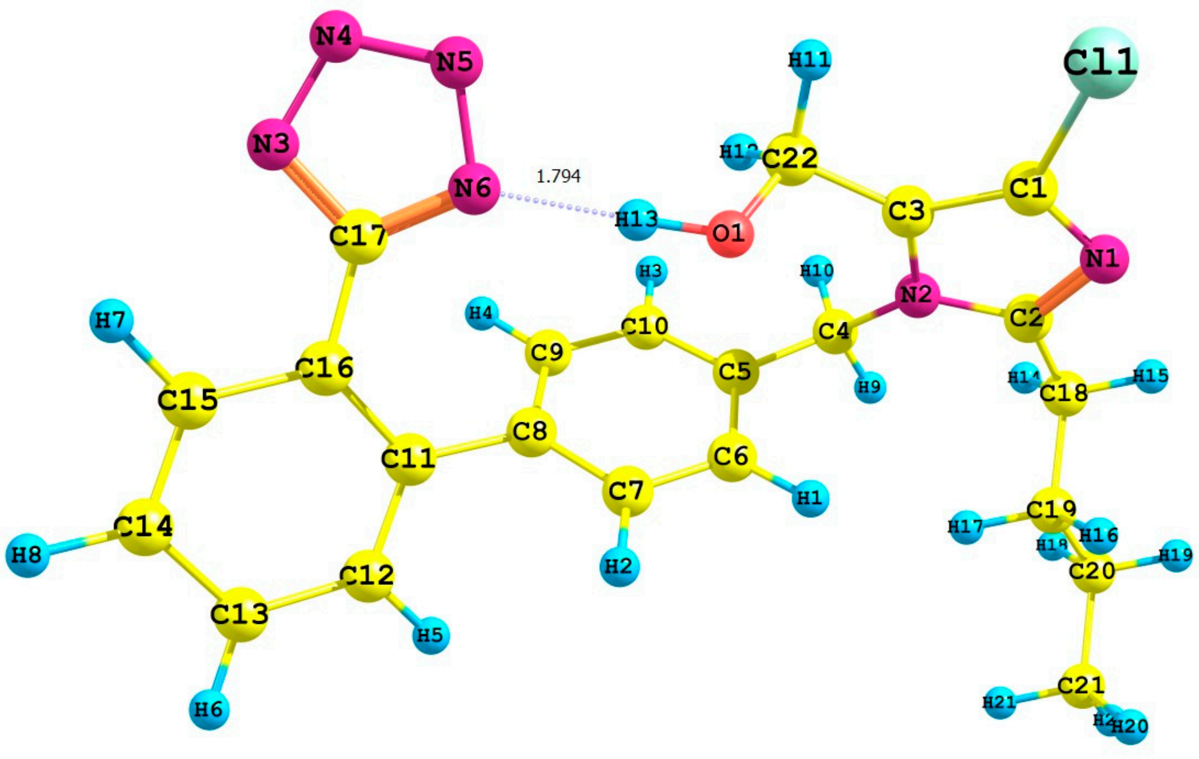

Figure S13. Optimized structure of the *anti*-losartan anion **1** (PBE1PBE/6-31G(d,p)/gas level of theory, Rotamer **XIII**).

Cartesian coordinates:

C	0.903040000	−0.303934000	−0.391257000
N	0.453805000	0.099051000	0.822609000
C	1.517227000	0.043815000	1.600283000
N	2.620674000	−0.388920000	0.918456000
C	2.240290000	−0.614921000	−0.398865000
C	3.978649000	−0.420376000	1.449686000
C	4.720753000	−1.693419000	1.144650000
C	4.178178000	−2.938286000	1.453660000
C	4.793771000	−4.100130000	1.007938000
C	5.952889000	−4.041398000	0.229835000
C	6.543375000	−2.794786000	0.004944000
C	5.933523000	−1.636739000	0.459302000
C	6.526399000	−5.265860000	−0.374078000
C	6.830357000	−6.349888000	0.457653000
C	7.416703000	−7.507572000	−0.037922000
C	7.712855000	−7.588415000	−1.396719000
C	7.397608000	−6.531298000	−2.238211000
C	6.788728000	−5.360137000	−1.759679000
C	6.465540000	−4.323041000	−2.750183000
N	7.169475000	−4.196574000	−3.883647000
N	6.581167000	−3.202939000	−4.543817000

N	5.558930000	−2.751419000	−3.851567000
N	5.457238000	−3.434676000	−2.712988000
C	1.481267000	0.374226000	3.056807000
C	1.454685000	−0.853504000	3.974528000
C	1.336516000	−0.489109000	5.450511000
C	1.302780000	−1.708949000	6.362582000
C	3.105485000	−1.162491000	−1.492916000
O	3.151469000	−2.554985000	−1.418689000
Cl	−0.168105000	−0.383488000	−1.743860000
H	3.222643000	−2.998389000	1.968773000
H	4.325570000	−5.063959000	1.187658000
H	6.372391000	−0.669932000	0.221224000
H	7.451088000	−2.735735000	−0.586905000
H	6.622675000	−6.257531000	1.521236000
H	7.649816000	−8.331783000	0.631278000
H	7.607954000	−6.579578000	−3.301892000
H	8.180215000	−8.481666000	−1.804001000
H	3.893088000	−0.260783000	2.528719000
H	4.538850000	0.431472000	1.044739000
H	2.669214000	−0.809184000	−2.441763000
H	4.107062000	−0.709229000	−1.430101000
H	3.980943000	−2.879861000	−1.858051000
H	2.317827000	1.028690000	3.338108000
H	0.566337000	0.955728000	3.206998000
H	0.611059000	−1.489611000	3.678647000
H	2.358988000	−1.457725000	3.819950000
H	2.177526000	0.159068000	5.733564000
H	0.428790000	0.110770000	5.601646000
H	0.453333000	−2.357065000	6.120945000
H	2.213378000	−2.308052000	6.253140000
H	1.215877000	−1.423884000	7.416107000

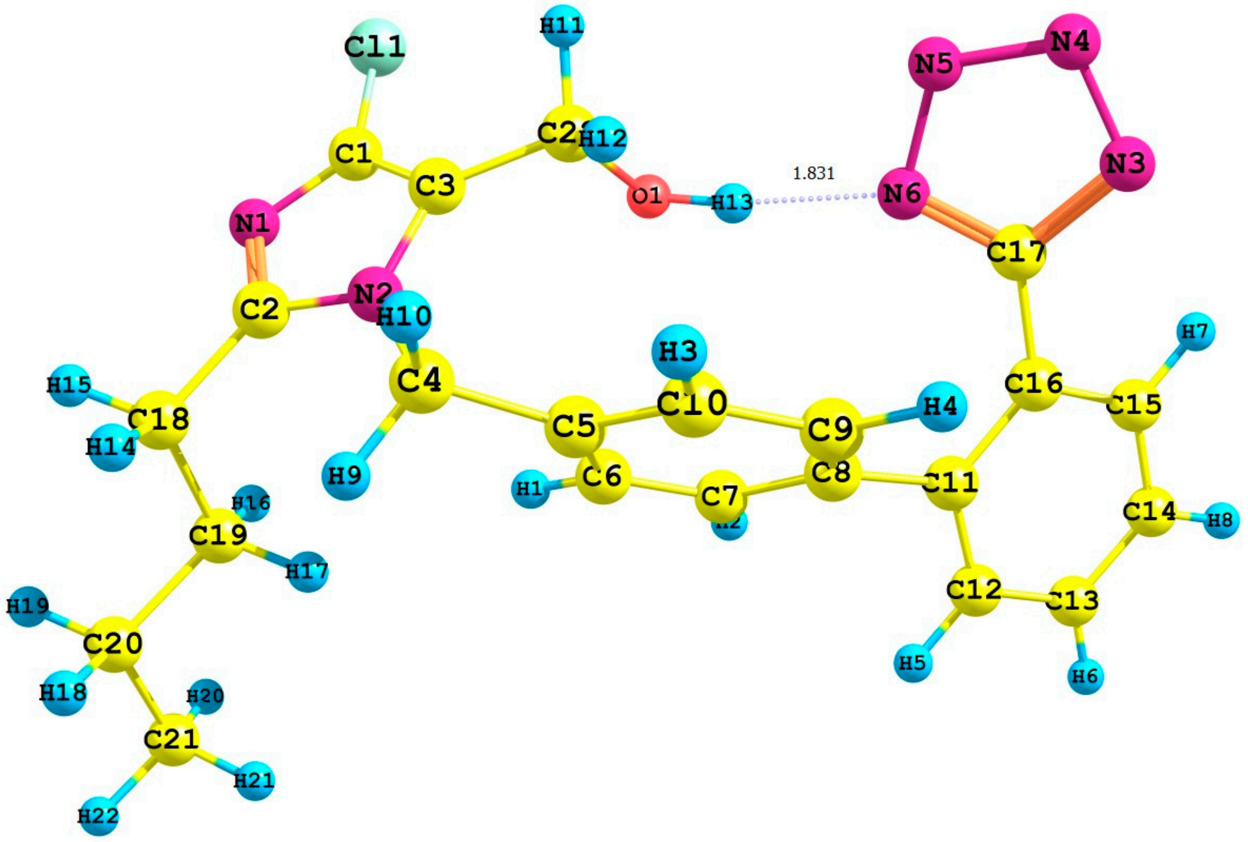

Figure S14. Optimized structure of the *anti*-losartan anion **1** (PBE1PBE/6-31G(d,p)/CPCM level of theory, Rotamer **XIV**).

Cartesian coordinates:

C	3.108872000	−2.248987000	−0.572774000
N	4.233973000	−1.497138000	−0.523221000
C	3.918305000	−0.480233000	0.263268000
N	2.627331000	−0.568395000	0.698019000
C	2.079504000	−1.729487000	0.169387000
C	1.992739000	0.323190000	1.665904000
C	0.630611000	0.792870000	1.225747000
C	0.437550000	1.368157000	−0.029516000
C	−0.843879000	1.661680000	−0.478572000
C	−1.960290000	1.370667000	0.311657000
C	−1.755401000	0.878396000	1.603868000
C	−0.474067000	0.594597000	2.054823000
C	−3.329949000	1.558375000	−0.222131000
C	−3.703168000	2.820593000	−0.699377000
C	−4.985313000	3.069488000	−1.175303000
C	−5.924294000	2.041024000	−1.179800000
C	−5.563116000	0.777341000	−0.730349000
C	−4.271068000	0.505959000	−0.256186000

C	−3.984341000	−0.881373000	0.153644000
N	−4.929974000	−1.676216000	0.674292000
N	−4.332511000	−2.853208000	0.855864000
N	−3.084372000	−2.782600000	0.457902000
N	−2.829781000	−1.552942000	0.009439000
C	4.860390000	0.630656000	0.592318000
C	4.620695000	1.910651000	−0.218171000
C	5.648302000	2.995893000	0.084610000
C	5.415873000	4.268131000	−0.720278000
C	0.671489000	−2.208219000	0.318467000
O	−0.129037000	−1.723126000	−0.733556000
Cl	3.056180000	−3.706971000	−1.503772000
H	1.288294000	1.536397000	−0.684945000
H	−0.987556000	2.064714000	−1.477479000
H	−0.333024000	0.171775000	3.046837000
H	−2.610391000	0.678247000	2.242832000
H	−2.971675000	3.623781000	−0.668895000
H	−5.250164000	4.060636000	−1.531640000
H	−6.280733000	−0.036712000	−0.751637000
H	−6.932161000	2.218308000	−1.543830000
H	2.671006000	1.167176000	1.811295000
H	1.915138000	−0.193872000	2.627926000
H	0.708117000	−3.309285000	0.313010000
H	0.280613000	−1.914125000	1.299924000
H	−1.047687000	−1.567155000	−0.409388000
H	4.835289000	0.858080000	1.665194000
H	5.865105000	0.252992000	0.379509000
H	4.646560000	1.663039000	−1.287104000
H	3.613399000	2.299917000	−0.017831000
H	5.623773000	3.227362000	1.157736000
H	6.654031000	2.605946000	−0.120562000
H	5.466015000	4.068680000	−1.796302000
H	4.428974000	4.693882000	−0.508315000
H	6.165001000	5.031016000	−0.487237000

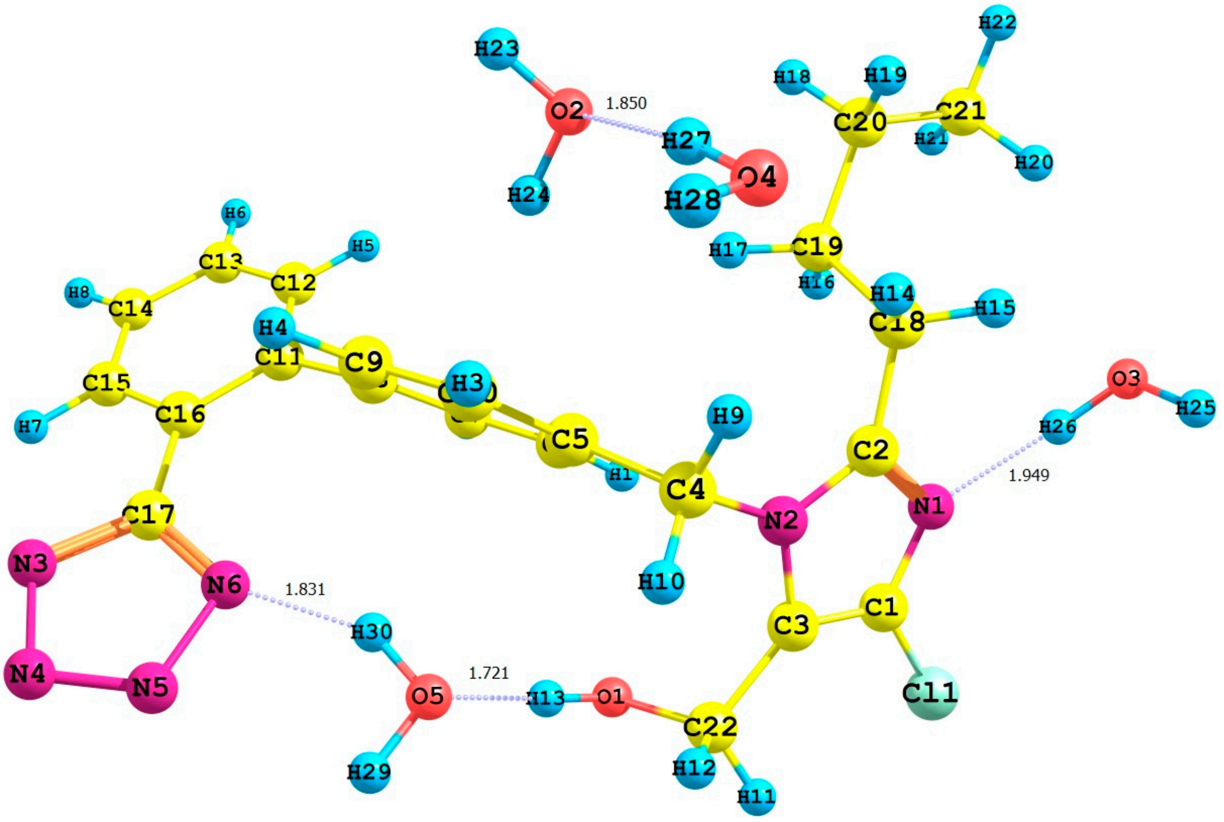

Figure S15. Optimized structure of the *anti*-losartan anion cluster **1** with water molecules (**XV**); interaction of **1** with three water molecules (B3LYP/6-31G(d,p) level of theory, gas).

Cartesian coordinates:

C	−4.247043000	−0.912515000	1.645576000
N	−4.555092000	0.411155000	1.560187000
C	−3.895002000	0.857151000	0.495765000
N	−3.179290000	−0.154165000	−0.090878000
C	−3.401567000	−1.321615000	0.643629000
C	−2.394833000	−0.060903000	−1.319404000
C	−0.909467000	0.202054000	−1.150464000
C	−0.243758000	0.153126000	0.073399000
C	1.148408000	0.269599000	0.122963000
C	1.908939000	0.415911000	−1.041220000
C	1.229476000	0.518335000	−2.265991000
C	−0.157132000	0.414750000	−2.317372000
C	3.398039000	0.453789000	−0.992580000
C	4.011984000	1.527802000	−0.329414000
C	5.397017000	1.662978000	−0.279292000
C	6.194118000	0.703472000	−0.907159000
C	5.604088000	−0.378462000	−1.550029000
C	4.204971000	−0.540591000	−1.602001000
C	3.691684000	−1.756232000	−2.257827000

N	4.477001000	−2.536627000	−3.026919000
N	3.700541000	−3.560584000	−3.396931000
N	2.493571000	−3.413690000	−2.872163000
N	2.455995000	−2.282996000	−2.148016000
C	−3.946715000	2.283706000	0.041371000
C	−2.773109000	3.153168000	0.548577000
C	−3.018669000	4.654665000	0.324170000
C	−3.996317000	5.274284000	1.331997000
C	−2.791319000	−2.661499000	0.346663000
O	−1.424061000	−2.743658000	0.685304000
Cl	−4.925980000	−1.905687000	2.907792000
H	−0.799092000	−0.036661000	0.984339000
H	1.656760000	0.187300000	1.079045000
H	−0.663168000	0.446516000	−3.279858000
H	1.800323000	0.624581000	−3.183300000
H	3.377128000	2.276609000	0.137874000
H	5.845581000	2.507644000	0.236864000
H	6.209113000	−1.142708000	−2.025169000
H	7.277561000	0.791596000	−0.885546000
H	−2.834136000	0.720565000	−1.947430000
H	−2.505792000	−1.010595000	−1.852851000
H	−3.362497000	−3.395128000	0.931493000
H	−2.950399000	−2.912530000	−0.713144000
H	−0.905977000	−2.824539000	−0.153351000
H	−3.989863000	2.346729000	−1.051798000
H	−4.885497000	2.678704000	0.438472000
H	−2.620103000	2.962167000	1.618638000
H	−1.851932000	2.852729000	0.039507000
H	−2.059369000	5.184330000	0.379215000
H	−3.384533000	4.806069000	−0.700213000
H	−4.938722000	4.720555000	1.393948000
H	−3.563080000	5.273140000	2.339158000
H	−4.228759000	6.313779000	1.073152000
O	−0.544403000	3.525390000	−2.307264000
H	0.133346000	3.919214000	−2.870714000
H	−0.182475000	2.656082000	−2.060751000
O	−6.416601000	2.468622000	2.376039000
H	−7.139381000	2.150032000	1.821231000
H	−5.769197000	1.733214000	2.318724000
O	−3.023978000	2.680420000	−3.354455000
H	−2.184623000	3.063994000	−3.027064000
H	−2.768496000	2.186576000	−4.142880000

O	−0.195116000	−2.845784000	−1.720950000
H	0.081349000	−3.690910000	−2.104193000
H	0.680393000	−2.377880000	−1.710230000

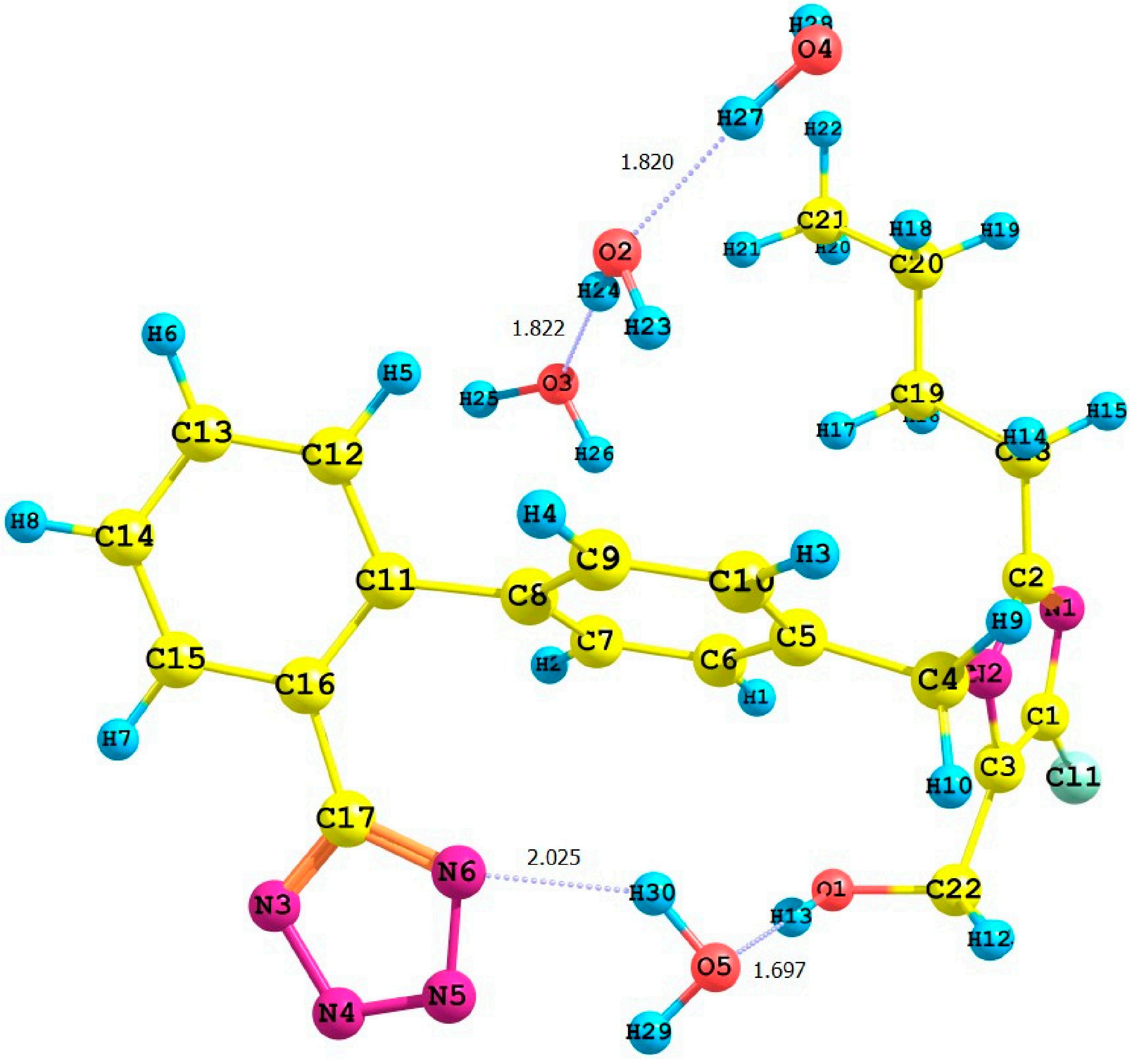

Figure S16. Optimized structure of the *anti*-losartan anion cluster **1** with water molecules (**XVI**); interaction of **1** with three water molecules (PBE1PBE/6-31G(d,p) level of theory, gas).

Cartesian coordinates:

C	−4.913755000	−1.457907000	0.962138000
N	−5.121094000	−0.124155000	0.854451000
C	−4.299648000	0.265787000	−0.101974000
N	−3.585627000	−0.785124000	−0.605753000
C	−3.973158000	−1.926778000	0.080043000
C	−2.665107000	−0.749149000	−1.724137000
C	−1.262221000	−0.291341000	−1.410256000
C	−0.654115000	−0.592548000	−0.188876000

C	0.704395000	−0.361356000	−0.011791000
C	1.490206000	0.174061000	−1.040817000
C	0.861632000	0.534041000	−2.235188000
C	−0.500955000	0.302994000	−2.416248000
C	2.951293000	0.357133000	−0.861837000
C	3.471927000	1.655856000	−0.957087000
C	4.820539000	1.911530000	−0.733728000
C	5.668221000	0.852486000	−0.416104000
C	5.172350000	−0.441479000	−0.356088000
C	3.816357000	−0.725837000	−0.586695000
C	3.407036000	−2.134562000	−0.569173000
N	4.216952000	−3.108454000	−0.122559000
N	3.535552000	−4.231489000	−0.296288000
N	2.364523000	−3.954808000	−0.830664000
N	2.250361000	−2.642411000	−1.018693000
C	−4.124305000	1.690532000	−0.510317000
C	−3.221605000	2.467821000	0.456204000
C	−3.149719000	3.957908000	0.144826000
C	−2.290505000	4.718702000	1.148355000
C	−3.351117000	−3.275837000	−0.086358000
O	−2.062263000	−3.342712000	0.462316000
Cl	−5.792931000	−2.403670000	2.111289000
H	−1.225639000	−1.093583000	0.587230000
H	1.185358000	−0.674582000	0.910983000
H	−0.964047000	0.545894000	−3.370745000
H	1.457776000	0.945769000	−3.045294000
H	2.795988000	2.469986000	−1.215666000
H	5.201700000	2.926245000	−0.808559000
H	5.822198000	−1.283312000	−0.138702000
H	6.724108000	1.032949000	−0.231433000
H	−3.097393000	−0.126801000	−2.514447000
H	−2.605198000	−1.766123000	−2.126165000
H	−4.013083000	−3.986342000	0.427003000
H	−3.349058000	−3.567032000	−1.148600000
H	−1.402457000	−3.450733000	−0.267173000
H	−3.724453000	1.762370000	−1.528413000
H	−5.117589000	2.152669000	−0.523297000
H	−3.602109000	2.314694000	1.473943000
H	−2.212949000	2.034640000	0.433687000
H	−2.749163000	4.121407000	−0.863864000
H	−4.167909000	4.372280000	0.144118000
H	−2.682652000	4.617971000	2.166824000

H	−1.261504000	4.343234000	1.154916000
H	−2.254806000	5.789308000	0.916141000
O	0.568187000	3.691958000	−1.440843000
H	0.302360000	2.848996000	−1.828095000
H	0.662623000	3.478899000	−0.490593000
O	1.028791000	2.676783000	1.103552000
H	1.967571000	2.478042000	0.992338000
H	0.607705000	1.814084000	0.983549000
O	−1.346403000	5.528884000	−2.319008000
H	−0.637259000	4.925602000	−2.023136000
H	−1.495874000	6.082737000	−1.548476000
O	−0.313609000	−3.587556000	−1.561525000
H	0.336576000	−4.276886000	−1.334040000
H	0.301835000	−2.829064000	−1.535962000

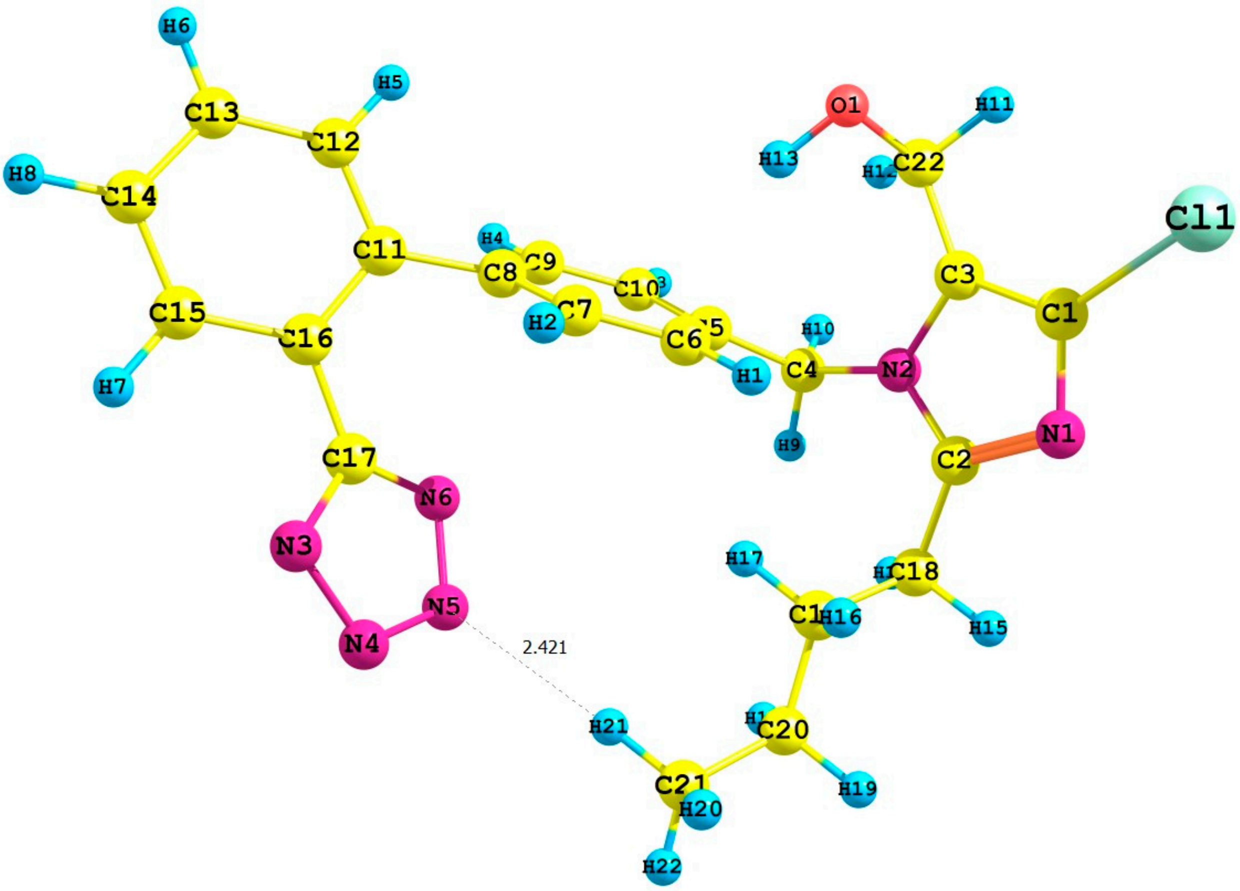

Figure S17. Optimized structure of the *syn*-losartan anion **1** (MP2/6-31G(d,p)/gas level of theory, Rotamer **XVII**).

Cartesian coordinates:

C	−4.388726000	−1.523290000	0.809352000
N	−4.649729000	−0.191416000	0.838852000
C	−3.914850000	0.317147000	−0.157385000

N	−3.213619000	−0.668889000	−0.804015000
C	−3.505312000	−1.878475000	−0.195647000
C	−2.294466000	−0.498655000	−1.918360000
C	−0.859618000	−0.763215000	−1.538971000
C	−0.393661000	−0.467136000	−0.248735000
C	0.965808000	−0.540102000	0.035760000
C	1.888725000	−0.877336000	−0.965385000
C	1.412596000	−1.239079000	−2.230658000
C	0.049561000	−1.174984000	−2.517728000
C	3.337078000	−0.828565000	−0.679892000
C	4.117276000	−1.983833000	−0.826802000
C	5.474477000	−1.977310000	−0.506461000
C	6.059263000	−0.801650000	−0.026722000
C	5.299196000	0.359464000	0.096346000
C	3.932567000	0.373962000	−0.231953000
C	3.185509000	1.627796000	−0.127310000
N	3.489777000	2.586111000	0.779425000
N	2.604676000	3.574773000	0.532045000
N	1.807976000	3.212004000	−0.502884000
N	2.157352000	1.981165000	−0.935841000
C	−3.819764000	1.772825000	−0.476265000
C	−2.538651000	2.440375000	0.043037000
C	−2.507020000	3.941079000	−0.230148000
C	−1.278987000	4.616342000	0.372165000
C	−2.871074000	−3.177080000	−0.555851000
O	−1.662186000	−3.455886000	0.145577000
Cl	−5.143051000	−2.613844000	1.913928000
H	−1.093030000	−0.171302000	0.524251000
H	1.329432000	−0.271079000	1.019788000
H	−0.307209000	−1.419015000	−3.513450000
H	2.120784000	−1.510704000	−3.004731000
H	3.639948000	−2.897235000	−1.165977000
H	6.063574000	−2.880574000	−0.613629000
H	5.740358000	1.283152000	0.448899000
H	7.111798000	−0.786125000	0.232446000
H	−2.406863000	0.528784000	−2.266526000
H	−2.606078000	−1.152970000	−2.736685000
H	−3.558873000	−3.977195000	−0.283868000
H	−2.720518000	−3.227972000	−1.641270000
H	−1.045280000	−2.740117000	−0.067550000
H	−3.915938000	1.940758000	−1.553744000
H	−4.687961000	2.238985000	−0.007335000

H	−2.473419000	2.262795000	1.120361000
H	−1.652874000	1.979828000	−0.401549000
H	−2.525470000	4.103994000	−1.312140000
H	−3.422344000	4.393720000	0.166298000
H	−1.277774000	4.500917000	1.457042000
H	−0.352927000	4.186682000	−0.012364000
H	−1.278073000	5.685027000	0.153706000

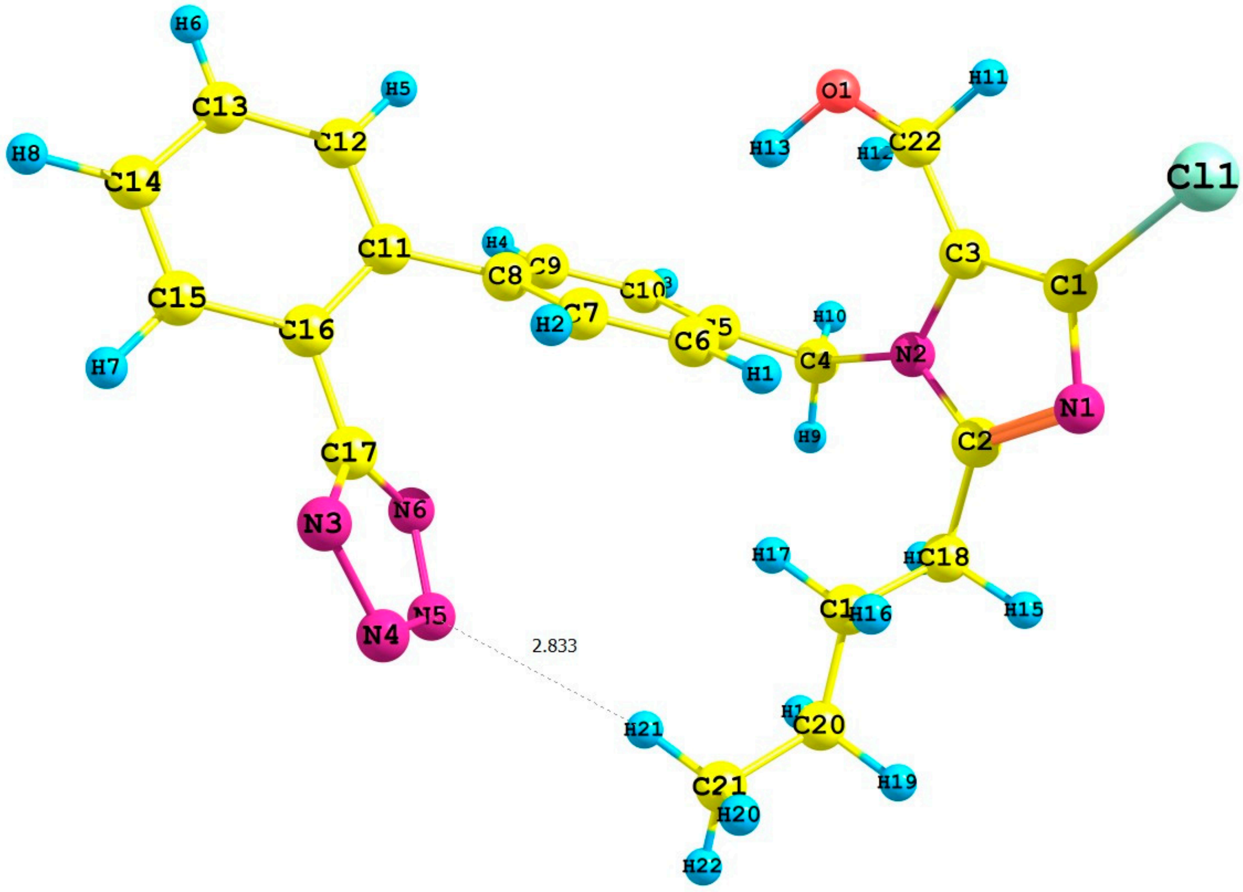

Figure S18. Optimized structure of the *syn*-losartan anion **1** (MP2/6-31G(d,p)/CPCM level of theory, Rotamer **XVIII**).

Cartesian coordinates:

C	−4.226282000	−1.537166000	0.933576000
N	−4.563108000	−0.221220000	0.940004000
C	−3.916106000	0.297303000	−0.112857000
N	−3.193726000	−0.666657000	−0.765814000
C	−3.381277000	−1.871368000	−0.109712000
C	−2.329859000	−0.479913000	−1.923784000
C	−0.870723000	−0.658276000	−1.577313000
C	−0.386149000	−0.335119000	−0.299893000
C	0.977366000	−0.414059000	−0.024343000
C	1.889297000	−0.789808000	−1.023449000

C	1.397600000	−1.142686000	−2.288110000
C	0.031388000	−1.073272000	−2.563453000
C	3.336893000	−0.835390000	−0.728113000
C	4.062309000	−2.010837000	−0.973203000
C	5.414562000	−2.100739000	−0.641395000
C	6.057119000	−1.010396000	−0.048859000
C	5.351567000	0.170919000	0.180945000
C	3.995344000	0.278524000	−0.160768000
C	3.297425000	1.555516000	0.046054000
N	3.317319000	2.238811000	1.212398000
N	2.572775000	3.342258000	0.972944000
N	2.132398000	3.312928000	−0.305311000
N	2.580138000	2.188754000	−0.908226000
C	−3.922540000	1.744993000	−0.476844000
C	−2.661647000	2.484042000	−0.010967000
C	−2.682342000	3.970150000	−0.350960000
C	−1.458348000	4.701908000	0.190019000
C	−2.699635000	−3.147567000	−0.462618000
O	−1.470892000	−3.352963000	0.244531000
Cl	−4.844620000	−2.628713000	2.119601000
H	−1.075001000	−0.043133000	0.483758000
H	1.343921000	−0.158878000	0.962706000
H	−0.334355000	−1.336921000	−3.549518000
H	2.090276000	−1.444612000	−3.064922000
H	3.550497000	−2.865628000	−1.400364000
H	5.957630000	−3.018426000	−0.829239000
H	5.848041000	1.030155000	0.615289000
H	7.104407000	−1.075322000	0.218567000
H	−2.513990000	0.524294000	−2.305010000
H	−2.630587000	−1.179118000	−2.705432000
H	−3.346033000	−3.975218000	−0.174549000
H	−2.548128000	−3.209741000	−1.544101000
H	−0.867667000	−2.652222000	−0.042332000
H	−4.055774000	1.872972000	−1.554232000
H	−4.799577000	2.181660000	0.003267000
H	−2.564998000	2.355353000	1.071149000
H	−1.775272000	2.027460000	−0.459111000
H	−2.736428000	4.086790000	−1.436617000
H	−3.591780000	4.419877000	0.056518000
H	−1.431294000	4.646275000	1.278921000
H	−0.534814000	4.261972000	−0.187284000
H	−1.472935000	5.755110000	−0.090096000

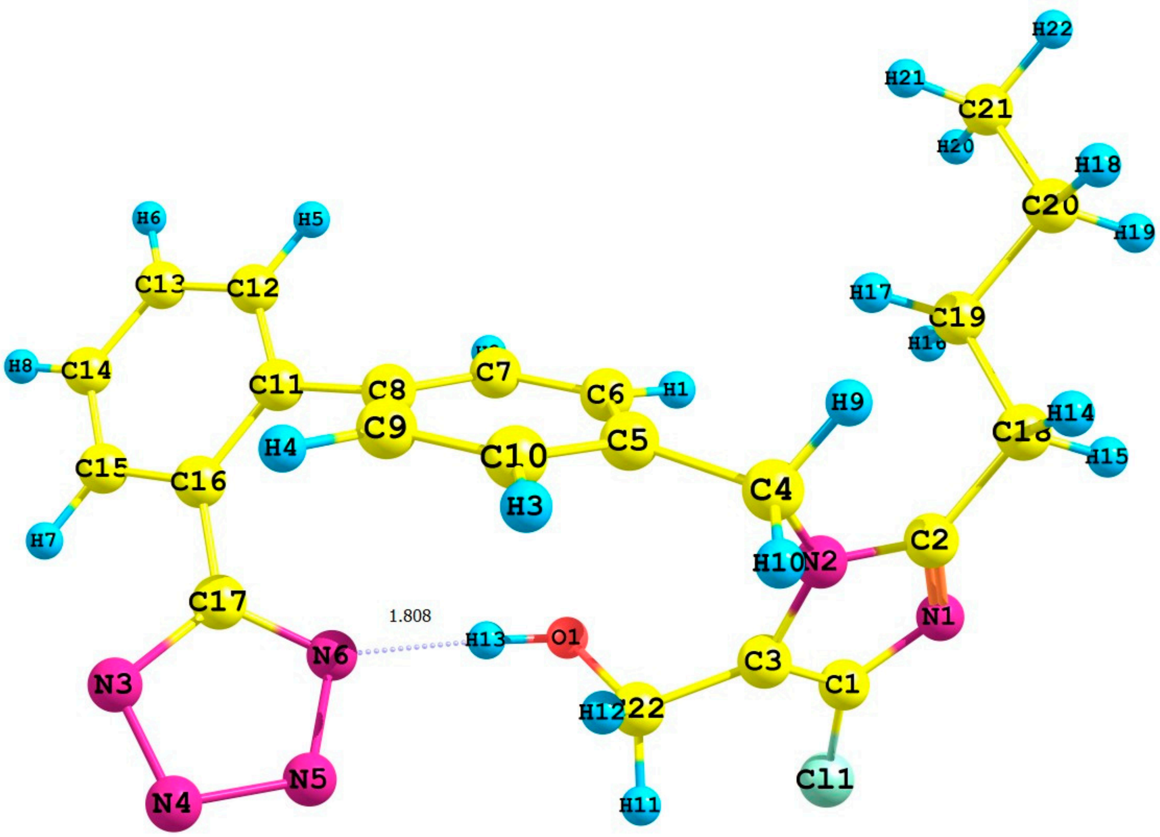

Figure S19. Optimized structure of the *anti*-losartan anion **1** (MP2/6-31G(d,p)/gas level of theory, Rotamer **XIX**).

Cartesian coordinates:

C	−3.561015000	−1.699491000	0.790478000
N	−4.295867000	−0.554958000	0.797180000
C	−3.783572000	0.166189000	−0.204600000
N	−2.748636000	−0.492946000	−0.818913000
C	−2.591485000	−1.725286000	−0.197538000
C	−2.042918000	−0.041817000	−2.015885000
C	−0.548929000	−0.070045000	−1.843312000
C	0.046054000	0.402516000	−0.670654000
C	1.410391000	0.218374000	−0.459050000
C	2.192087000	−0.452054000	−1.408078000
C	1.609464000	−0.835891000	−2.624629000
C	0.248818000	−0.648607000	−2.835976000
C	3.586156000	−0.834370000	−1.108426000
C	4.522156000	0.137407000	−0.728534000
C	5.852386000	−0.202780000	−0.484569000
C	6.258397000	−1.532571000	−0.625340000
C	5.332013000	−2.511879000	−0.979051000
C	3.985852000	−2.187313000	−1.212754000
C	3.052002000	−3.263075000	−1.563864000

N	3.393319000	−4.271586000	−2.396192000
N	2.288697000	−5.046789000	−2.468903000
N	1.317989000	−4.520610000	−1.693213000
N	1.782668000	−3.391517000	−1.111050000
C	−4.225433000	1.552004000	−0.539103000
C	−3.251088000	2.627805000	−0.046049000
C	−3.730736000	4.045344000	−0.339995000
C	−2.759608000	5.108760000	0.160660000
C	−1.499490000	−2.711460000	−0.465107000
O	−0.385598000	−2.390555000	0.343229000
Cl	−3.877458000	−2.961569000	1.924060000
H	−0.572217000	0.816695000	0.117170000
H	1.855093000	0.502115000	0.487763000
H	−0.209583000	−1.014228000	−3.749040000
H	2.211088000	−1.353398000	−3.361872000
H	4.200528000	1.170907000	−0.656335000
H	6.566072000	0.561834000	−0.200692000
H	5.626497000	−3.548954000	−1.078515000
H	7.291518000	−1.807102000	−0.445888000
H	−2.411281000	0.961045000	−2.235817000
H	−2.328631000	−0.682949000	−2.854067000
H	−1.896901000	−3.707413000	−0.234230000
H	−1.242836000	−2.715352000	−1.529277000
H	0.423808000	−2.723491000	−0.122586000
H	−4.394353000	1.669352000	−1.614372000
H	−5.192246000	1.685405000	−0.051000000
H	−3.115763000	2.498568000	1.031223000
H	−2.267607000	2.475978000	−0.500393000
H	−3.876734000	4.159511000	−1.418050000
H	−4.710958000	4.194367000	0.121302000
H	−2.621718000	5.027491000	1.238941000
H	−1.782670000	4.990851000	−0.308512000
H	−3.118996000	6.114432000	−0.057812000

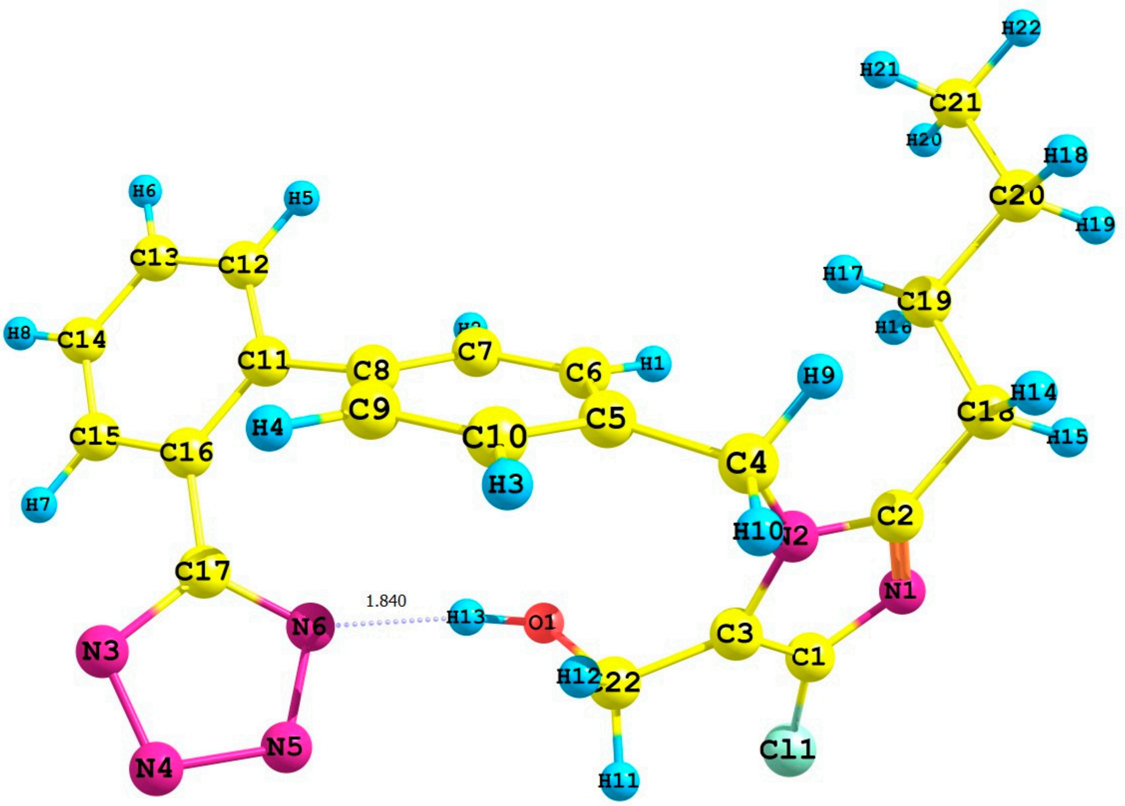

Figure S20. Optimized structure of the *anti*-losartan anion **1** (MP2/6-31G(d,p)/CPCM level of theory, Rotamer **XX**).

Cartesian coordinates:

C	−3.547312000	−1.693400000	0.754258000
N	−4.264680000	−0.540698000	0.796973000
C	−3.769820000	0.193574000	−0.208420000
N	−2.760904000	−0.470733000	−0.856611000
C	−2.606028000	−1.713077000	−0.258873000
C	−2.053859000	−0.001938000	−2.050929000
C	−0.561454000	−0.083008000	−1.873079000
C	0.045466000	0.401767000	−0.709945000
C	1.406595000	0.197701000	−0.493633000
C	2.177305000	−0.503719000	−1.431620000
C	1.583478000	−0.908524000	−2.636088000
C	0.224488000	−0.700704000	−2.852586000
C	3.580578000	−0.856095000	−1.134753000
C	4.499523000	0.146053000	−0.790388000
C	5.836500000	−0.159734000	−0.535415000
C	6.271679000	−1.484635000	−0.623861000
C	5.364624000	−2.494053000	−0.944426000
C	4.014926000	−2.199780000	−1.195216000
C	3.101582000	−3.311771000	−1.499594000

N	3.442332000	−4.332226000	−2.317195000
N	2.374488000	−5.159715000	−2.309971000
N	1.421133000	−4.650590000	−1.502930000
N	1.864013000	−3.485185000	−0.980665000
C	−4.214436000	1.582723000	−0.525451000
C	−3.242760000	2.655491000	−0.018848000
C	−3.742254000	4.071894000	−0.283248000
C	−2.774936000	5.134963000	0.224726000
C	−1.554771000	−2.725690000	−0.565717000
O	−0.454040000	−2.543902000	0.318556000
Cl	−3.841224000	−2.970963000	1.881132000
H	−0.557441000	0.878587000	0.054320000
H	1.863390000	0.523075000	0.434049000
H	−0.238642000	−1.056336000	−3.766584000
H	2.179733000	−1.426330000	−3.378074000
H	4.160558000	1.174915000	−0.750380000
H	6.533344000	0.628689000	−0.280083000
H	5.687537000	−3.526587000	−0.991839000
H	7.308133000	−1.731688000	−0.430866000
H	−2.384593000	1.019145000	−2.235090000
H	−2.366860000	−0.606215000	−2.904420000
H	−2.002363000	−3.717526000	−0.431944000
H	−1.249711000	−2.648920000	−1.610972000
H	0.372155000	−2.786860000	−0.160664000
H	−4.380577000	1.705087000	−1.598913000
H	−5.183057000	1.713727000	−0.040715000
H	−3.093447000	2.511444000	1.054967000
H	−2.264264000	2.524525000	−0.489906000
H	−3.901307000	4.200392000	−1.357097000
H	−4.717172000	4.201186000	0.193891000
H	−2.623175000	5.036762000	1.299781000
H	−1.803756000	5.037034000	−0.260878000
H	−3.149994000	6.138764000	0.027997000

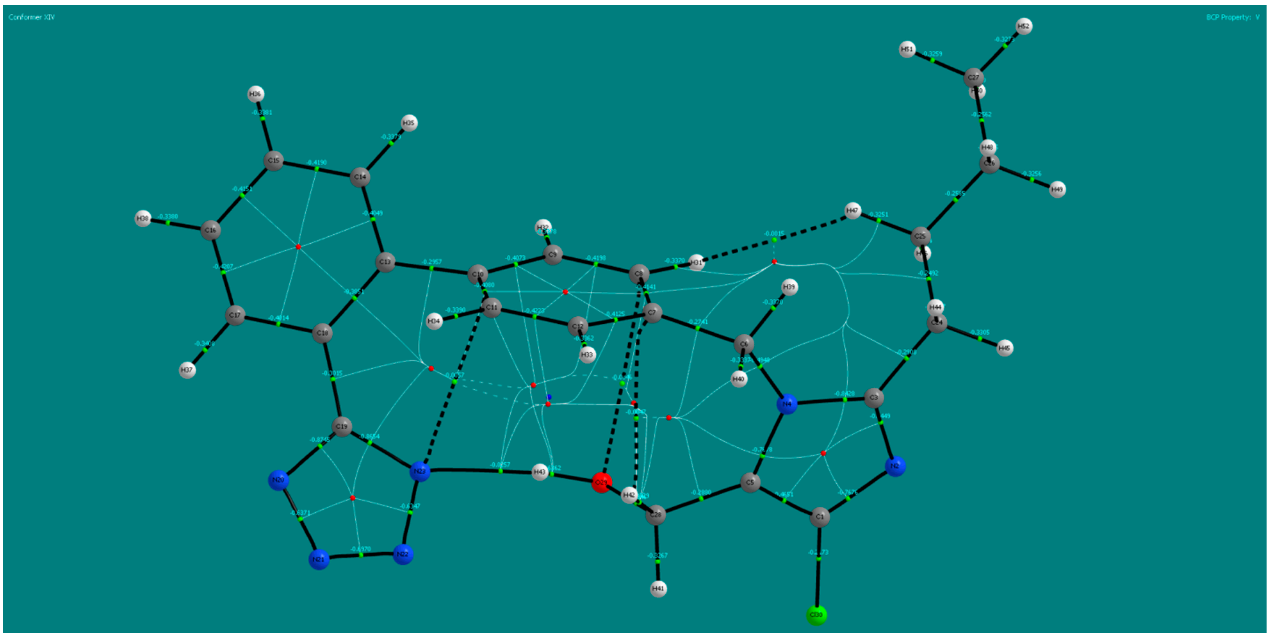

Figure S21. Results of QTAIM (quantum theory of atoms-in-molecules) calculations of the *anti*-losartan anion **1** (PBE1PBE/6-31G(d,p)/CPCM level of theory, Rotamer **XIV**).

Table S19. Data extracted from the *sumviz* type of file (QTAIM calculations).

BCP No.	Name	Atoms	Rho	DelSqRho	Ellipticity	K
1	BCP1	C1–N2	0.338539	−1.11326	0.248911	0.522973
2	BCP2	N2–C3	0.356886	−1.01807	0.287978	0.599693
3	BCP3	N4–C5	0.295841	−0.60569	0.207922	0.459635
4	BCP4	C3–N4	0.314094	−0.66426	0.230813	0.504429
5	BCP5	C1–C5	0.325362	−0.88639	0.413812	0.343367
6	BCP6	N4–C6	0.256977	−0.70186	0.025562	0.335154
7	BCP7	C6–C7	0.257584	−0.62475	0.044061	0.215149
8	BCP8	C7–C8	0.311528	−0.8467	0.20875	0.312909
9	BCP9	C8–C9	0.313449	−0.86124	0.212157	0.317552
10	BCP10	C9–C10	0.308815	−0.83277	0.207404	0.307755
11	BCP11	C7–C12	0.311001	−0.84327	0.214707	0.311675
12	BCP12	C10–C11	0.309353	−0.83724	0.202807	0.308656
13	BCP13	C6–H40	0.28342	−1.01651	0.03347	0.293928
14	BCP14	C7–H42	0.009099	0.032679	2.37448	−0.00172
15	BCP15	C11–C12	0.314457	−0.86662	0.214575	0.319494
16	BCP16	C13–C14	0.307738	−0.82496	0.212194	0.305571
17	BCP17	C10–C13	0.268187	−0.6715	0.048524	0.231774
18	BCP18	C15–H36	0.283952	−1.02516	0.014542	0.297183
19	BCP19	C14–C15	0.313393	−0.86175	0.209923	0.31722
20	BCP20	C15–C16	0.312137	−0.85649	0.202544	0.314592
21	BCP21	C13–C18	0.300063	−0.78219	0.205177	0.290331
22	BCP22	C16–H38	0.283903	−1.02449	0.014445	0.297038
23	BCP23	C16–C17	0.314045	−0.86446	0.21056	0.31841
24	BCP24	C11–N23	0.010037	0.032678	1.130845	−0.00125
25	BCP25	C17–H37	0.285579	−1.04155	0.01604	0.300598

Table S19. *Cont.*

BCP No.	Name	Atoms	Rho	DelSqRho	Ellipticity	K
26	BCP26	C17–C18	0.306919	−0.82336	0.202169	0.303635
27	BCP27	C18–C19	0.270951	−0.69083	0.097104	0.237081
28	BCP28	C19–N23	0.34381	−1.00907	0.221963	0.55885
29	BCP29	C19–N20	0.349835	−1.07069	0.227916	0.571074
30	BCP30	N20–N21	0.390047	−0.78742	0.111266	0.416954
31	BCP31	N22–N23	0.38917	−0.78375	0.124609	0.415327
32	BCP32	N21–N22	0.413605	−0.88451	0.168266	0.459056
33	BCP33	C3–C24	0.261819	−0.65068	0.053432	0.226758
34	BCP34	H31–H47	0.003696	0.012698	0.087341	−0.00084
35	BCP35	C24–C25	0.241004	−0.54169	0.018346	0.192288
36	BCP36	C25–H47	0.275048	−0.92991	0.007341	0.278806
37	BCP37	C26–H49	0.2754	−0.93331	0.005084	0.279455
38	BCP38	C25–C26	0.247302	−0.571	0.015898	0.200634
39	BCP39	C26–C27	0.245993	−0.56704	0.006224	0.198997
40	BCP40	C5–C28	0.262419	−0.65696	0.004286	0.226131
41	BCP41	C8–O29	0.007455	0.024427	2.098968	−0.00077
**42**	**BCP42**	**N23–H43**	**0.038172**	**0.092778**	**0.035852**	**0.00124**
43	BCP43	C28–O29	0.268979	−0.58425	0.055585	0.390307
44	BCP44	C1–Cl30	0.202695	−0.32282	0.080286	0.149008
45	BCP45	C8–H31	0.283224	−1.01885	0.014072	0.295878
46	BCP46	C9–H32	0.283703	−1.02258	0.017135	0.296711
47	BCP47	C12–H33	0.282726	−1.01382	0.016335	0.294815
48	BCP48	C11–H34	0.284446	−1.02933	0.01513	0.298143
49	BCP49	C14–H35	0.28375	−1.02225	0.017544	0.296717
50	BCP50	C6–H39	0.284102	−1.02426	0.028876	0.29654
51	BCP51	C28–H41	0.280105	−0.98221	0.041204	0.28612
52	BCP52	C28–H42	0.283069	−1.00893	0.035947	0.292572
53	BCP53	O29–H43	0.335798	−1.95476	0.024855	0.562452
54	BCP54	C24–H44	0.275791	−0.94265	0.01123	0.280699
55	BCP55	C24–H45	0.278757	−0.97037	0.008075	0.286559
56	BCP56	C25–H46	0.276297	−0.94282	0.006423	0.28104
57	BCP57	C26–H48	0.275351	−0.93281	0.005002	0.279364
58	BCP58	C27–H50	0.274798	−0.93357	0.006738	0.279685
59	BCP59	C27–H51	0.274768	−0.93325	0.006732	0.27963
60	BCP60	C27–H52	0.275481	−0.94027	0.006981	0.281201

In this table, the $N6_{tetrazole}$···HO-CH_2-imidazole type of interactions within the structure of Rotamer **XIV** (isomer *anti*) are marked.

Number of electrons (from occupied molecular orbitals) = 222.0000000000

Number of alpha electrons (from occupied molecular orbitals) = 111.0000000000

Number of beta electrons (from MO Occs) = 111.0000000000

Number of electron pairs (N × (N − 1)/2) = 24,531.0000000000

Number of electron pairs = 24,531.0000000000

Number of NACPs = 52

Number of NNACPs = 0
Number of BCPs = 60
Number of RCPs = 10
Number of CCPs = 1
NumNACP + NumNNACP - NumBCP + NumRCP − NumCCP = 1
Poincare–Hopf Relationship is satisfied.
NACP = nuclear attractor critical point
NNACP = non-nuclear attractor critical point
BCP = bond critical point
RCP = ring critical point
CCP = cage critical point rho = electron density
DelSqRho = Laplacian of rho = trace of Hessian of rho
Bond ellipticity = (HessRho_EigVal(1)/HessRho_EigVal(2)) − 1
K = Hamiltonian form of kinetic energy density

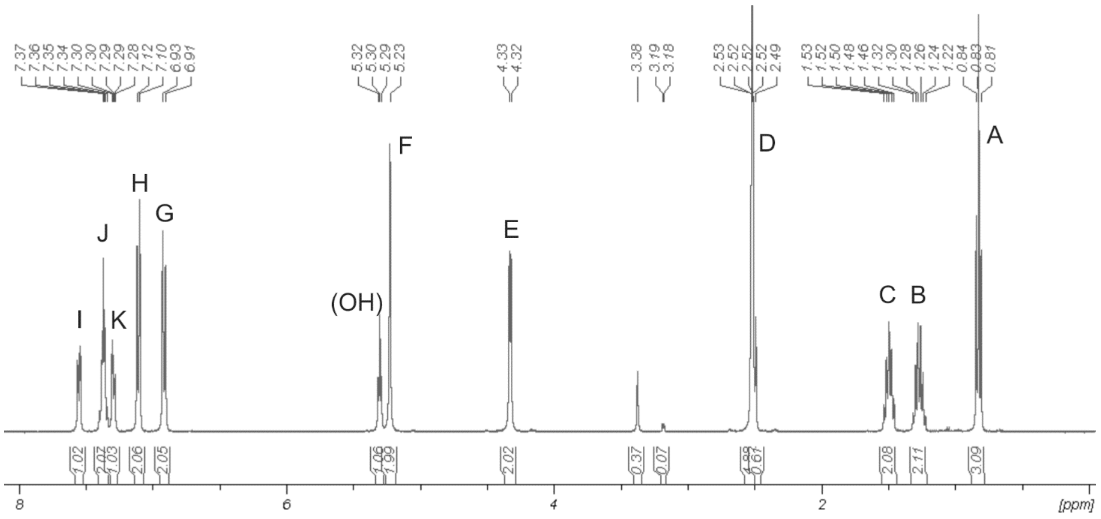

Figure S22. The experimental ^{1}H-NMR spectrum of Losartan **1** recorded at 293 K.

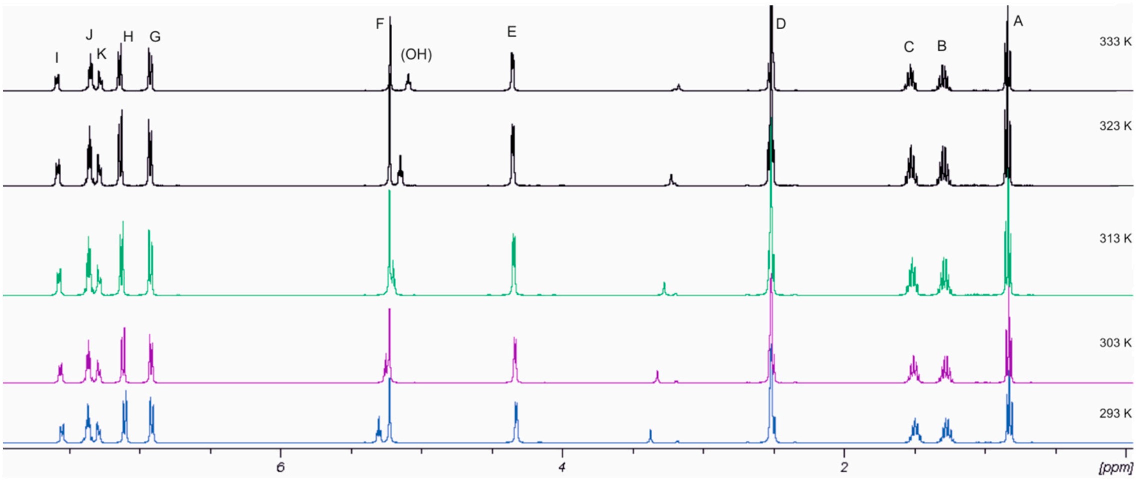

Figure S23. The imposition of experimental ^{1}H-NMR spectra of Losartan **1** recorded at five different temperatures.

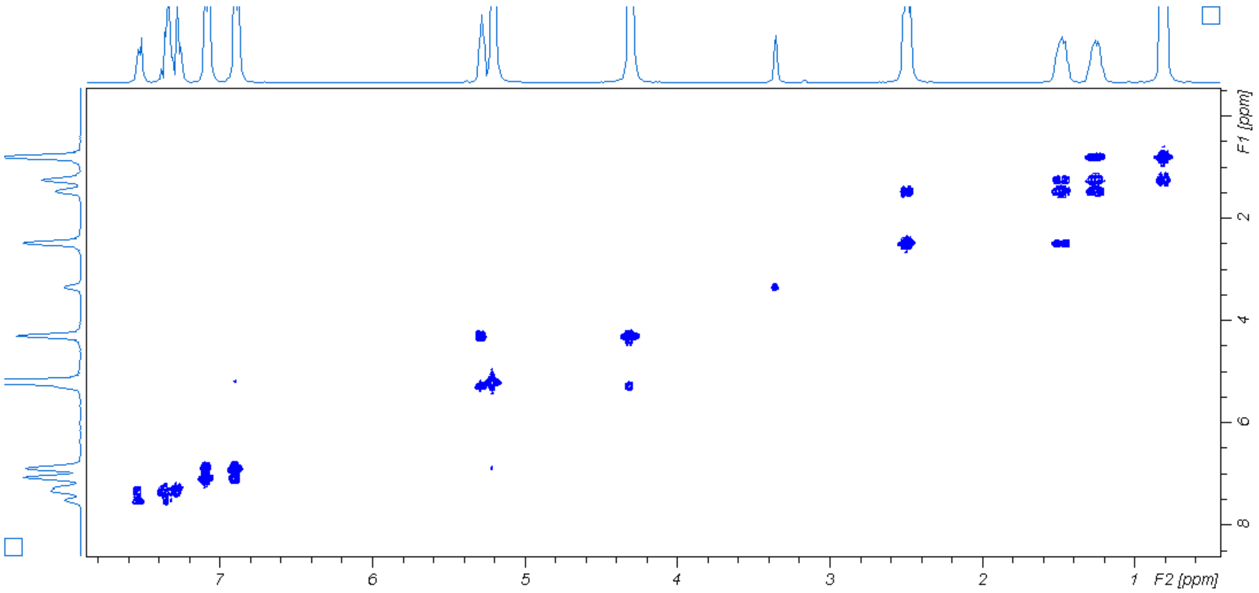

Figure S24. The ^{1}H-^{1}H COSY spectrum of Losartan **1** recorded at 293 K.

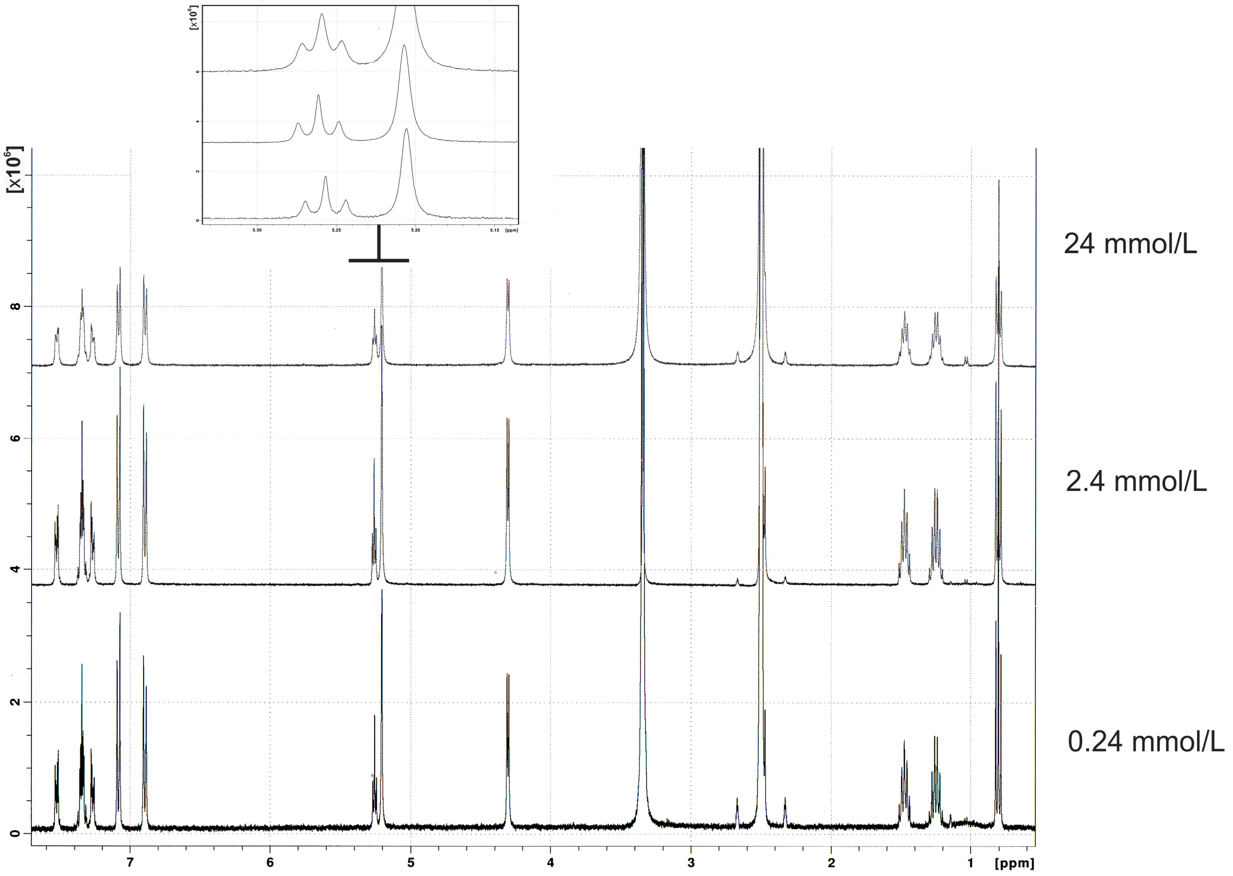

Figure S25. The imposition of experimental ^{1}H NMR spectra of Losartan **1** recorded at three different concentrations.
